# *In vitro* expanded skeletal myogenic progenitors from pluripotent stem cell-derived teratomas have high engraftment capacity

**DOI:** 10.1016/j.stemcr.2021.10.014

**Published:** 2021-11-18

**Authors:** Ning Xie, Sabrina N. Chu, Karim Azzag, Cassandra B. Schultz, Lindsay N. Peifer, Michael Kyba, Rita C.R. Perlingeiro, Sunny S.K. Chan

**Affiliations:** 1 Department of Pediatrics, University of Minnesota, 2231 6th Street SE, Cancer and Cardiovascular Research Building, Minneapolis, MN 55455 USA; 2 Stem Cell Institute, University of Minnesota, Minneapolis, MN, USA; 3 Lillehei Heart Institute, University of Minnesota, Minneapolis, MN, USA; 4 Department of Medicine, University of Minnesota, Minneapolis, MN, USA

**Keywords:** pluripotent stem cells, myogenic differentiation, muscle stem cells, cell therapy, muscular dystrophy

## Abstract

One major challenge in realizing cell-based therapy for treating muscle-wasting disorders is the difficulty in obtaining therapeutically meaningful amounts of engraftable cells. We have previously described a method to generate skeletal myogenic progenitors with exceptional engraftability from pluripotent stem cells via teratoma formation. Here, we show that these cells are functionally expandable *in vitro* while retaining their *in vivo* regenerative potential. Within 37 days in culture, teratoma-derived skeletal myogenic progenitors were expandable to a billion-fold. Similar to their freshly sorted counterparts, the expanded cells expressed PAX7 and were capable of forming multinucleated myotubes *in vitro*. Importantly, these cells remained highly regenerative *in vivo*. Upon transplantation, the expanded cells formed new DYSTROPHIN^+^ fibers that reconstituted up to 40% of tibialis anterior muscle volume and repopulated the muscle stem cell pool. Our study thereby demonstrates the possibility of producing large quantities of engraftable skeletal myogenic cells for transplantation.

## Highlights

• Skeletal myogenic progenitors from PSC-derived teratomas are expandable *in vitro*
• Expanded cells are PAX7^+^ and are capable of differentiating into MHC^+^ myotubes
• Expanded cells engraft and regenerate fibers with adult myosins and innervation
• Expanded cells repopulate the muscle stem cell niche after transplantation

## Graphical abstract

**Figure:**
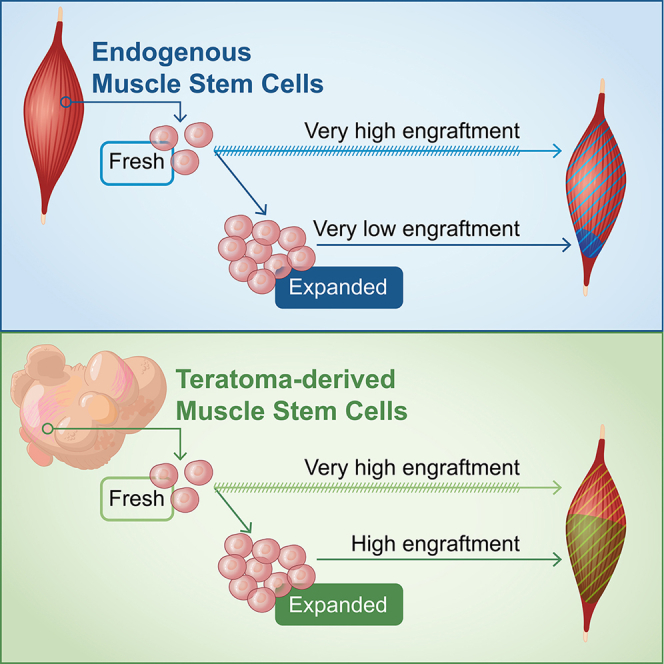

## Teaser

In this article, Chan and colleagues show that skeletal myogenic progenitors obtained from PSC-derived teratomas are expandable in cultures and remain highly regenerative. Upon transplantation, the expanded cells robustly engraft to form new muscle fibers and reconstitute the muscle stem cell pool. This study is an advance toward generating large quantities of engraftable skeletal myogenic progenitors for downstream applications.

## Introduction

Satellite cells, also known as muscle stem cells, are the primary cells responsible for muscle regeneration (Günther et al., 2013; von Maltzahn et al., 2013). Satellite cells reside between the basal lamina and the sarcolemma of muscle fibers (Mauro, 1961), are normally quiescent, and express the transcription factor Pax7 (Schultz et al., 1978; von Maltzahn et al., 2013). In response to muscle injuries, satellite cells are activated, reenter the cell cycle, rapidly proliferate, differentiate into myoblasts, and fuse to form multinucleated myofibers (Cooper et al., 1999; Snow, 1977). In addition, some satellite cells become quiescent again and repopulate the muscle stem cell pool (Zammit et al., 2004). Satellite cells have tremendous *in vivo* regenerative capability. A single satellite cell is capable of regenerating damaged skeletal muscles by reconstituting both the fiber and the muscle stem cell compartments (Collins et al., 2005; Sacco et al., 2008). Therefore, transplantation of healthy satellite cells is a promising approach for treating skeletal muscle-wasting disorders such as Duchenne muscular dystrophy.

Generating sufficient engraftable cells is a major challenge for realizing cell-based therapy (Blau and Daley, 2019). Satellite cells are scarce, representing only 1% to 2% of mononuclear cells in skeletal muscles (Roth et al., 2000). A therapeutically meaningful amount of satellite cells is thereby unlikely to be obtained from small skeletal muscle biopsies. This problem can be theoretically solved by *in vitro* expansion of satellite cells. However, the robust regenerative potency of satellite cells is lost once they are isolated and grown in cultures. For mouse satellite cells, a 3-day culture led to a 10-fold decrease in engraftment potential (Montarras et al., 2005; Sacco et al., 2008). Cultured canine myoblasts produced poorer engraftment compared with freshly isolated satellite cells (Parker et al., 2012). Human myoblasts expanded *in vitro* also showed low engraftment efficiency and failed to replenish the muscle stem cell pool after transplantation into Duchenne muscular dystrophy patients (Gussoni et al., 1992; Mendell et al., 1995). Optimization of the culturing conditions can alleviate this problem to a certain extent. Satellite cells cultured in hydrogels or artificial niches that resemble the elasticity of the native skeletal muscle environment had improved engraftability (Gilbert et al., 2010; Quarta et al., 2016). Similarly, modulation of the Notch and p38 signaling pathways restored the regenerative potential of cultured satellite cells after transplantation (Charville et al., 2015; Parker et al., 2012). However, these approaches were usually performed within a relatively short period of time in cultures. The validity of long-term *in vitro* expansion of engraftable skeletal myogenic cells remains unresolved.

Pluripotent stem cells (PSCs), including embryonic stem cells (ESCs) and induced pluripotent stem cells (iPSCs), possess great promise for cell therapy targeting degenerating muscles (Chal and Pourquié, 2017). PSCs have theoretically unlimited proliferative potential, thereby allowing generation of sufficient skeletal myogenic progenies for transplantations. Currently there are two main approaches to derive skeletal myogenic cells from PSCs *in vitro* (Chal and Pourquié, 2017; Jiwlawat et al., 2018). Overexpression of skeletal myogenic transcription factors such as PAX3 or PAX7 can efficiently differentiate PSCs into skeletal myogenic progenitors that engraft to form force-generating muscle fibers (Darabi et al., 2012; Filareto et al., 2013). On the other hand, non-transgenic approaches employ various growth factors and small molecules to direct skeletal myogenic differentiation from PSCs (Chal et al., 2015; Shelton et al., 2014). However, skeletal myogenic cells derived from monolayer differentiation do not reliably engraft without further purification, making evaluation of the functionality of the regenerated muscles difficult (Hicks et al., 2018). Also, whether monolayer differentiation-derived skeletal myogenic cells can be passaged and expanded while maintaining their already modest engraftability remains unclear (Al Tanoury et al., 2020).

We recently developed a novel method to differentiate mouse PSCs into skeletal myogenic progenitors via teratoma formation (Chan et al., 2018). On a cell-to-cell basis, teratoma-derived skeletal myogenic progenitors are functionally similar to endogenous satellite cells in forming muscle fibers. Also, these teratoma-derived cells repopulate the muscle stem cell pool and are responsive to subsequent injuries for a secondary regeneration. Here, we show that teratoma-derived skeletal myogenic progenitors are readily expandable *in vitro* while retaining significant parts of their robust *in vivo* regenerative capacity.

## Results

### Teratoma-derived skeletal myogenic progenitors are expandable *in vitro*

**Figure 1:**
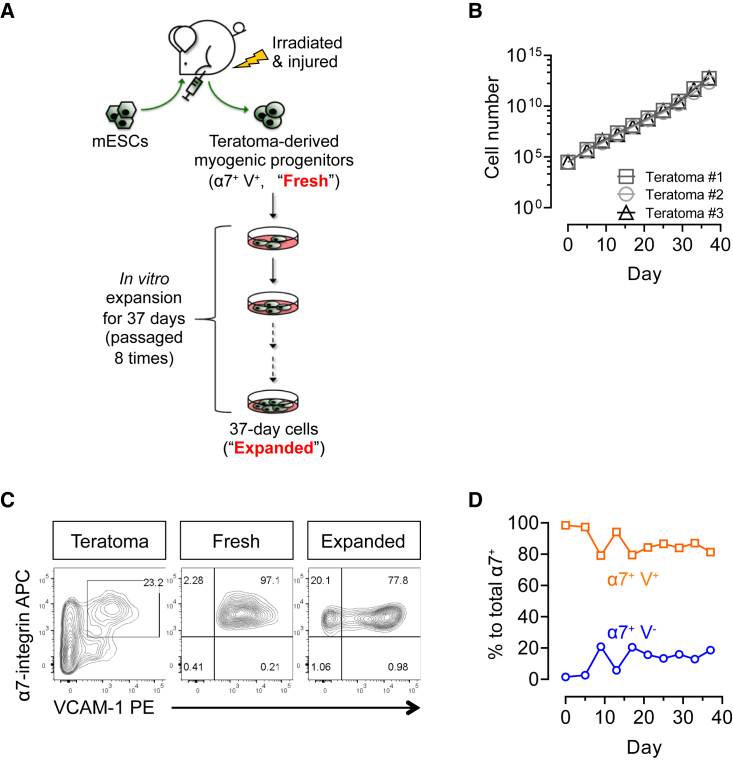
Teratoma-derived skeletal myogenic progenitors are expandable *in vitro* (A) Schematic of *in vitro* expansion of teratoma-derived skeletal myogenic progenitors. Four-week EGFP-labeled mouse ESC-derived teratomas were harvested and FACS sorted for the skeletal myogenic population: CD31^−^ CD45^−^ α7-integrin^+^ VCAM-1^+^ (α7^+^ V^+^) cells. The freshly sorted α7^+^ V^+^ cells (“Fresh”) were subsequently cultured and expanded for another 37 days over 8 passages (“Expanded”). (B) *In vitro* cultures of α7^+^ V^+^ cells showed exponential growth for up to 37 days. Data are shown as the mean ± SEM of three independent experiments. (C) FACS profiling of total teratoma cells, freshly sorted α7^+^ V^+^ cells, and expanded 37-day cells (representative of three independent experiments). (D) Quantitation of α7^+^ V^+^ and α7^+^ V^−^ cells by FACS during *in vitro* expansion. Data are shown as the mean ± SEM of three independent experiments. ESCs, embryonic stem cells; α7, α7-integrin; V, VCAM-1. See also Figure S1.

We have previously reported that mouse PSC-derived teratomas are rich in skeletal myogenic progenitors with exceptional regenerative potency (Chan et al., 2018). Because it is more feasible to expand a purified skeletal myogenic population than to perform a large-scale differentiation operation followed by a massive purification step for each transplant, it is imperative to explore the *in vitro* expandability of teratoma-derived skeletal myogenic progenitors (Figure 1A). We first induced teratoma formation by implanting EGFP-labeled mouse ESCs (E14-EGFP ESCs) into irradiated and cardiotoxin-injured tibialis anterior (TA) muscles of NSG-mdx^4Cv^ mice as previously described (Chan et al., 2018). At 4 weeks, teratomas were harvested and EGFP^+^ (teratoma-derived) skeletal myogenic progenitors, defined as lineage-negative (Lin^−^) (CD31^−^ and CD45^−^, i.e., non-endothelial and non-hematopoietic, respectively), α7-integrin^+^, and VCAM-1^+^ (α7^+^ VCAM^+^), were isolated by fluorescence-activated cell sorting (FACS) (Figures 1A and S1A) (Chan et al., 2018). We then plated and cultured these freshly sorted cells in a pro-proliferation medium (Figure 1A). Teratoma-derived skeletal myogenic progenitors maintained a steady growth and were amplified by 9 orders of magnitude within 37 days over eight passages (Figure 1B). We subsequently compared the skeletal myogenic characteristics of the passage 8 cells (expanded cells) with their freshly sorted counterparts (fresh cells).

We first determined whether the expression of α7 and VCAM was altered during the expansion process (Figures 1C and S1B). From the time points we investigated (up to day 37, or passage 8), most cells stably expressed α7 (98.1%–99.1%, 95% confidence interval; n = 3 independent experiments). This suggested that our culture system promoted a predominately skeletal myogenic population with limited non-myogenic potential (e.g., brown adipocytes [Seale et al., 2008]). Among the α7^+^ population, a majority (≥75%) were also VCAM^+^ (Figure 1D), suggesting a muscle stem cell signature (Chan et al., 2013). Indeed, compared with α7^+^ VCAM^−^ resorted cells, α7^+^ VCAM^+^ cells sorted from passage 8 cultures expanded more readily and had a higher expression of muscle stem cell factor *Pax7* but a lower expression of skeletal muscle specification factors *Myod1* and *Myog* (Figure S1C and S1D). Importantly, α7^+^ VCAM^+^ resorted from passage 8 cultures engrafted more reliably than α7^+^ VCAM^−^ cells (Figure S1E). On the other hand, α7^+^ VCAM^−^ cells may represent a skeletal myogenic-committed subpopulation that was differentiating into myoblasts (Giordani et al., 2019).

### Expanded teratoma-derived α7^+^ VCAM^+^ cells remain highly skeletal myogenic

**Figure 2:**
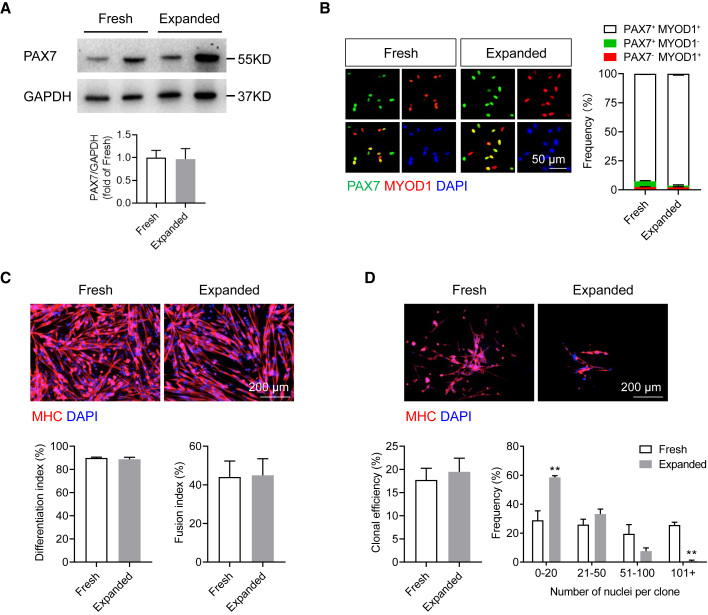
*In vitro* characterization of expanded teratoma-derived skeletal myogenic progenitors (A) Immunoblots (top) and quantification (bottom) showing that both freshly sorted and expanded cells expressed the muscle stem cell transcription factor PAX7 (two independent samples from each group are shown). Data are shown as the mean ± SEM of five independent experiments. (B) Immunostaining (left) and quantification (right) showing the expression of PAX7 and MYOD1 in 3-day cultures of freshly sorted and expanded cells. Scale bar represents 50 μm. Data are shown as the mean ± SEM of three independent experiments. (C) Immunostaining (top) of MHC in freshly sorted and expanded cells cultured in differentiation medium. Scale bar represents 200 μm. Quantification of differentiation (bottom left) and fusion in MHC^+^ myotubes (bottom right) from three independent experiments. Data are shown as the mean ± SEM. (D) Clonal analysis (top) showing that both single freshly sorted and expanded cells were capable of forming MHC^+^ skeletal myogenic colonies. Scale bar represents 200 μm. Quantification of clonal efficiency (bottom left) and clonal size distribution (bottom right) from three independent experiments and 170 single cells per experiment. ^∗∗^p < 0.01. Data are shown as the mean ± SEM. α7, α7-integrin; V, VCAM-1; MHC, myosin heavy chain. See also Figure S2.

Quiescent satellite cells in adult skeletal muscles express the transcription factor PAX7 (von Maltzahn et al., 2013). We observed comparable expression of the PAX7 protein in both freshly sorted and expanded teratoma-derived α7^+^ VCAM^+^ cells, suggesting that the expanded cells remained mostly undifferentiated (Figure 2A). In contrast, PAX7 was significantly reduced in endogenous satellite cells after *in vitro* expansion for eight passages (Figures S2A and S2B).

Once isolated and in culture (or in response to injury *in vivo*), satellite cells become activated and begin to express the skeletal myogenic specification factor MYOD1 (Brack et al., 2008; Tierney et al., 2016). Indeed, after 3 days of culture, cells in both groups started to express MYOD1 (Figure 2B). A majority of them were PAX7^+^ MYOD1^+^ (fresh, 92.7% ± 0.6%; expanded, 96.4% ± 1.3%; n = 3 independent experiments, p > 0.05), indicating a predominately activated/proliferating population as expected. Notably, a small but significant subpopulation of cells remained in an inactivated PAX7^+^ MYOD1^−^ state (fresh, 5.0% ± 0.6%; expanded, 1.6% ± 0.7%; n = 3 independent experiments, p < 0.05). PAX7^−^ MYOD1^+^-committed myoblasts were also observed (fresh, 2.4% ± 0.3%; expanded, 2.0% ± 0.6%; n = 3 independent experiments, p > 0.05).

We next probed the skeletal myogenic differentiation potential of these cells. In serum-depleted conditions, proliferating skeletal myogenic progenitors differentiated into myosin heavy chain-positive (MHC^+^) myoblasts, which subsequently fused into multinucleated myotubes. We found that both freshly sorted and expanded cells were equally adept at developing into myoblasts and fusing into myotubes (Figure 2C).

In culture, single satellite cells are capable of forming individual colonies (Seale et al., 2001). To test the clonality of teratoma-derived skeletal myogenic progenitors, we seeded single freshly sorted and expanded cells by FACS into 96-well plates and cultured them in a medium that supports both proliferation and differentiation (Ippolito et al., 2012). After 8 days, we observed a similar clonal efficiency between the two groups (Figure 2D). Nevertheless, colonies from freshly sorted cells appeared to consist of more myonuclei than expanded cells (Figure 2D). Therefore, on a cell-to-cell basis, freshly sorted cells and expanded cells have comparable proficiency in forming MHC^+^ colonies, although colonies from the latter cells were at a smaller size. Altogether, our results showed that *in vitro* expansion did not fundamentally alter the skeletal myogenic nature of teratoma-derived α7^+^ VCAM^+^ cells.

### Expanded teratoma-derived skeletal myogenic progenitors engraft and differentiate into muscle fibers upon transplantation

**Figure 3:**
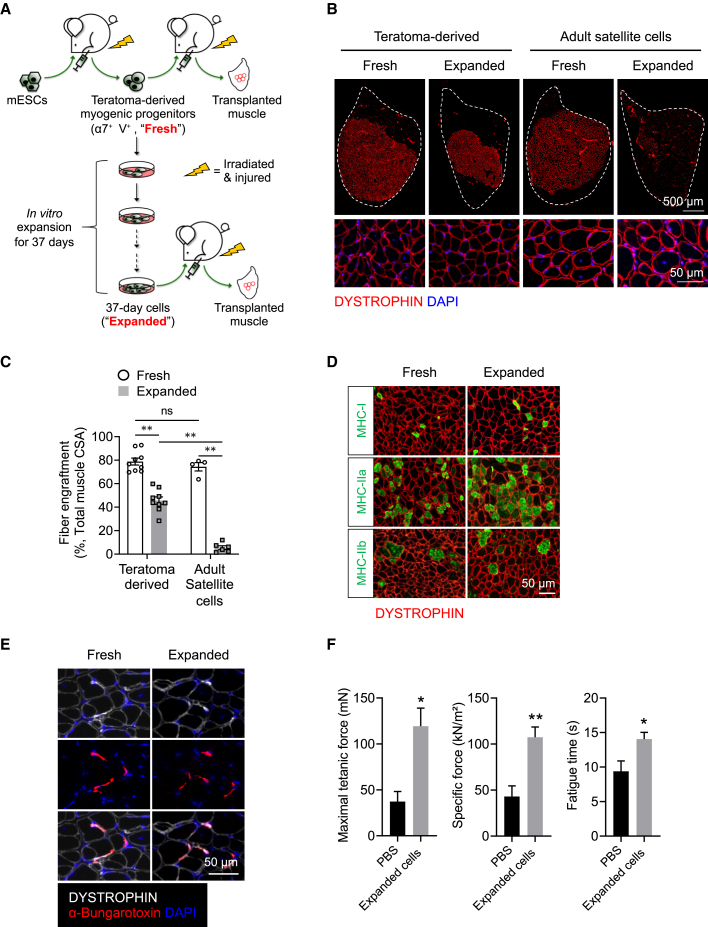
Expanded teratoma-derived skeletal myogenic progenitors engraft and form new muscle fibers (A) Schematic of evaluation of the engraftability of freshly sorted and expanded teratoma-derived skeletal myogenic progenitors. (B) Freshly sorted (far left) and expanded (middle left) teratoma-derived skeletal myogenic progenitors engrafted and formed DYSTOPHIN^+^ fibers 4 months post-transplant. In contrast, freshly sorted adult satellite cells (middle right) engrafted, but their expanded counterparts (far right) did not. The whole TA muscle is outlined (top, scale bar represents 500 μm), and magnified images are shown (bottom, scale bar represents 50 μm). Representative images from four to nine biological replicates. (C) Quantification of fiber engraftment (DYSTROPHIN^+^ fibers) in transplanted TA muscles (n = 4–9 biological replicates). Data are shown as the mean ± SEM. ^∗∗^p < 0.01; ns, not significant. (D) Newly formed DYSTROPHIN^+^ muscle fibers derived from freshly sorted and expanded cells consisted of slow-twitch (MHC-I) and fast-twitch (MHC-IIa and MHC-IIb) fibers (representative images from six biological replicates). Scale bar represents 50 μm. (E) Potential presence of neuromuscular junctions as revealed by close proximity of α-bungarotoxin staining to newly formed DYSTROPHIN^+^ fibers derived from freshly sorted and expanded cells (representative images from three biological replicates). Scale bar represents 50 μm. (F) *In situ* physiological assessment revealed functional improvement 4 months after transplantation of expanded teratoma-derived skeletal myogenic progenitors (n = 5–9 biological replicates). ^∗^p < 0.05, ^∗∗^p < 0.01 versus PBS (vehicle). ESCs, embryonic stem cells; α7, α7-integrin; V, VCAM-1; CSA, cross-sectional area. See also Figures S2–S4.

To assess the *in vivo* regenerative potential of expanded teratoma-derived α7^+^ VCAM^+^ cells, we performed intra-muscular transplantations (Figure 3A). We transplanted the freshly sorted or the expanded teratoma-derived skeletal myogenic progenitors into the TA muscles of NSG-mdx^4Cv^ mice (Arpke et al., 2013). These animals are immunocompromised (from the NSG background) and their muscles lack dystrophin (from the mdx^4Cv^ background), thereby allowing allogeneic cell transplantations and identification of donor-derived fibers (DYSTROPHIN^+^). Also, the TA muscles were irradiated and cardiotoxin-injured to create a permissive environment for cell transplantation (Chan et al., 2018). We transplanted into each TA 40,000 cells, a number we have previously determined to be functionally equivalent to the amount of endogenous PAX7^+^ satellite cells in a single TA muscle (Brack et al., 2005; Chan et al., 2018), and evaluated fiber engraftment after 4 months (Figures 3B and 3C). As a direct comparison, we also transplanted freshly isolated and expanded (day 37, or passage 8) endogenous satellite cells harvested from adult muscles (Figures 3B, 3C, and S2A). Freshly sorted teratoma-derived α7^+^ VCAM^+^ cells displayed robust fiber engraftment, regenerating ∼80% of the TA muscle. This level of engraftment is almost equivalent to endogenous satellite cells freshly isolated from adult muscles. Intriguingly, expanded teratoma-derived skeletal myogenic progenitors also produced a significant number of DYSTROPHIN^+^ fibers, reconstituting >40% of the total muscle volume. This is particularly exciting, as endogenous satellite cells rapidly lost their engraftment capability (to ∼5%) once they were in culture (Figures 3B and 3C and Montarras et al., 2005; Sacco et al., 2008).

The remarkable engraftment potential of teratoma-derived skeletal myogenic progenitors that have been cultured for 37 days (passage 8) prompted us to determine whether these cells remain engraftable if they were expanded to a longer time point. To address this, we cultured teratoma-derived skeletal myogenic progenitors in a dish for 79 days to passage 18. These passage 18 cells expanded readily, were mainly α7^+^ VCAM^+^, expressed PAX7, and were capable of differentiating into multinucleated myotubes (Figures S3A–S3D). Remarkably, passage 18 cells remained highly regenerative *in vivo*, with an engraftment level similar to passage 8 cells (Figures S3E and S3F).

The above results were obtained using E14 ESCs. To evaluate whether the expandability of teratoma-derived skeletal myogenic progenitors is also applicable to other PSC lines, we tested C57BL/6N-PRX-B6N #1 ESCs and Pax7-ZsGreen iPSCs (Chan et al., 2018). We found that teratoma-derived α7^+^ VCAM^+^ cells obtained from these PSC lines were expandable (at least up to passage 8) and remained highly skeletal myogenic in forming myotubes *in vitro* and in regenerating new muscle fibers after transplantation (Figures S4A–S4H).

### Newly formed fibers in muscles transplanted with expanded teratoma-derived skeletal myogenic progenitors are functional

Adult skeletal muscles consist of multiple fiber types, including slow-twitch (MHC-I) and fast-twitch (MHC-IIa and MHC-IIb) isoforms. To determine the maturity of the regenerated muscles, we evaluated their fiber type composition. Immunostaining of the newly formed muscles revealed that all of these adult myosin isoforms were present at varying degrees in DYSTROPHIN^+^ fibers derived from both freshly sorted and expanded cells (Figures 3D and S4I). Measurement of individual fiber cross-sectional area also showed similar fiber size distributions between the two cell groups (Figure S4J).

Innervation of newly formed fibers is essential to their maturation and longevity. We undertook staining with α-bungarotoxin, which binds to the nicotinic acetylcholine receptors in the post-synaptic membrane of the neuromuscular junction. We observed a close proximity of α-bungarotoxin immunostaining to DYSTROPHIN^+^ fibers derived from both freshly sorted and expanded cells, suggesting a potential presence of neuromuscular junctions in these newly formed fibers (Figure 3E).

Next, we evaluated whether the expanded cells were capable of endowing functional improvement to the transplanted muscles. Four months post-transplant, we performed physiological assessment to the transplanted TA muscles *in situ*. Comparing with the PBS-injected control muscles, we observed a significant improvement in maximal tetanic force, specific force, and fatigue time in muscles transplanted with expanded teratoma-derived skeletal myogenic progenitors (Figure 3F). These results indicated that the regenerated muscles were functional and capable of force generation.

### Expanded teratoma-derived skeletal myogenic progenitors repopulate the muscle stem cell compartment upon transplantation

**Figure 4:**
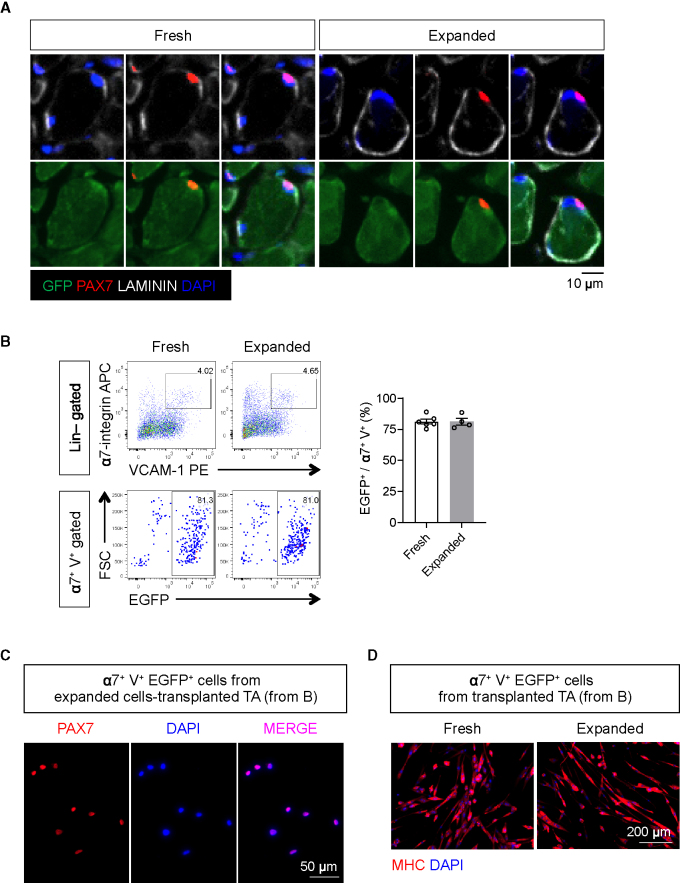
Expanded teratoma-derived skeletal myogenic progenitors reconstitute the muscle stem cell pool after transplantation (A) Immunostaining showing the presence of donor-derived EGFP^+^ PAX7^+^ putative muscle stem cells under the basal lamina in transplanted muscles (representative images from six biological replicates). Scale bar represents 10 μm. (B) FACS analysis (left) and quantification (right; n = 4–6 biological replicates) of transplanted muscles revealed that the majority of Lin^−^ (CD31^−^ CD45^−^) α7^+^ V^+^ muscle stem cells are also EGFP^+^, i.e., donor derived. Data are shown as the mean ± SEM. (C) Reisolated α7^+^ V^+^ EGFP^+^ cells (from [B]) from expanded cell-transplanted TA muscles expressed PAX7 (representative images from three biological replicates). Scale bar represents 50 μm. (D) Reisolated α7^+^ V^+^ EGFP^+^ cells (from [B]) differentiated into multinucleated MHC^+^ myotubes in cultures (representative images from four to six biological replicates). Scale bar represents 200 μm. α7, α7-integrin; V, VCAM-1.

The long-term maintenance of the regenerated muscles is ultimately determined by the ability of the transplanted cells to reconstitute the muscle stem cell pool. We first performed immunostaining on sections of the transplanted muscles with antibodies against EGFP (donor cells), PAX7 (satellite cells), and laminin (sarcolemma). We readily observed EGFP^+^ PAX7^+^ cells under the muscle basal lamina (laminin^+^) from both freshly sorted and expanded cell transplantations (Figure 4A). This indicated that the transplanted cells adopted a satellite cell fate and repopulated the muscle stem cell niche.

We wished to further quantify the extent to which teratoma-derived skeletal myogenic progenitors reconstituted the muscle stem cell compartment. We evaluated the mononuclear fraction of the transplanted muscles by FACS for signs of muscle stem cell contribution. Remarkably, the cultured cells gave rise to as many α7^+^ VCAM^+^ mononuclear cells as the freshly isolated teratoma cells (Figure 4B). These reisolated EGFP^+^ α7^+^ VCAM^+^ cells expressed PAX7 (Figure 4C) and were capable of differentiating into MHC^+^ multinucleated myotubes upon subsequent culture (Figure 4D). Altogether, our results demonstrate that teratoma-derived skeletal myogenic progenitors have an unprecedented level of *in vitro* expansion potential while maintaining a very high potency for muscle regeneration after transplantation.

### Several factors may contribute to the engraftability of expanded teratoma-derived skeletal myogenic progenitors

As illustrated above, expanded teratoma-derived skeletal myogenic progenitors had an engraftment capacity superior to that of expanded adult satellite cells cultured in the same conditions (Figures 3B and 3C). The difference in engraftability might be due to relative PAX7 expression, as *in vitro* expansion over eight passages reduced PAX7 levels in adult satellite cells but not in teratoma-derived skeletal myogenic progenitors (Figures 2A and S2B). Cell senescence might also be a contribution factor, as p21^Waf1/Cip1^ started to emerge in the expanded but not in the freshly isolated teratoma-derived α7^+^ VCAM^+^ cells (Figure S5A). On the other hand, both freshly isolated and expanded teratoma-derived skeletal myogenic progenitors had similar profiles in myotube formation potential (Figure 2C) and cell-cycle stages (Figure S5B).

**Figure 5:**
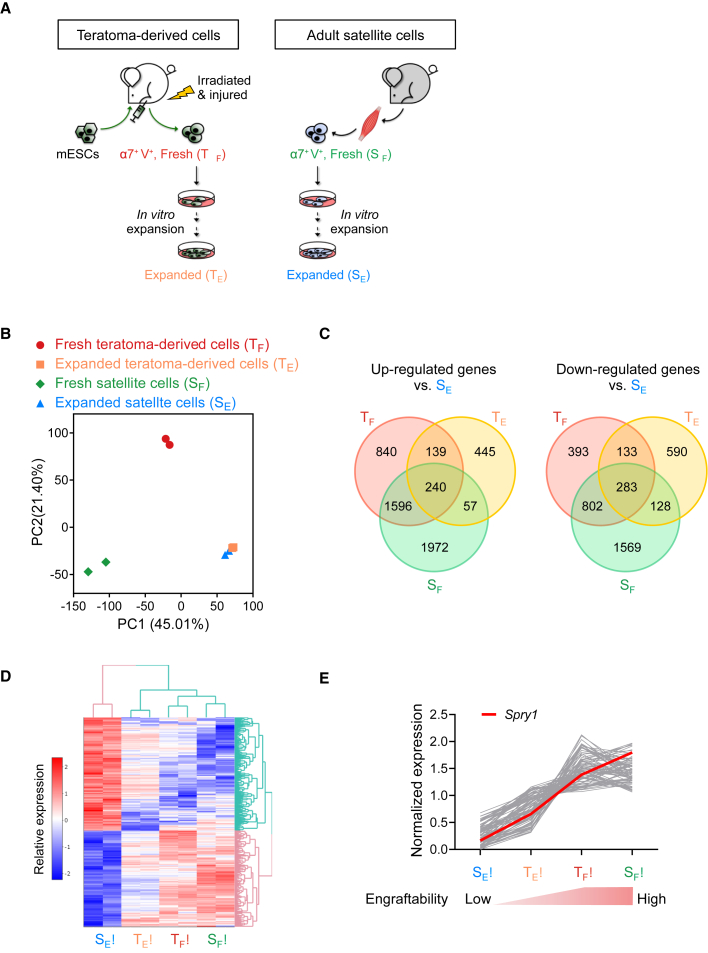
RNA-seq analysis to discover factors that might regulate the engraftability of skeletal myogenic progenitors (A) Schematic of samples used for RNA-seq analysis: freshly isolated (T_F_) and expanded (T_E_) teratoma-derived skeletal myogenic progenitors and freshly isolated (S_F_) and expanded (S_E_) adult satellite cells (n = 2 biological replicates from each group). (B) Principal-component analysis. Note that the transcriptomes of the three engraftable cell populations (T_F_, T_E_, and S_F_) are relatively spread out and the two expanded cell populations (T_E_ and S_E_) are very close to each other. (C) Venn diagrams showing differentially expressed genes (fold change >1.25, adjusted p < 0.05) that are commonly upregulated (left) or downregulated (right) in the three engraftable cell populations (T_F_, T_E_, and S_F_) versus the non-engraftable cells (S_E_). (D) Heatmap showing the 240 upregulated and 283 downregulated genes from (C). (E) Expression of upregulated genes with a Pearson correlation coefficient of >0.9 (gray lines) in the four cell populations. Expression of *Spry1* is shown in red. See text for details. See also Figure S5 and Tables 1 and S2.

To gain further insights into why teratoma-derived skeletal myogenic progenitors have such a high expandability and functionality, we performed an RNA-sequencing (RNA-seq) experiment using four groups of skeletal myogenic cells: fresh teratoma-derived (T_F_), expanded (day 37/passage 8) teratoma-derived (T_E_), fresh satellite (S_F_) (all three engraftable), and expanded (day 37/passage 8) satellite cells (S_E_) (much less engraftable) (Figure 5A). From principal-component analysis, it is intriguing to see that the three engraftable populations are relatively spread out and that the two expanded cell populations are very close to each other, even though one is engraftable and the other is not (Figure 5B). Comparisons between the three engraftable populations (T_F_, T_E_, and S_F_) and the non-engraftable population (S_E_) revealed 240 upregulated genes and 283 downregulated genes (Figures 5C and 5D; Table S1).

We reasoned that factors that regulate engraftability may have their expression directly correlated to engraftment potential, that is, samples with the best engraftment would contain the highest level of engraftment-promoting genes and vice versa. We therefore calculated the Pearson correlation coefficient between engraftment and gene expression and looked for genes with a positive correlation (r > 0.9) (Figure S5C; Table S2). From this analysis, we found *Spry1* (Sprouty1) expression to be highly correlated to engraftment: it is minimally expressed in S_E_ (minimal engraftment), moderately expressed in T_E_ (modest engraftment), and highly expressed in S_F_ and T_F_ (both have the best engraftment) (Figure 5E). Importantly, Sprouty1 has been reported as a key regulator of satellite cell quiescence and self-renewal (Shea et al., 2010). Also, satellite cells that lack Sprouty1 were previously shown to undergo apoptosis during muscle regeneration (Shea et al., 2010), which may explain why cultured satellite cells that minimally express Sprouty1 do not engraft reliably (this study and Sacco et al., 2008). Altogether, Sprouty1, Pax7, and cell senescence might contribute to the engraftability of expanded teratoma-derived skeletal myogenic progenitors.

## Discussion

Differentiation of PSCs into skeletal myogenic cells that can reliably engraft has been difficult. We have previously shown that skeletal myogenic progenitors obtained from PSC-derived teratomas have exceptional *in vivo* regenerative potency in forming new fibers and repopulating the muscle stem cell pool (Chan et al., 2018). Here, we have further extended these findings in showing that teratoma-derived skeletal myogenic progenitors are capable of tremendous *in vitro* expansion without losing their remarkable regenerative power after transplantation.

We have provided multiple lines of *in vitro* and *in vivo* evidence in showing that expanded teratoma-derived skeletal myogenic progenitors have muscle stem cell characteristics. The expanded cells were PAX7^+^, expressed the muscle stem cell surface markers α7 and VCAM, and were capable of forming MHC^+^ colonies with multinucleated myotubes from single cells (i.e., clonal). Most importantly, the expanded cells remained highly engraftable. When culture-expanded cells were transplanted, they differentiated into new force-generating fibers with adult myosins, and developed into PAX7^+^ muscle stem cells residing under the basal lamina. These results suggest that the expanded teratoma-derived skeletal myogenic progenitors retain a muscle stem cell identity.

It should be noted that although the expanded teratoma-derived skeletal myogenic progenitors remained highly regenerative, their fiber engraftment potency was reduced somewhat compared with the freshly sorted teratoma cells. This modest reduction in engraftability could not be adequately explained by most *in vitro* assays, as cells from both groups were comparably potent in expressing the pro-myogenic factors PAX7 and MYOD1 and in differentiating into multinucleated MHC^+^ myotubes. In fact, these *in vitro* features are common to skeletal myogenic populations obtained via various PSC differentiation methods, even though their *in vivo* regenerative potentials differ substantially (Chal et al., 2015; Charville et al., 2015; Gilbert et al., 2010; Hicks et al., 2018; Montarras et al., 2005; Parker et al., 2012; Quarta et al., 2016; Shelton et al., 2014). So far, the field lacks an *in vitro* assay that accurately predicts the *in vivo* engraftability of a putative skeletal myogenic population derived from PSCs. Among the *in vitro* experiments we have performed, we found in the clonal assay that the freshly sorted and the expanded teratoma-derived cells behaved somewhat differently. In the clonal assay, single cells were individually sorted and seeded by FACS, and their ability to survive, self-renew, proliferate, and differentiate into MHC^+^ colonies was collectively evaluated after 8 days in culture. We found that although single cells from both groups had similar cloning efficiencies, colonies developed from the freshly sorted cells were generally larger in size. We reasoned that the proficiency in fiber engraftment from the transplanted cells is governed not only by their potential to express the relevant myogenic factors and undergo proper differentiation but also by their ability to survive in the transplanted muscle milieu. In this regard, the harsh environment in the irradiated and cardiotoxin-damaged muscles is no more unforgiving to the transplanted cells than the stress induced by FACS isolation and the foreign culture conditions is to the single cells. Therefore, the *in vitro* survival/self-renewal capacity of a testing cell population as revealed in the clonal assay might correlate with its *in vivo* engraftment efficiency (Stuelsatz et al., 2015). We reason that clonal analysis might be an effective predictor of engraftability after transplantation.

Large-scale expansion of skeletal myogenic progenitors with high regenerative potential is important for cell therapy applications (Blau and Daley, 2019). Recent clinical trials on cell therapy for treating muscular disorders tested tens to hundreds of millions of cells (Gussoni et al., 1992; Karpati et al., 1993; Mendell et al., 1995; Périé et al., 2014; Skuk et al., 2006, 2007). For example, in a 2014 oculopharyngeal muscular dystrophy trial, an average of 178 million myogenic cells were focally injected to achieve improvement in swallowing (Périé et al., 2014). However, *in vitro* expansion of muscle stem cells is difficult because of the dramatic loss of regenerative capacity after *in vitro* culture (Gussoni et al., 1992; Mendell et al., 1995; Montarras et al., 2005; Sacco et al., 2008). Published methods to optimize the culture conditions to enhance engraftability are so far limited to short-term expansion, and the regenerative potency of the expanded cells thus derived is relatively modest (Charville et al., 2015; Cosgrove et al., 2014; Parker et al., 2012; Quarta et al., 2016). Our study shows that skeletal myogenic progenitors obtained from PSC-derived teratomas can overcome this problem. Over a 37-day culture, teratoma-derived skeletal myogenic progenitors maintained a steady growth and expanded over 1 billion-fold, and remarkably, the expanded cells still reliably engrafted. It is conceivable that these cells could grow even longer in culture. Transplantation of 40,000 expanded cells regenerated more than 40% of the recipient muscle. This level of engraftment from muscle cells in culture is unprecedented—a 3-day *in vitro* culture of endogenous satellite cells dramatically diminished their engraftability by >10-fold, resulting in a modest 300 fibers even though 100,000 cells were transplanted (Montarras et al., 2005). Furthermore, our RNA-seq revealed factors such as Sprouty1, which may regulate the engraftability of expanded teratoma-derived skeletal myogenic progenitors. These factors might be potential candidates to promote *in vitro* expansion of endogenous muscle stem cells with preserved engraftability. We are currently investigating whether the teratoma method to produce expandable and engraftable skeletal myogenic cells is also applicable to human PSCs.

In the current study, we have demonstrated the functional expansion of an engraftable skeletal myogenic population. The possibility of culturing muscle stem cells over a prolonged period of time enables extensive genetic manipulation, which in turn is fundamental to combining gene and cell therapies to treat muscle disorders (Amoasii et al., 2018; Konieczny et al., 2013). Further investigations to address the molecular mechanisms underlying the high regenerative capacity and *in vitro* expandability of teratoma-derived skeletal myogenic progenitors would provide valuable insights to functionally expand endogenous muscle stem cells *in vitro*.

## Experimental procedures

Details can be found in the supplemental experimental procedures.

### Animals

All animal procedures were performed according to the University of Minnesota Institutional Animal Care and Use Committee guidelines and approved protocols.

### Teratoma formation, cell transplantation, and engraftment evaluation

Recipient NSG-mdx^4Cv^ mice with their hindlimbs irradiated and TA muscles cardiotoxin-injured were used for both teratoma formation and skeletal myogenic cell transplantations. For teratoma formation, 250,000 ESCs were injected into the TA muscle. Teratomas were harvested 4 weeks later. For cell transplantations, 40,000 freshly isolated or cultured/expanded cells derived from teratomas or adult muscles were transplanted into the TA muscle. Transplanted muscles were harvested and analyzed 4 months later.

### RNA-seq analysis

Paired-end 150-bp sequencing libraries were created using the SMARTer Stranded Total RNA-Seq Kit v.2-Pico Input Mammalian Kit (Clontech, Mountain View, CA). Differentially expressed genes were identified with fold change >1.25 and adjusted p < 0.05.

### Data and code availability

The accession number for the data reported in this paper is GEO: GSE182508.

## Author contributions

Conceptualization: S.S.K.C.; methodology: N.X., M.K., R.C.R.P., and S.S.K.C.; investigation: N.X., S.N.C., K.A., C.S., and L.N.P.; formal analysis: N.X.; visualization: N.X. and S.S.K.C.; writing: N.X. and S.S.K.C.; funding acquisition: M.K., R.C.R.P., and S.S.K.C.; supervision: S.S.K.C.

## Conflicts of interest

The authors declare no competing interests.

## Supplemental information

Document S1. supplemental experimental procedures and Figures S1–S5

Table S1. Common differentially expressed genes in engraftable cells versus non-engraftable cells, related to Figure 5

Table S2. Pearson correlation analysis of differentially expressed genes, related to Figure 5

Document S2. Article plus supplemental information

## References

[bib1] Al Tanoury Z., Rao J., Tassy O., Gobert B., Gapon S., Garnier J.M., Wagner E., Hick A., Hall A., Gussoni E. (2020). Differentiation of the human PAX7-positive myogenic precursors/satellite cell lineage in vitro. Development.

[bib2] Amoasii L., Hildyard J.C.W., Li H., Sanchez-Ortiz E., Mireault A., Caballero D., Harron R., Stathopoulou T.R., Massey C., Shelton J.M. (2018). Gene editing restores dystrophin expression in a canine model of Duchenne muscular dystrophy. Science.

[bib3] Arpke R.W., Darabi R., Mader T.L., Zhang Y., Toyama A., Lonetree C.L., Nash N., Lowe D.A., Perlingeiro R.C., Kyba M. (2013). A new immuno-, dystrophin-deficient model, the NSG-mdx(4Cv) mouse, provides evidence for functional improvement following allogeneic satellite cell transplantation. Stem Cells.

[bib4] Blau H.M., Daley G.Q. (2019). Stem cells in the treatment of disease. N. Engl. J. Med..

[bib5] Brack A.S., Bildsoe H., Hughes S.M. (2005). Evidence that satellite cell decrement contributes to preferential decline in nuclear number from large fibres during murine age-related muscle atrophy. J. Cell Sci..

[bib6] Brack A.S., Conboy I.M., Conboy M.J., Shen J., Rando T.A. (2008). A temporal switch from notch to Wnt signaling in muscle stem cells is necessary for normal adult myogenesis. Cell Stem Cell.

[bib7] Chal J., Oginuma M., Al Tanoury Z., Gobert B., Sumara O., Hick A., Bousson F., Zidouni Y., Mursch C., Moncuquet P. (2015). Differentiation of pluripotent stem cells to muscle fiber to model Duchenne muscular dystrophy. Nat. Biotechnol..

[bib8] Chal J., Pourquié O. (2017). Making muscle: skeletal myogenesis in vivo and in vitro. Development.

[bib9] Chan S.S., Arpke R.W., Filareto A., Xie N., Pappas M.P., Penaloza J.S., Perlingeiro R.C.R., Kyba M. (2018). Skeletal muscle stem cells from PSC-derived teratomas have functional regenerative capacity. Cell Stem Cell.

[bib10] Chan S.S., Shi X., Toyama A., Arpke R.W., Dandapat A., Iacovino M., Kang J., Le G., Hagen H.R., Garry D.J. (2013). Mesp1 patterns mesoderm into cardiac, hematopoietic, or skeletal myogenic progenitors in a context-dependent manner. Cell Stem Cell.

[bib11] Charville G.W., Cheung T.H., Yoo B., Santos P.J., Lee G.K., Shrager J.B., Rando T.A. (2015). In vitro expansion and in vivo self-renewal of human muscle stem cells. Stem Cell Reports.

[bib12] Collins C.A., Olsen I., Zammit P.S., Heslop L., Petrie A., Partridge T.A., Morgan J.E. (2005). Stem cell function, self-renewal, and behavioral heterogeneity of cells from the adult muscle satellite cell niche. Cell.

[bib13] Cooper R.N., Tajbakhsh S., Mouly V., Cossu G., Buckingham M., Butler-Browne G.S. (1999). In vivo satellite cell activation via Myf5 and MyoD in regenerating mouse skeletal muscle. J. Cell Sci..

[bib14] Cosgrove B.D., Gilbert P.M., Porpiglia E., Mourkioti F., Lee S.P., Corbel S.Y., Llewellyn M.E., Delp S.L., Blau H.M. (2014). Rejuvenation of the muscle stem cell population restores strength to injured aged muscles. Nat. Med..

[bib15] Darabi R., Arpke R.W., Irion S., Dimos J.T., Grskovic M., Kyba M., Perlingeiro R.C. (2012). Human ES- and iPS-derived myogenic progenitors restore DYSTROPHIN and improve contractility upon transplantation in dystrophic mice. Cell Stem Cell.

[bib16] Filareto A., Parker S., Darabi R., Borges L., Iacovino M., Schaaf T., Mayerhofer T., Chamberlain J.S., Ervasti J.M., McIvor R.S. (2013). An in vitro gene therapy approach to treat muscular dystrophy using inducible pluripotent stem cells. Nat. Commun..

[bib17] Gilbert P.M., Havenstrite K.L., Magnusson K.E., Sacco A., Leonardi N.A., Kraft P., Nguyen N.K., Thrun S., Lutolf M.P., Blau H.M. (2010). Substrate elasticity regulates skeletal muscle stem cell self-renewal in culture. Science.

[bib18] Giordani L., He G.J., Negroni E., Sakai H., Law J.Y.C., Siu M.M., Wan R., Corneau A., Tajbakhsh S., Cheung T.H. (2019). High-dimensional single-cell cartography reveals novel skeletal muscle-resident cell populations. Mol. Cell.

[bib19] Günther S., Kim J., Kostin S., Lepper C., Fan C.M., Braun T. (2013). Myf5-positive satellite cells contribute to Pax7-dependent long-term maintenance of adult muscle stem cells. Cell Stem Cell.

[bib20] Gussoni E., Pavlath G.K., Lanctot A.M., Sharma K.R., Miller R.G., Steinman L., Blau H.M. (1992). Normal dystrophin transcripts detected in Duchenne muscular dystrophy patients after myoblast transplantation. Nature.

[bib21] Hicks M.R., Hiserodt J., Paras K., Fujiwara W., Eskin A., Jan M., Xi H., Young C.S., Evseenko D., Nelson S.F. (2018). ERBB3 and NGFR mark a distinct skeletal muscle progenitor cell in human development and hPSCs. Nat. Cell Biol..

[bib22] Ippolito J., Arpke R.W., Haider K.T., Zhang J., Kyba M. (2012). Satellite cell heterogeneity revealed by G-Tool, an open algorithm to quantify myogenesis through colony-forming assays. Skelet. Muscle.

[bib23] Jiwlawat N., Lynch E., Jeffrey J., Van Dyke J.M., Suzuki M. (2018). Current progress and challenges for skeletal muscle differentiation from human pluripotent stem cells using transgene-free approaches. Stem Cells Int..

[bib24] Karpati G., Ajdukovic D., Arnold D., Gledhill R.B., Guttmann R., Holland P., Koch P.A., Shoubridge E., Spence D., Vanasse M. (1993). Myoblast transfer in Duchenne muscular dystrophy. Ann. Neurol..

[bib25] Konieczny P., Swiderski K., Chamberlain J.S. (2013). Gene and cell-mediated therapies for muscular dystrophy. Muscle Nerve.

[bib26] Mauro A. (1961). Satellite cell of skeletal muscle fibers. J. Biophys. Biochem. Cytol..

[bib27] Mendell J.R., Kissel J.T., Amato A.A., King W., Signore L., Prior T.W., Sahenk Z., Benson S., McAndrew P.E., Rice R. (1995). Myoblast transfer in the treatment of Duchenne's muscular dystrophy. N. Engl. J. Med..

[bib28] Montarras D., Morgan J., Collins C., Relaix F., Zaffran S., Cumano A., Partridge T., Buckingham M. (2005). Direct isolation of satellite cells for skeletal muscle regeneration. Science.

[bib29] Parker M.H., Loretz C., Tyler A.E., Duddy W.J., Hall J.K., Olwin B.B., Bernstein I.D., Storb R., Tapscott S.J. (2012). Activation of Notch signaling during in vitro expansion maintains donor muscle cell engraftment. Stem Cells.

[bib30] Périé S., Trollet C., Mouly V., Vanneaux V., Mamchaoui K., Bouazza B., Marolleau J.P., Laforêt P., Chapon F., Eymard B. (2014). Autologous myoblast transplantation for oculopharyngeal muscular dystrophy: a phase I/IIa clinical study. Mol. Ther..

[bib31] Quarta M., Brett J.O., DiMarco R., De Morree A., Boutet S.C., Chacon R., Gibbons M.C., Garcia V.A., Su J., Shrager J.B. (2016). An artificial niche preserves the quiescence of muscle stem cells and enhances their therapeutic efficacy. Nat. Biotechnol..

[bib32] Roth S.M., Martel G.F., Ivey F.M., Lemmer J.T., Metter E.J., Hurley B.F., Rogers M.A. (2000). Skeletal muscle satellite cell populations in healthy young and older men and women. Anat. Rec..

[bib33] Sacco A., Doyonnas R., Kraft P., Vitorovic S., Blau H.M. (2008). Self-renewal and expansion of single transplanted muscle stem cells. Nature.

[bib34] Schultz E., Gibson M.C., Champion T. (1978). Satellite cells are mitotically quiescent in mature mouse muscle: an EM and radioautographic study. J. Exp. Zool..

[bib35] Seale P., Asakura A., Rudnicki M.A. (2001). The potential of muscle stem cells. Dev. Cell.

[bib36] Seale P., Bjork B., Yang W., Kajimura S., Chin S., Kuang S., Scimè A., Devarakonda S., Conroe H.M., Erdjument-Bromage H. (2008). PRDM16 controls a brown fat/skeletal muscle switch. Nature.

[bib37] Shea K.L., Xiang W., LaPorta V.S., Licht J.D., Keller C., Basson M.A., Brack A.S. (2010). Sprouty1 regulates reversible quiescence of a self-renewing adult muscle stem cell pool during regeneration. Cell Stem Cell.

[bib38] Shelton M., Metz J., Liu J., Carpenedo R.L., Demers S.P., Stanford W.L., Skerjanc I.S. (2014). Derivation and expansion of PAX7-positive muscle progenitors from human and mouse embryonic stem cells. Stem Cell Reports.

[bib39] Skuk D., Goulet M., Roy B., Chapdelaine P., Bouchard J.P., Roy R., Dugré F.J., Sylvain M., Lachance J.G., Deschênes L. (2006). Dystrophin expression in muscles of duchenne muscular dystrophy patients after high-density injections of normal myogenic cells. J. Neuropathol. Exp. Neurol..

[bib40] Skuk D., Goulet M., Roy B., Piette V., Côté C.H., Chapdelaine P., Hogrel J.Y., Paradis M., Bouchard J.P., Sylvain M. (2007). First test of a “high-density injection” protocol for myogenic cell transplantation throughout large volumes of muscles in a Duchenne muscular dystrophy patient: eighteen months follow-up. Neuromuscul. Disord..

[bib41] Snow M.H. (1977). Myogenic cell formation in regenerating rat skeletal muscle injured by mincing. II. An autoradiographic study. Anat. Rec..

[bib42] Stuelsatz P., Shearer A., Li Y., Muir L.A., Ieronimakis N., Shen Q.W., Kirillova I., Yablonka-Reuveni Z. (2015). Extraocular muscle satellite cells are high performance myo-engines retaining efficient regenerative capacity in dystrophin deficiency. Dev. Biol..

[bib43] Tierney M.T., Gromova A., Sesillo F.B., Sala D., Spenlé C., Orend G., Sacco A. (2016). Autonomous extracellular matrix remodeling controls a progressive adaptation in muscle stem cell regenerative capacity during development. Cell Rep..

[bib44] von Maltzahn J., Jones A.E., Parks R.J., Rudnicki M.A. (2013). Pax7 is critical for the normal function of satellite cells in adult skeletal muscle. Proc. Natl. Acad. Sci. U S A.

[bib45] Zammit P.S., Golding J.P., Nagata Y., Hudon V., Partridge T.A., Beauchamp J.R. (2004). Muscle satellite cells adopt divergent fates: a mechanism for self-renewal?. J. Cell Biol..

