# Supplementary material for: In vitro expanded skeletal myogenic progenitors from pluripotent stem cell-derived teratomas have high engraftment capacity
Source: Stem Cell Reports. 2021 Nov 18;16(12):2900–12. doi: 10.1016/j.stemcr.2021.10.014 (PMC8693664; doi:10.1016/j.stemcr.2021.10.014)
Supplement: Document S1. supplemental experimental procedures and Figures S1–S5 [file mmc1.pdf]

Stem Cell Reports, Volume *16*

Supplemental Information

In vitro expanded skeletal myogenic progenitors from pluripotent stem cell-derived teratomas have high engraftment capacity

Ning Xie, Sabrina N. Chu, Karim Azzag, Cassandra B. Schultz, Lindsay N. Peifer, Michael Kyba, Rita C.R. Perlingeiro, and Sunny S.K. Chan

Supplemental Figures

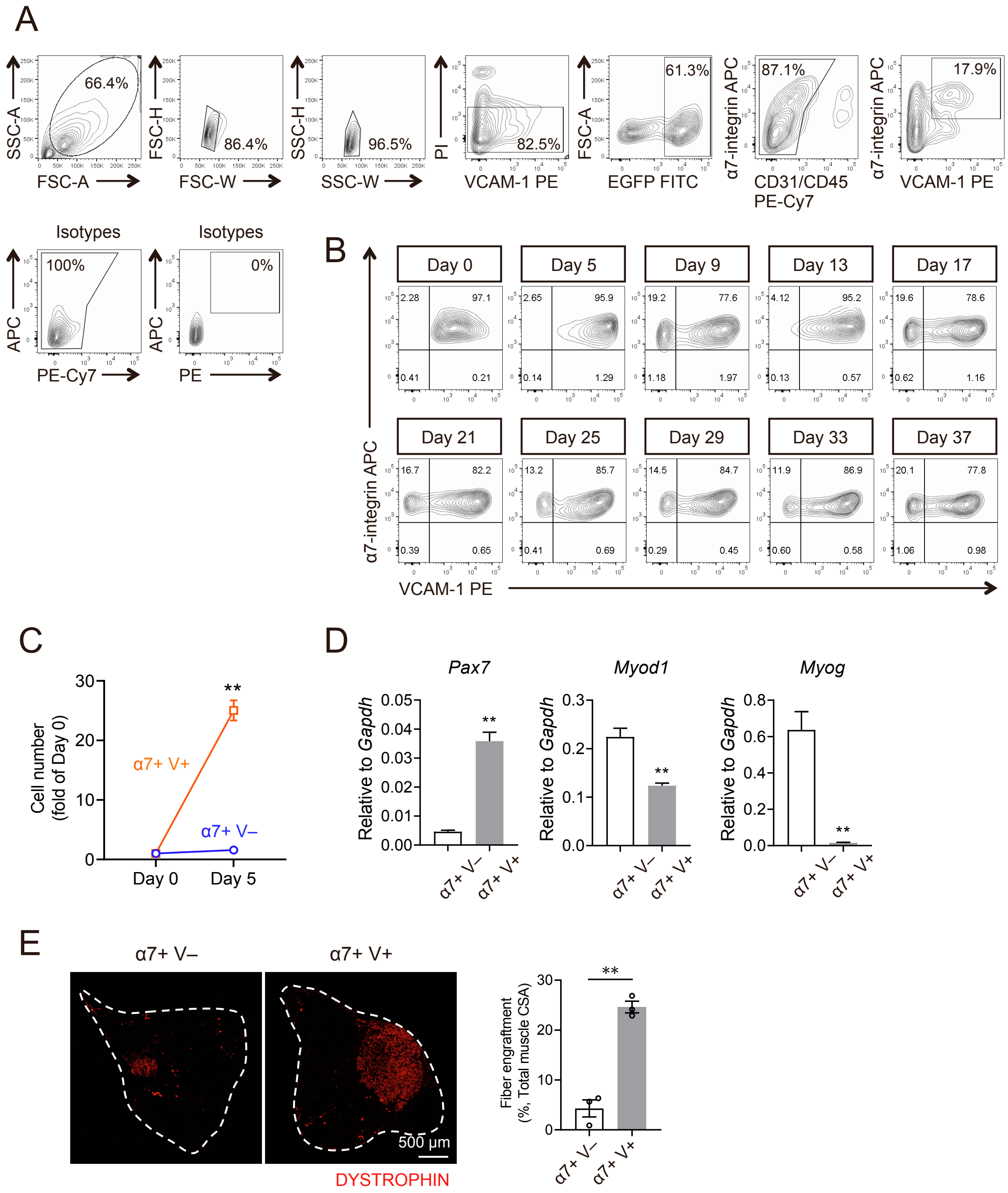

Figure S1, related to Figure 1. FACS profiling and comparison between α7+ VCAM+ and α7+ VCAM– populations in expanded teratoma-derived skeletal myogenic progenitors

(A) Representative FACS gatings for identifying skeletal myogenic progenitors in mouse ESC-derived teratomas.

(B) FACS profiling of teratoma-derived skeletal myogenic progenitors in cultures over 37 days. Note that the FACS plots for Day 0 and Day 37 were also used in Figure 1C as "Fresh" and "Expanded" respectively.

(C) α7+ V+ and α7+ V– cells were sorted from expanded (day 37/passage 8) teratoma-derived skeletal myogenic progenitors and were subsequently cultured for another 5 days. α7+ V+ cells grew more readily than α7+ V– cells. Data are shown as mean ± SEM from 3 independent experiments. $^{**}p < 0.01$.

(D) Quantitative RT-PCR analysis for markers of muscle stem cells (*Pax7*), committed myogenic cells (*Myod1*) and differentiated myocytes (*Myog*) in α7+ V+ and α7+ V– cells sorted from expanded (day 37/passage 8) teratoma-derived skeletal myogenic progenitors. Data are shown as mean ±SEM from 3 independent experiments. $^{**}p < 0.01$.

(E) Representative images (left) and quantification (right) of fiber engraftment (DYSTROPHIN+ fibers) of α7+ V+ and α7+ V– cells sorted from expanded (day 37/passage 8) teratoma-derived skeletal myogenic progenitors. Data are shown as mean ± SEM from 3 biological replicates. $^{**}p < 0.01$.

ESC, embryonic stem cells. α7, α7-integrin. V, VCAM-1. CSA, cross-sectional area.

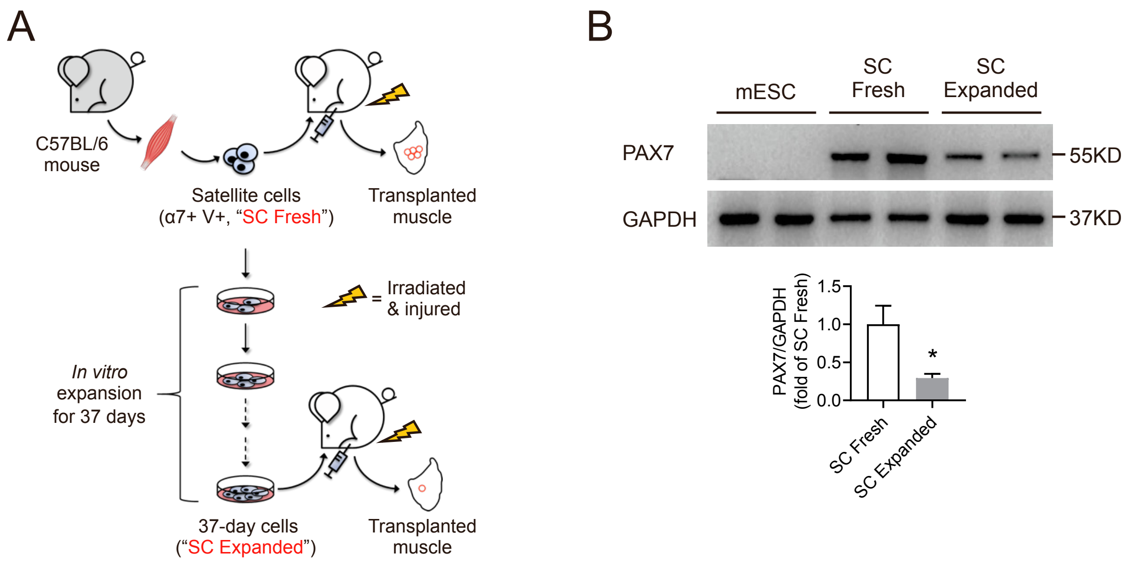

Figure S2, related to Figures 2 and 3. Isolation and characterization of adult satellite cells

(A) Schematic of *in vitro* expansion of endogenous satellite cells isolated from adult muscle tissues.

(B) Immunoblots (top) and quantification (bottom) of PAX7 protein expression in freshly-isolated and expanded adult satellite cells. Mouse ESCs were used as a control. Data are shown as mean ±SEM from 4 independent experiments. *$p < 0.05$.

α7, α7-integrin. V, VCAM-1. mESC, mouse embryonic stem cells. SC, satellite cells.

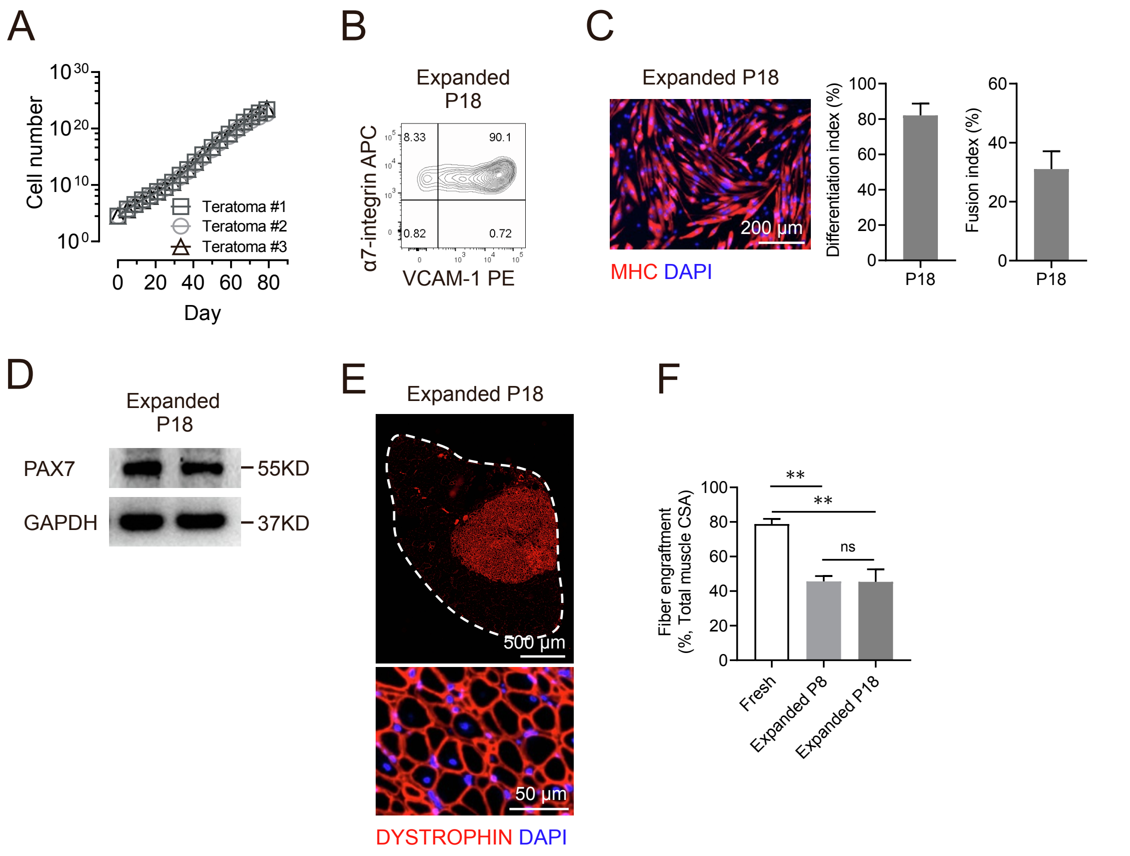

Figure S3, related to Figure 3. Teratoma-derived skeletal myogenic progenitors remain engraftable after 79 days (passage 18) of *in vitro* expansion

(A) *In vitro* cultures of teratoma-derived skeletal myogenic progenitors grew steadily for up to 79 days (passage 18). Data are shown as mean ±SEM from 3 independent experiments.

(B) FACS profile of expanded passage 18 (P18) teratoma-derived skeletal myogenic progenitors.

(C) Immunostaining (left) of MHC in expanded P18 cells cultured in differentiation medium. Scale bar represents 200 μm. Quantification of differentiation (middle) and

fusion in MHC+ myotubes (right) from 3 independent experiments. Data are shown as mean ± SEM.

(D) Immunoblots showing that expanded P18 cells expressed the muscle stem cell transcription factor PAX7 (2 independent samples from each cell group are shown).

(E) Expanded P18 teratoma-derived skeletal myogenic progenitors engrafted and formed DYSTOPHIN+ fibers 4 months post-transplant. The whole TA muscles are outlined (top, scale bar represents 500 μm), and magnified images are shown (bottom, scale bar represents 50 μm).

(F) Quantitation of fiber engraftment (DYSTROPHIN+ fibers) in TA muscles transplanted with freshly-sorted, expanded P8 and expanded P18 skeletal myogenic progenitors derived from teratomas (n=9-12 biological replicates). Data are shown as mean ± SEM. ∗∗$p < 0.01$. ns, not significant. Note that the quantification for "Fresh" and "Expanded P8" were also used in Figure 3C as "Fresh, teratoma-derived" and "Expanded, teratoma-derived", respectively.

MHC, myosin heavy chain. PI, propidium iodide. CSA, cross-sectional area.

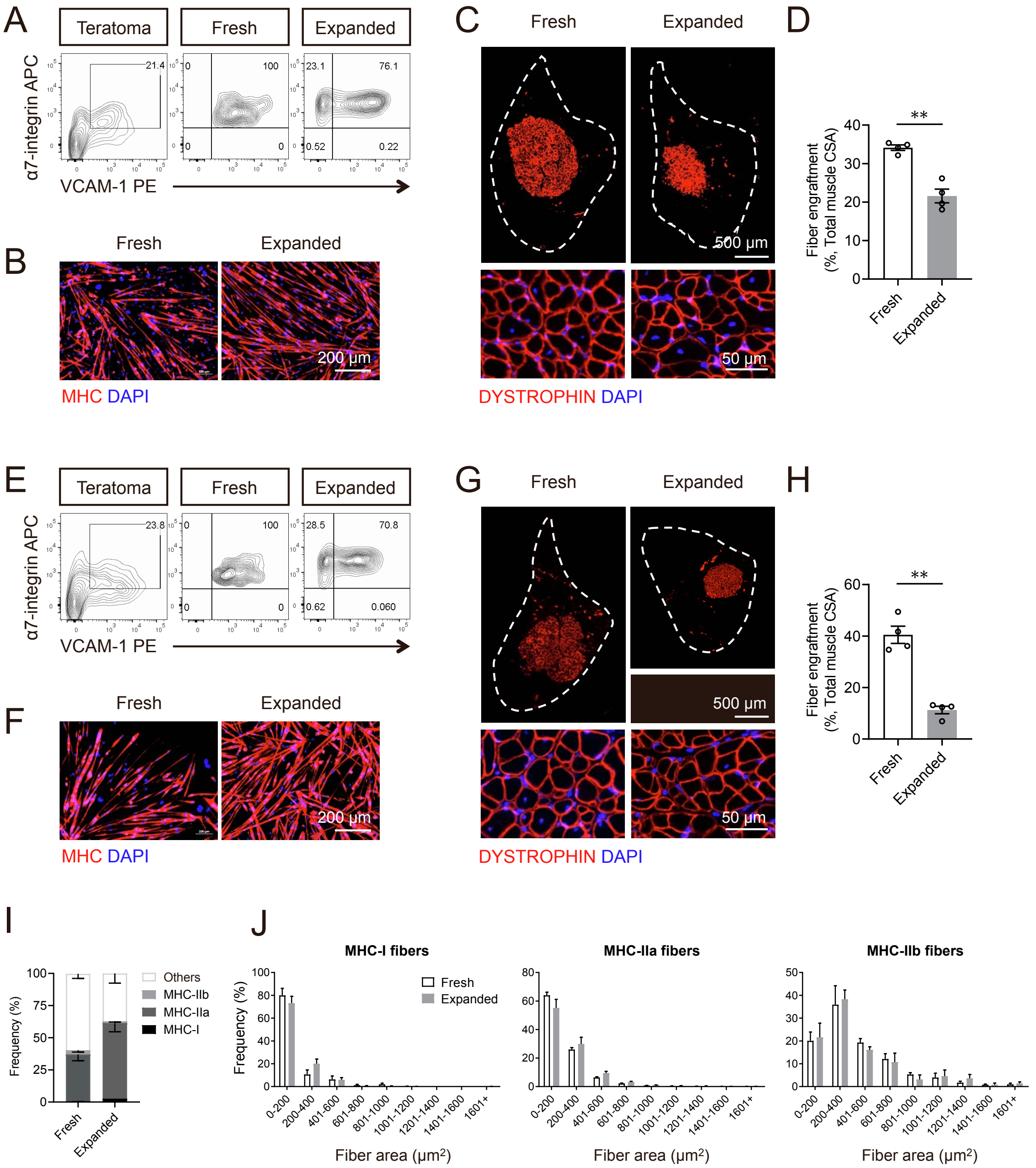

Figure S4, related to Figure 3. Evaluation of the functionality of expanded teratoma-derived skeletal myogenic progenitors generated from multiple PSC lines

C57BL/6N-PRX-B6N #1 ESCs (A-D) and Pax7-ZsGreen iPSCs (E-H) were used to generate teratoma-derived α7+ V+ cells, whose *in vitro* expandability and skeletal myogenic potential were evaluated.

(A, E) FACS profiling of total teratoma cells, freshly-sorted α7+ V+ cells and expanded 37-day (passage 8) cells.

(B, F) Immunostaining of MHC in freshly-sorted and expanded cells cultured in differentiation medium. Scale bar represents 200 μm.

(C, G) Freshly-sorted and expanded teratoma-derived skeletal myogenic progenitors engrafted and formed DYSTOPHIN+ fibers 6 weeks post-transplant. The whole TA muscles are outlined (top, scale bar represents 500 μm), and magnified images are shown (bottom, scale bar represents 50 μm).

(D, H) Quantitation of fiber engraftment (DYSTROPHIN+ fibers) (n=4 biological replicates). Data are shown as mean ± SEM. **p < 0.01.

(I) Quantification of various fiber types of engrafted DYSTROPHIN+ muscle fibers derived from freshly-sorted and expanded teratoma-derived skeletal myogenic progenitors. Data are shown as mean ± SEM from 6 biological replicates.

(J) Area distribution of individual DYSTROPHIN+ fibers expressing MHC-I (left), MHC-IIa (middle) and MHC-IIb (right). Data are shown as mean ± SEM from 6 biological replicates.

ESC, embryonic stem cells. iPSC, induced pluripotent stem cells. α7, α7-integrin. V, VCAM-1. MHC, myosin heavy chain. CSA, cross-sectional area.

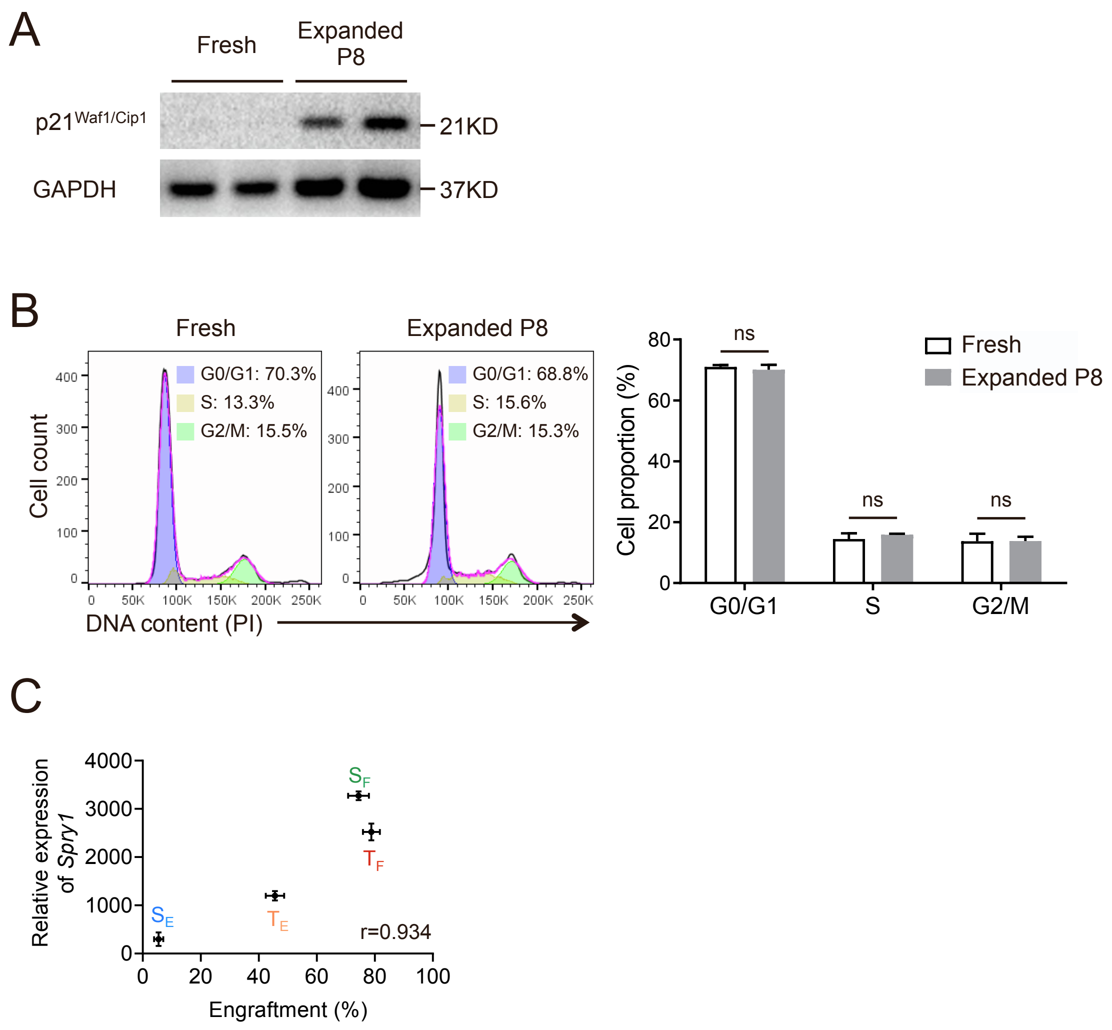

Figure S5, related to Figure 5. Potential factors affecting engraftment

(A) Immunoblots showing p21[Waf1/Cip1] protein expression in expanded P8 cells but not in fresh cells (2 independent samples from each cell group are shown).

(B) FACS profiling (left) and quantification (right) of cell cycle analysis of fresh and expanded P8 cells. Cells were stained with PI and analyzed by FACS. Data are shown as mean ± SEM of 3 independent experiments. ns, not significant.

(C) Pearson correlation coefficient (r) between engraftment (%) and *Spry1* gene expression (MRN-normalized read counts) from S_E, T_E, T_F and S_F samples.

Supplemental Tables

Table S1, related to Figure 5. Common differentially expressed genes in engraftable cells vs. non-engraftable cells

Table S2, related to Figure 5. Pearson correlation analysis of differentially expressed genes

Supplemental Experimental Procedures

Animals

Animal housing, husbandry and experiments were carried out according to protocols (#1903-36840A) approved by the University of Minnesota Institutional Animal Care and Use Committee and under institutional assurances of AAALAC accreditation (#000552, as of Nov 2015), USDA research facility registration (USDA No. 41-R-0005), and PHS Animal Welfare Assurance approval (A3456-01). The NSG-mdx^{4Cv} mice has been described (Arpke et al., 2013), and were generated by crossing NOD.Cg-Prkdc^{scid}Il2rg^{tm1Wjl}/SzJ (NSG) mice and B6Ros.Cg-Dmd$^{mdx\text{-}4Cv}$/J (mdx^{4Cv}) mice and maintained in autoclaved cages. NSG-mdx^{4Cv} mice of both sexes (3-4 months old) were used for experiments. Male C57BL/6J mice (3-4 months old) were obtained from Jackson Laboratory (#000664, Bar Harbor, ME).

ESCs/iPSCs culture

E14-EGFP mouse ESCs (Ismailoglu et al., 2008), C57BL/6N-PRX-B6N #1 mouse ESCs (Jackson Laboratory #012448, via Mouse Genetics Laboratory, University of Minnesota, MN) and Pax7-ZsGreen mouse iPSCs (Chan et al., 2018) were cultured on irradiated mouse embryonic fibroblasts (MEFs) in maintenance medium containing Knock-Out Dulbecco's Minimum Essential Medium (DMEM) (Life Technologies #10829-018, Grand Island, NY), 15% fetal bovine serum (FBS) (Gemini Bio-Products #100-119, West Sacramento, CA), 1% non-essential amino acids (Life Technologies #11140-050), 1% penicillin/streptomycin (P/S) (Life Technologies #15140-122), 2 mM Glutamax (Life Technologies #SCR006), 0.1 mM β-mercaptoethanol (Sigma #M3148, St. Louis, MO)

and 500 U/mL leukemia inhibitory factor (Millipore #ESG1107, Temecula, CA), at 37°C in 5% CO_2. Cells were fed with fresh medium every day and were passaged with 0.25% trypsin-EDTA (Life Technologies #25200-072) every other day. On the day of teratoma formation, ESCs were dissociated with 0.25% trypsin-EDTA and plated on a cell culture flask for 45-60 min to remove MEFs.

Teratoma formation

Mouse ESCs/iPSCs were injected into tibialis anterior (TA) muscles of NSG-mdx^{4Cv} mice to generate teratomas. Two days prior to cell injections, recipients were anesthetized with ketamine (150 mg/kg, i.p., Akorn #NDC:59399-114-10, Lake Forest, IL) and xylazine (10 mg/kg, i.p., Akorn #NDC:59399-111-50), and left hindlimbs were exposed to 1200 cGy X-ray irradiation. One day before cell injections, 15 μL of cardiotoxin (10 μM, Latoxan #L8102, France) was injected into the irradiated muscles to induce muscle injury. The next day, 250,000 ESCs or 1,000,000 iPSCs were resuspended in 10 μL PBS (HyClone #SH30256.01, Logan, UT) and injected into the TA muscles using a Hamilton syringe (Hamilton, Reno, NV).

Teratoma harvest

Four week-old teratomas were dissected and mechanically chopped into small pieces. Chopped tissues were then incubated in primary digestion buffer consisting of DMEM/high glucose (HyClone #SH30243.01), 2 mg/mL Collagenase II (Gibco #17101-015, Gaithersburg, MD) and 1% P/S on a shaker at 250 rpm, 37°C for 30 min. The digested cell supernatant was filtered through 40 μm cell strainers and spun down, then

the filtered cell pellet was washed with rinsing buffer consisting of Ham's/F-10 medium (Caisson Labs #HFL01, Smithfield, UT), 10% horse serum (HyClone #SH30074.03), 1% HEPES buffer solution (Caisson Labs #HOL06) and 1% P/S and kept on ice (first-pass). The undigested chunk tissues were further incubated in primary digestion buffer on a shaker at 250 rpm, 37°C for another 30 min, then filtered through 40 µm cell strainers, spun down and the cell pellet was washed with rinsing buffer (second-pass). The cells from first-pass and second-pass were combined together, resuspended in FACS buffer (PBS with 0.2% FBS) and kept on ice until downstream FACS staining.

Satellite cells isolation

Hindlimb muscles were removed from C57BL/6J mice and then mechanically chopped into small pieces. Chopped tissues were incubated in primary digestion buffer on a shaker at 250 rpm, 37°C for 75 min. Primary digestion was stopped by adding rinsing buffer and tissue spun down at 1500 rpm for 5 min at 4°C. The tissues were further digested in secondary digestion buffer consisting of rinsing buffer supplemented with 0.1 mg/mL Collagenase II and 0.5 mg/mL Dispase (Gibco, Cat#17105-041) on a shaker at 250 rpm, 37°C for 30 min. Digested tissues were vortexed, drawn and released into a 10 mL syringe with a 16-gauge needle four times and then with an 18-gauge needle four times. Dissociated tissues were filtered through 40 µm cell strainers, spun down, then washed with rinsing buffer and resuspended in FACS buffer on ice. Cells were stained and sorted as described below and endogenous satellite cells are defined as CD31– CD45– α7-integrin+ VCAM-1+.

Cell isolation from transplanted TA muscles

Transplanted TA muscles were removed and were processed the same way as satellite cells isolation.

Cell transplantation

NSG-mdx^{4Cv} mice were irradiated and cardiotoxin-injured as mentioned above. For cell transplantations, 40,000 cells (derived from teratomas or isolated from adult muscles) in PBS was injected into TA muscles using a Hamilton syringe. Grafted TA muscles were harvested at 4 months after transplantation and processed for immunostaining or fluorescence activated cell sorting (FACS) analysis.

Fluorescence-activated cell sorting (FACS)

Dissociated cells were filtered through 40 μm cell strainers and incubated with fluorophore-conjugated antibodies on ice for 30 min. Stained cells were washed twice in FACS buffer (PBS with 0.2% FBS) and resuspended in FACS buffer with propidium iodide (PI, 1 μg/mL, Sigma #P4170) for FACS analysis. PI was used as a live/dead cell indicator and only live cells (PI–) were counted. Cells were analyzed and sorted by BD FACSAriaII (BD Biosciences, San Diego, CA) with FACSDiva software (BD Biosciences). Four-way purity precision was used for bulk sorting and single-cell precision was used for sorting single cells into 96-well plate for clonal analysis. Sorted cells were collected into cell culture medium and kept cold until downstream processing. FACS plots were generated using FlowJo (FLOWJO LLC, Ashland, OR). Antibodies used were PE-Cy7 anti-CD31, BD Biosciences #561410; RRID:AB_10612003, San Jose,

CA; PE-Cy7 anti-CD45, BD Biosciences #552848; RRID:AB_394489; APC anti-α7-Integrin, AbLab #67-0010-05, Vancouver, Canada; Biotin anti-VCAM-1, BD Biosciences #553331; RRID:AB_10053328; PE streptavidin, BD Biosciences #554061; all at 0.5 µL per million cells. To analyze cell-cycle progression, cells were harvested and immobilized in 70% ethanol at 4°C for 12 hr, followed by washing with PBS. Then the cells were incubated in PI (50 µg/mL) with RNase (10 µg/mL) for 30 min. 10,000 cells were analyzed for each sample using FACSAriaII.

In vitro cell expansion

To expand the teratoma-derived skeletal myogenic progenitors, FACS-sorted CD31– CD45– α7+ VCAM+ cells were plated on 0.1% gelatin-coated wells and cultured in myogenic expansion medium containing Ham's/F-10, 20% FBS (Sigma #F0926), 10 ng/mL basic FGF (R&D Systems #233-FB/CF, Minneapolis, MN), 1% P/S, 2 mM Glutamax and 0.1 mM β-mercaptoethanol. Cells were passaged every 4-5 days in myogenic expansion medium when they reached 40% to 50% confluency. Cells (freshly-isolated or expanded) were plated in myogenic expansion medium and cultured for 3 days before processing to immunostaining with PAX7 and MYOD1 and imaging. Endogenous satellite cells were cultured and expanded similarly.

In vitro skeletal myogenic differentiation

Skeletal myogenic progenitors were differentiated into myotubes at 70% to 80% confluency. Medium was switched to myogenic differentiation medium containing high-glucose DMEM, 2% horse serum, 1% insulin-transferrin-selenium (Life Technologies

#41400045) and 1% P/S for 3 days. Cells at the end of differentiation were subjected to immunostaining with myosin heavy chain (MHC). Cells were imaged and analyzed. Differentiation index was calculated as the percentage of nuclei within MHC+ cells relative to the total number of nuclei. Fusion index was calculated as the percentage of nuclei within myotubes (elongated MHC+ cells containing at least three nuclei) relative to the total number of nuclei. Approximately 100 myotubes were counted for each replicate.

Clonal analysis

Single cells were seeded via FACS into gelatin-coated 96-well plates (one cell per well) in myogenic clonal medium containing DMEM/F12 (Cellgro #15-090-CV, Manassas, VA), 20% FBS, 10% horse serum, 10 ng/mL basic FGF, 1% P/S, 2 mM Glutamax and 0.5% chick embryo extract (US Biological #C3999, Salem, MA). This medium supports both proliferation and differentiation (Ippolito et al., 2012). Cells were cultured at 37 °C, 5% CO_2 in a humidified tissue culture incubator. Cells were left undisturbed for 8 days, followed by immunostaining with MHC and 4',6-diamidino-2-phenylindole (DAPI) (Life Technologies #D3571). Clone size was determined by counting the number of DAPI-positive nuclei using ImageJ software.

Western blotting

One hundred thousand cells were used for each lane. Protein extracts were separated by electrophoresis on 10% SDS-polyacrylamide gels and then transferred to PVDF membranes. Membranes were blocked with 5% nonfat dry milk in Tris-buffered saline

and 0.1% Tween 20 (Bio-Rad #170-6531, Hercules, CA) for 1 hr, and then incubated with the primary antibody overnight at 4 °C, followed by incubation with secondary antibody peroxidase-conjugated anti-mouse IgG (1:10000, GE Healthcare # NA931, RRID:AB_772210) for 1 hr. Detections were carried out using a chemiluminescence detection substrate (Thermo Fisher Scientific #32106). Primary antibodies used were mouse anti-PAX7 at 1:10, Developmental Studies Hybridoma Bank (DSHB) #PAX7, RRID:AB_528428, Iowa City, IA; mouse anti-p21$^{WAF1/CIP1}$ at 1:1000, Santa Cruz Biotechnology Cat# sc-6246, RRID:AB_628073, Dallas, TX; and HRP-conjugated GAPDH at 1:10000, Proteintech #HRP-60004, RRID:AB_2737588, Rosemont, IL.

Immunostaining on cultured cells

Cell cultures were fixed with 4% paraformaldehyde (PFA) (Sigma #P6148) for 20 min, rinsed three times in PBS, then permeabilized with 0.3% Triton X-100 (Sigma #X100) for 30 min, followed by blocking with 3% bovine serum albumin (BSA) (Thermo Fisher Scientific #BP1605-100, Waltham, MA) for 1 hr, all at room temperature. Primary antibodies were diluted in 3% BSA and incubated overnight at 4°C. Cultures were then washed three times in PBS and incubated with appropriate coupled secondary antibodies in 3% BSA for 1 hr at room temperature. Cultures were counterstained with DAPI for 15 min and washed 3 times in PBS before analysis. Primary antibodies used were mouse anti-PAX7 at 1:10, DSHB #PAX7, RRID:AB_528428; rabbit anti-MYOD1 at 1:500, Santa Cruz Biotechnology #sc-304, RRID:AB_631992, Dallas, TX; and mouse anti-MHC at 1:20, DSHB #MF-20, RRID:AB_2147781. Secondary antibodies used were: goat anti-mouse Alexa Fluor 555, goat anti-mouse Alexa Fluor 647, goat anti-rabbit

Alexa Fluor 647 (all from Life Technologies). Fluorescent images were captured with a Zeiss AxioObserver Z1 inverted microscope with an AxioCamMR3 camera using the ZEN software (Zeiss). Image processing and quantification were performed using Fiji/ImageJ.

Immunostaining on muscle sections

TA muscles were harvested for analysis 4 months after cell transplantation. Harvested TA muscles were embedded in optimal cutting temperature (OCT) solution (Scigen #4586, Gardena, CA), snap frozen in liquid nitrogen-chilled 2-methylbutane (Sigma #320404) and stored at -80°C. For EGFP staining, TA muscles were pre-fixed with 4% PFA overnight at 4°C following by overnight incubation with 20%/30% sucrose before embedding. Muscle sections were cut at 10 µm on a Leica CM3050 S cryostat (Leica Microsystems, Buffalo Grove, IL) and collected every other 250 µm across the TA muscle. Sections with the largest cross-sectional area of the whole TA were used for fiber engraftment evaluation. Sections were rehydrated with PBS, permeabilized with 0.3% Triton X-100 for 30 min and blocked with 3% BSA for 1 hr at room temperature. For PFA-fixed samples, sections were boiled in sodium citrate buffer (10 mM sodium citrate, 0.05% Tween 20, pH 6.0) at 95°C for 20 min for epitope retrieval before permeabilization. Primary antibodies were incubated overnight at 4°C followed by secondary antibodies for 1 hr at room temperature and counterstained with DAPI. Slides were mounted with Immu-Mount (Thermo Scientific #9990402) and proceeded to image capture as described above. Images showing whole TA muscles were captured in a tile mode and stitched together using the Zen software. Primary antibodies used were rabbit

anti-DYSTROPHIN (1:250, Abcam #ab15277; RRID:AB_301813), mouse anti-MHC-I (1:100, DSHB #BA-D5; RRID:AB_2235587), mouse anti-MHC-IIa (1:100, DSHB #SC-71; RRID:AB_2147165), mouse anti-MHC-IIb (1:100, DSHB #BF-F3; RRID:AB_2266724), mouse anti-PAX7 (1:10, DSHB #PAX7; RRID:AB_528428), chicken anti-EGFP (1:500, Abcam #ab13970; RRID:AB_300798), rabbit anti-laminin (1:500, Sigma #L9393; RRID:AB_477163), and Alexa Fluor 555 anti-α-bungarotoxin (1:100, Invitrogen #B35451; RRID:AB_2617152). Secondary antibodies used were goat anti-rabbit Alexa Fluor 555, goat anti-mouse Alexa Fluor 647 and donkey anti-chicken Alexa Fluor 488.

Fiber counting and area measurement

Sections with the largest cross-sectional area of the whole TA were used for fiber engraftment evaluation. Fiber counting and cross section area measurement were performed using Muscle2Veiw, a CellProfiler pipeline (Sanz et al., 2019) with adjusted parameters. DYSTROPHIN or laminin staining was used to determine the cross-sectional area of muscle fibers. Fiber engraftment is defined as the total cross-sectional area of DYSTROPHIN+ fibers over the total cross-sectional area of the whole TA section.

In situ muscle function assay

For functional evaluation of TA muscles, an *in situ* assessment of force production was performed using the Aurora 3-in-1 animal system (1300A; Aurora Scientific, Aurora, Canada), implemented with Dynamic Muscle Analysis software (615A; Aurora Scientific) for data analysis (Wu et al., 2021). Mice were maintained under anesthetized

during the whole procedure with isoflurane (Piramal Critical Care, NDC:66794-013-25, Mumbai, India). 4-5% of isoflurane were provided to the mice in the induction chamber until the mouse was recumbent, then the anesthesia was maintained with 1-2% isoflurane provided through a nose cone. The TA muscle was exposed and isolated at its distal tendon. The animal was then positioned on the aurora system and its knee was stabilized using a vertical knee pin. The TA distal tendon was attached to a force transducer and a pair of platinum-coated electrodes was placed inside the TA muscle for electrical stimulation using rectangular unipolar pulses at 0.2 ms duration and 150 Hz. TA muscle was maintained at optimal length, and the maximal tetanic force (F_0) was determined at 0.2 ms of 150 Hz stimulation for 300 ms. Fatigue time was defined as the time required for force to drop to 30% of F_0 after stimulation at 0.2 ms of 150 Hz for 2 min. Total muscle cross-sectional area (CSA) was calculated by dividing muscle mass (mg) by the product of muscle length (mm) and muscle density (1.06 mg/mm^3). Specific force (sF_0) was determined by normalizing F_0 to CSA.

Quantitative RT-PCR

Total RNA was extracted using RNeasy Mini Kit (QIAGEN #74106, Valencia, CA), and subsequent genomic DNA removal and reverse transcription (RT) were performed using Verso cDNA Synthesis Kit (Thermo Scientific #AB1453A, Pittsburgh, PA). Quantitative reverse transcription polymerase chain reaction (qRT-PCR) was performed in triplicate using TB Green Premix Ex Taq II (Takara Bio, Otsu, Japan). Expression of individual genes was subsequently analyzed by the ΔCt method in relative to the expression of the housekeeping gene *Gapdh* in a QuantStudio 6 Flex Real-Time PCR System using

QuantStudio Real-Time PCR Software (both Applied Biosystems). Primer sequences are listed as follows: *Pax7* (GTTCGGGAAGAAAGAGGACGAC, GGTTCTGATTCCACATCTGAGCC); *Myod1* (GCACTACAGTGGCGACTCAGAT, TAGTAGGCGGTGTCGTAGCCAT); *Myog* (CCATCCAGTACATTGAGCGCCT, CTGTGGGAGTTGCATTCACTGG); *Gapdh* (TTCAACGGCACAGTCAAG, CCAGTAGACTCCACGACATA).

RNA-seq

Freshly-sorted α7+ V+ cells from E14 ESC-derived teratomas (T_F), 37-day expanded teratoma-derived cells (T_E), freshly-sorted α7+ V+ satellite cells from wildtype B6 hindlimb muscles (S_F) and 37-day expanded satellite cells (S_E) were collected for RNA extraction (200,000-500,000 cells per sample, n=2 biological replicates for each group). Total RNA was extracted using RNeasy Mini Kit and in-column genomic DNA removal was applied. RNA samples with RNA integrity number (RIN) > 7 were processed for library generation. 250 pg-10 ng of total RNA was used for sequencing libraries generation using SMARTer Stranded Total RNA-Seq Kit v2 – Pico Input Mammalian Kit (Clontech #634412). Paired-end 150 base-pair sequencing was performed using an Illumina NovaSeq 6000 (Illumina, San Diego, CA), producing 20 million raw reads per sample at the University of Minnesota Genomic Center.

RNA-seq analysis

RNA-seq raw paired-end reads were aligned to Mus musculus reference transcriptome (GRCm38/mm10) using Kallisto (v0.44.0) (Bray et al., 2016) via Minnesota

Supercomputing Institute, University of Minnesota. Transcript abundance was quantified and summarized into gene level as read counts values using the R package tximport (v1.10.0) (Soneson et al., 2015). DEBrowser software (Kucukural et al., 2019) implemented with DESeq2 (Love et al., 2014) was used to perform subsequent analysis. Lowly-covered genes were filtered out from the 8 samples (only genes with at least 2 read counts per million mapped reads in at least 2 samples were included), and the read counts of filtered genes were normalized using the median ratio normalization (MRN) method for further analysis. Principal component analysis (PCA) of all detected genes was plotted using the function PCA plot. Differentially expressed genes (DEGs) were identified with fold change > 1.25 and adjusted p value < 0.05. Heatmap of DEGs was generated using the function Heatmap plot included with DEBrowser. RNA-seq datasets can be accessed on GEO: GSE182508. Pearson correlation coefficient (r) was calculated between engraftment (%) and gene expression of 4 groups to evaluate correlation.

Software

FACS analysis data acquisition were performed in FACSDiva v6.1.3 (BD) and analyzed in FlowJo v7.6.3 (FLOWJO LLC). Immunostaining images were acquired using ZEN v2.3 pro (Zeiss). Fiber counting and measurements were performed with using Muscle2Veiw, a CellProfiler pipeline (Sanz et al., 2019).

Statistical analysis

Data are presented as mean ± SEM. All statistical analyses are performed using GraphPad Prism v6.07 (GraphPad Software, La Jolla, CA). Significance is calculated

using two-tailed, unpaired Student's t tests for comparison between two groups. Differences are considered to be statistically significant at the $p < 0.05$ level.

Supplemental References

Arpke, R.W., Darabi, R., Mader, T.L., Zhang, Y., Toyama, A., Lonetree, C.L., Nash, N., Lowe, D.A., Perlingeiro, R.C., and Kyba, M. (2013). A new immuno-, dystrophin-deficient model, the NSG-mdx(4Cv) mouse, provides evidence for functional improvement following allogeneic satellite cell transplantation. Stem Cells *31*, 1611-1620.

Bray, N.L., Pimentel, H., Melsted, P., and Pachter, L. (2016). Near-optimal probabilistic RNA-seq quantification. Nat. Biotechnol. *34*, 525-527.

Chan, S.S., Arpke, R.W., Filareto, A., Xie, N., Pappas, M.P., Penaloza, J.S., Perlingeiro, R.C.R., and Kyba, M. (2018). Skeletal muscle stem cells from PSC-derived teratomas have functional regenerative capacity. Cell Stem Cell *23*, 74-85.

Ippolito, J., Arpke, R.W., Haider, K.T., Zhang, J., and Kyba, M. (2012). Satellite cell heterogeneity revealed by G-Tool, an open algorithm to quantify myogenesis through colony-forming assays. Skelet. Muscle *2*, 13.

Ismailoglu, I., Yeamans, G., Daley, G.Q., Perlingeiro, R.C., and Kyba, M. (2008). Mesodermal patterning activity of SCL. Exp. Hematol. *36*, 1593-1603.

Kucukural, A., Yukselen, O., Ozata, D.M., Moore, M.J., and Garber, M. (2019). DEBrowser: interactive differential expression analysis and visualization tool for count data. BMC Genomics *20*, 6.

Love, M.I., Huber, W., and Anders, S. (2014). Moderated estimation of fold change and dispersion for RNA-seq data with DESeq2. Genome Biol. *15*, 550.

Sanz, G., Martínez-Aranda, L.M., Tesch, P.A., Fernandez-Gonzalo, R., and Lundberg, T.R. (2019). Muscle2View, a CellProfiler pipeline for detection of the capillary-to-

muscle fiber interface and high-content quantification of fiber type-specific histology. J. Appl. Physiol. *127*, 1698-1709.

Soneson, C., Love, M.I., and Robinson, M.D. (2015). Differential analyses for RNA-seq: transcript-level estimates improve gene-level inferences. F1000Research *4*, 1521.

Wu, J., Matthias, N., Bhalla, S., and Darabi, R. (2021). Evaluation of the Therapeutic Potential of Human iPSCs in a Murine Model of VML. Mol. Ther. *29*, 121-131.
