# Supplementary material for: In vitro expanded skeletal myogenic progenitors from pluripotent stem cell-derived teratomas have high engraftment capacity
Source: Stem Cell Reports. 2021 Nov 18;16(12):2900–12. doi: 10.1016/j.stemcr.2021.10.014 (PMC8693664; doi:10.1016/j.stemcr.2021.10.014)
Supplement: Document S2. Article plus supplemental information [file mmc4.pdf]

Stem Cell Reports
Article

OPEN ACCESS

In vitro expanded skeletal myogenic progenitors from pluripotent stem cell-derived teratomas have high engraftment capacity

Ning Xie,[1,2,3] Sabrina N. Chu,[1] Karim Azzag,[4] Cassandra B. Schultz,[1] Lindsay N. Peifer,[1] Michael Kyba,[1,2,3] Rita C.R. Perlingeiro,[2,3,4] and Sunny S.K. Chan[1,2,3,*]
[1]Department of Pediatrics, University of Minnesota, 2231 6th Street SE, Cancer and Cardiovascular Research Building, Minneapolis, MN 55455 USA
[2]Stem Cell Institute, University of Minnesota, Minneapolis, MN, USA
[3]Lillehei Heart Institute, University of Minnesota, Minneapolis, MN, USA
[4]Department of Medicine, University of Minnesota, Minneapolis, MN, USA
*Correspondence: sschan@umn.edu
https://doi.org/10.1016/j.stemcr.2021.10.014

SUMMARY

One major challenge in realizing cell-based therapy for treating muscle-wasting disorders is the difficulty in obtaining therapeutically meaningful amounts of engraftable cells. We have previously described a method to generate skeletal myogenic progenitors with exceptional engraftability from pluripotent stem cells via teratoma formation. Here, we show that these cells are functionally expandable *in vitro* while retaining their *in vivo* regenerative potential. Within 37 days in culture, teratoma-derived skeletal myogenic progenitors were expandable to a billion-fold. Similar to their freshly sorted counterparts, the expanded cells expressed PAX7 and were capable of forming multinucleated myotubes *in vitro*. Importantly, these cells remained highly regenerative *in vivo*. Upon transplantation, the expanded cells formed new DYSTROPHIN$^+$ fibers that reconstituted up to 40% of tibialis anterior muscle volume and repopulated the muscle stem cell pool. Our study thereby demonstrates the possibility of producing large quantities of engraftable skeletal myogenic cells for transplantation.

INTRODUCTION

Satellite cells, also known as muscle stem cells, are the primary cells responsible for muscle regeneration (Günther et al., 2013; von Maltzahn et al., 2013). Satellite cells reside between the basal lamina and the sarcolemma of muscle fibers (Mauro, 1961), are normally quiescent, and express the transcription factor Pax7 (Schultz et al., 1978; von Maltzahn et al., 2013). In response to muscle injuries, satellite cells are activated, reenter the cell cycle, rapidly proliferate, differentiate into myoblasts, and fuse to form multinucleated myofibers (Cooper et al., 1999; Snow, 1977). In addition, some satellite cells become quiescent again and repopulate the muscle stem cell pool (Zammit et al., 2004). Satellite cells have tremendous *in vivo* regenerative capability. A single satellite cell is capable of regenerating damaged skeletal muscles by reconstituting both the fiber and the muscle stem cell compartments (Collins et al., 2005; Sacco et al., 2008). Therefore, transplantation of healthy satellite cells is a promising approach for treating skeletal muscle-wasting disorders such as Duchenne muscular dystrophy.

Generating sufficient engraftable cells is a major challenge for realizing cell-based therapy (Blau and Daley, 2019). Satellite cells are scarce, representing only 1% to 2% of mononuclear cells in skeletal muscles (Roth et al., 2000). A therapeutically meaningful amount of satellite cells is thereby unlikely to be obtained from small skeletal muscle biopsies. This problem can be theoretically solved by *in vitro* expansion of satellite cells. However, the robust regenerative potency of satellite cells is lost once they are isolated and grown in cultures. For mouse satellite cells, a 3-day culture led to a 10-fold decrease in engraftment potential (Montarras et al., 2005; Sacco et al., 2008). Cultured canine myoblasts produced poorer engraftment compared with freshly isolated satellite cells (Parker et al., 2012). Human myoblasts expanded *in vitro* also showed low engraftment efficiency and failed to replenish the muscle stem cell pool after transplantation into Duchenne muscular dystrophy patients (Gussoni et al., 1992; Mendell et al., 1995). Optimization of the culturing conditions can alleviate this problem to a certain extent. Satellite cells cultured in hydrogels or artificial niches that resemble the elasticity of the native skeletal muscle environment had improved engraftability (Gilbert et al., 2010; Quarta et al., 2016). Similarly, modulation of the Notch and p38 signaling pathways restored the regenerative potential of cultured satellite cells after transplantation (Charville et al., 2015; Parker et al., 2012). However, these approaches were usually performed within a relatively short period of time in cultures. The validity of long-term *in vitro* expansion of engraftable skeletal myogenic cells remains unresolved.

Pluripotent stem cells (PSCs), including embryonic stem cells (ESCs) and induced pluripotent stem cells (iPSCs), possess great promise for cell therapy targeting degenerating muscles (Chal and Pourquié, 2017). PSCs have

This is an open access article under the CC BY license (http://creativecommons.org/licenses/by/4.0/).

theoretically unlimited proliferative potential, thereby allowing generation of sufficient skeletal myogenic progenies for transplantations. Currently there are two main approaches to derive skeletal myogenic cells from PSCs *in vitro* (Chal and Pourquié, 2017; Jiwlawat et al., 2018). Overexpression of skeletal myogenic transcription factors such as PAX3 or PAX7 can efficiently differentiate PSCs into skeletal myogenic progenitors that engraft to form force-generating muscle fibers (Darabi et al., 2012; Filareto et al., 2013). On the other hand, non-transgenic approaches employ various growth factors and small molecules to direct skeletal myogenic differentiation from PSCs (Chal et al., 2015; Shelton et al., 2014). However, skeletal myogenic cells derived from monolayer differentiation do not reliably engraft without further purification, making evaluation of the functionality of the regenerated muscles difficult (Hicks et al., 2018). Also, whether monolayer differentiation-derived skeletal myogenic cells can be passaged and expanded while maintaining their already modest engraftability remains unclear (Al Tanoury et al., 2020).

We recently developed a novel method to differentiate mouse PSCs into skeletal myogenic progenitors via teratoma formation (Chan et al., 2018). On a cell-to-cell basis, teratoma-derived skeletal myogenic progenitors are functionally similar to endogenous satellite cells in forming muscle fibers. Also, these teratoma-derived cells repopulate the muscle stem cell pool and are responsive to subsequent injuries for a secondary regeneration. Here, we show that teratoma-derived skeletal myogenic progenitors are readily expandable *in vitro* while retaining significant parts of their robust *in vivo* regenerative capacity.

RESULTS

Teratoma-derived skeletal myogenic progenitors are expandable *in vitro*

We have previously reported that mouse PSC-derived teratomas are rich in skeletal myogenic progenitors with exceptional regenerative potency (Chan et al., 2018). Because it is more feasible to expand a purified skeletal myogenic population than to perform a large-scale differentiation operation followed by a massive purification step for each transplant, it is imperative to explore the *in vitro* expandability of teratoma-derived skeletal myogenic progenitors (Figure 1A). We first induced teratoma formation by implanting EGFP-labeled mouse ESCs (E14-EGFP ESCs) into irradiated and cardiotoxin-injured tibialis anterior (TA) muscles of NSG-mdx^{4Cv} mice as previously described (Chan et al., 2018). At 4 weeks, teratomas were harvested and EGFP$^+$ (teratoma-derived) skeletal myogenic progenitors, defined as lineage-negative (Lin$^-$) (CD31$^-$ and CD45$^-$, i.e., non-endothelial and non-hematopoietic, respectively), α7-integrin$^+$, and VCAM-1$^+$ (α7$^+$ VCAM$^+$), were isolated by fluorescence-activated cell sorting (FACS) (Figures 1A and S1A) (Chan et al., 2018). We then plated and cultured these freshly sorted cells in a pro-proliferation medium (Figure 1A). Teratoma-derived skeletal myogenic progenitors maintained a steady growth and were amplified by 9 orders of magnitude within 37 days over eight passages (Figure 1B). We subsequently compared the skeletal myogenic characteristics of the passage 8 cells (expanded cells) with their freshly sorted counterparts (fresh cells).

We first determined whether the expression of α7 and VCAM was altered during the expansion process (Figures 1C and S1B). From the time points we investigated (up to day 37, or passage 8), most cells stably expressed α7 (98.1%–99.1%, 95% confidence interval; n = 3 independent experiments). This suggested that our culture system promoted a predominately skeletal myogenic population with limited non-myogenic potential (e.g., brown adipocytes [Seale et al., 2008]). Among the α7$^+$ population, a majority (≥75%) were also VCAM$^+$ (Figure 1D), suggesting a muscle stem cell signature (Chan et al., 2013). Indeed, compared with α7$^+$ VCAM$^-$ resorted cells, α7$^+$ VCAM$^+$ cells sorted from passage 8 cultures expanded more readily and had a higher expression of muscle stem cell factor *Pax7* but a lower expression of skeletal muscle specification factors *Myod1* and *Myog* (Figure S1C and S1D). Importantly, α7$^+$ VCAM$^+$ resorted from passage 8 cultures engrafted more reliably than α7$^+$ VCAM$^-$ cells (Figure S1E). On the other hand, α7$^+$ VCAM$^-$ cells may represent a skeletal myogenic-committed subpopulation that was differentiating into myoblasts (Giordani et al., 2019).

Expanded teratoma-derived α7$^+$ VCAM$^+$ cells remain highly skeletal myogenic

Quiescent satellite cells in adult skeletal muscles express the transcription factor PAX7 (von Maltzahn et al., 2013). We observed comparable expression of the PAX7 protein in both freshly sorted and expanded teratoma-derived α7$^+$ VCAM$^+$ cells, suggesting that the expanded cells remained mostly undifferentiated (Figure 2A). In contrast, PAX7 was significantly reduced in endogenous satellite cells after *in vitro* expansion for eight passages (Figures S2A and S2B).

Once isolated and in culture (or in response to injury *in vivo*), satellite cells become activated and begin to express the skeletal myogenic specification factor MYOD1 (Brack et al., 2008; Tierney et al., 2016). Indeed, after 3 days of culture, cells in both groups started to express MYOD1 (Figure 2B). A majority of them were PAX7$^+$ MYOD1$^+$ (fresh, 92.7% ± 0.6%; expanded, 96.4% ± 1.3%; n = 3 independent experiments, $p > 0.05$), indicating a predominately activated/proliferating population as expected. Notably, a

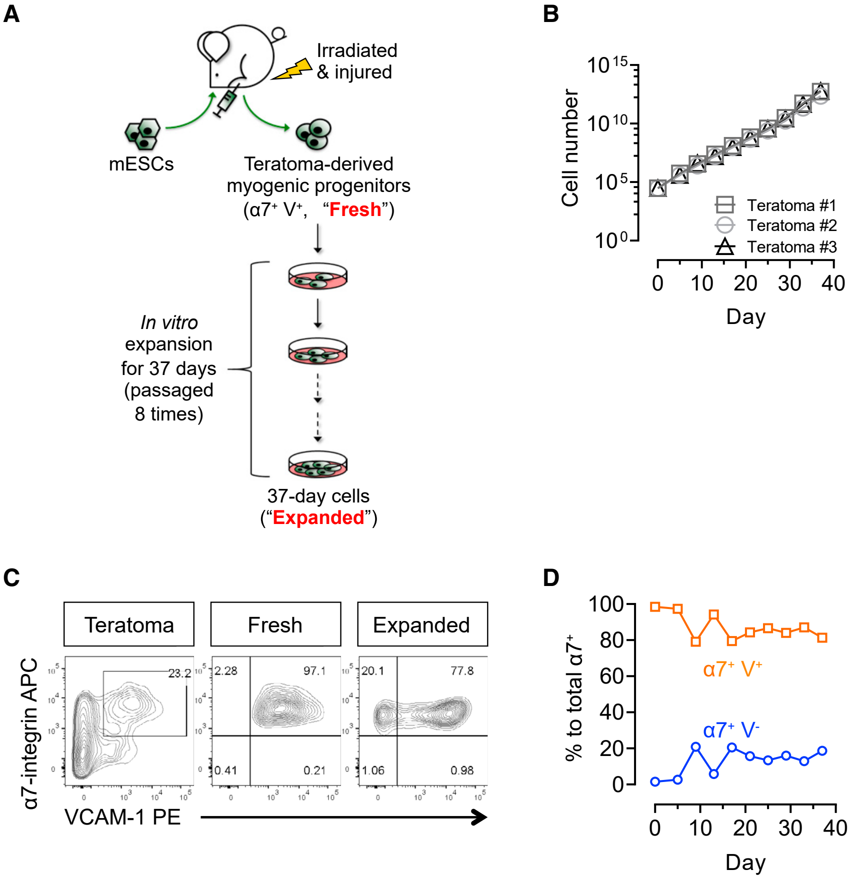

Figure 1. Teratoma-derived skeletal myogenic progenitors are expandable *in vitro*

(A) Schematic of *in vitro* expansion of teratoma-derived skeletal myogenic progenitors. Four-week EGFP-labeled mouse ESC-derived teratomas were harvested and FACS sorted for the skeletal myogenic population: $CD31^-$ $CD45^-$ α7-integrin$^+$ VCAM-1$^+$ (α7$^+$ V$^+$) cells. The freshly sorted α7$^+$ V$^+$ cells ("Fresh") were subsequently cultured and expanded for another 37 days over 8 passages ("Expanded").

(B) *In vitro* cultures of α7$^+$ V$^+$ cells showed exponential growth for up to 37 days. Data are shown as the mean ± SEM of three independent experiments.

(C) FACS profiling of total teratoma cells, freshly sorted α7$^+$ V$^+$ cells, and expanded 37-day cells (representative of three independent experiments).

(D) Quantitation of α7$^+$ V$^+$ and α7$^+$ V$^-$ cells by FACS during *in vitro* expansion. Data are shown as the mean ± SEM of three independent experiments.

ESCs, embryonic stem cells; α7, α7-integrin; V, VCAM-1.

See also Figure S1.

small but significant subpopulation of cells remained in an inactivated PAX7$^+$ MYOD1$^-$ state (fresh, 5.0% ± 0.6%; expanded, 1.6% ± 0.7%; n = 3 independent experiments, $p < 0.05$). PAX7$^-$ MYOD1$^+$-committed myoblasts were also observed (fresh, 2.4% ± 0.3%; expanded, 2.0% ± 0.6%; n = 3 independent experiments, $p > 0.05$).

We next probed the skeletal myogenic differentiation potential of these cells. In serum-depleted conditions, proliferating skeletal myogenic progenitors differentiated into myosin heavy chain-positive (MHC$^+$) myoblasts, which subsequently fused into multinucleated myotubes. We found that both freshly sorted and expanded cells were equally adept at developing into myoblasts and fusing into myotubes (Figure 2C).

In culture, single satellite cells are capable of forming individual colonies (Seale et al., 2001). To test the clonality of teratoma-derived skeletal myogenic progenitors, we seeded single freshly sorted and expanded cells by FACS into 96-well plates and cultured them in a medium that supports both proliferation and differentiation (Ippolito et al., 2012). After 8 days, we observed a similar clonal efficiency between the two groups (Figure 2D). Nevertheless, colonies from freshly sorted cells appeared to consist of more myonuclei than expanded cells (Figure 2D). Therefore, on a cell-to-cell basis, freshly sorted cells and expanded cells have comparable proficiency in forming MHC$^+$ colonies, although colonies from the latter cells were at a smaller size. Altogether, our results showed that *in vitro* expansion did not fundamentally alter the skeletal myogenic nature of teratoma-derived α7$^+$ VCAM$^+$ cells.

Expanded teratoma-derived skeletal myogenic progenitors engraft and differentiate into muscle fibers upon transplantation

To assess the *in vivo* regenerative potential of expanded teratoma-derived α7$^+$ VCAM$^+$ cells, we performed intramuscular transplantations (Figure 3A). We transplanted the freshly sorted or the expanded teratoma-derived skeletal myogenic progenitors into the TA muscles of NSG-mdx^{4Cv} mice (Arpke et al., 2013). These animals are immunocompromised (from the NSG background) and their muscles lack dystrophin (from the mdx^{4Cv} background), thereby allowing allogeneic cell transplantations and identification of donor-derived fibers (DYSTROPHIN$^+$). Also, the TA muscles were irradiated and cardiotoxin-injured to create a permissive environment for cell

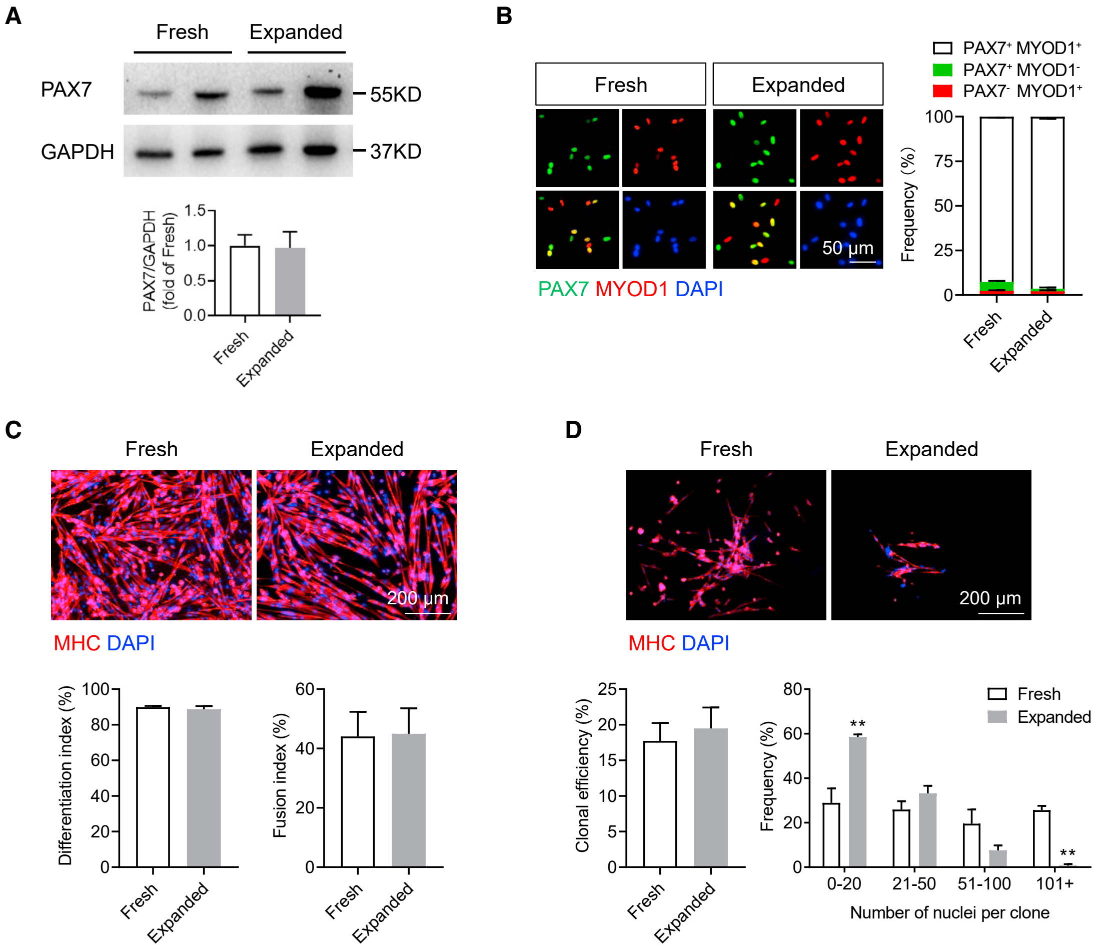

Figure 2. *In vitro* characterization of expanded teratoma-derived skeletal myogenic progenitors

(A) Immunoblots (top) and quantification (bottom) showing that both freshly sorted and expanded cells expressed the muscle stem cell transcription factor PAX7 (two independent samples from each group are shown). Data are shown as the mean ± SEM of five independent experiments.

(B) Immunostaining (left) and quantification (right) showing the expression of PAX7 and MYOD1 in 3-day cultures of freshly sorted and expanded cells. Scale bar represents 50 μm. Data are shown as the mean ± SEM of three independent experiments.

(C) Immunostaining (top) of MHC in freshly sorted and expanded cells cultured in differentiation medium. Scale bar represents 200 μm. Quantification of differentiation (bottom left) and fusion in MHC$^+$ myotubes (bottom right) from three independent experiments. Data are shown as the mean ± SEM.

(D) Clonal analysis (top) showing that both single freshly sorted and expanded cells were capable of forming MHC$^+$ skeletal myogenic colonies. Scale bar represents 200 μm. Quantification of clonal efficiency (bottom left) and clonal size distribution (bottom right) from three independent experiments and 170 single cells per experiment. **$p < 0.01$. Data are shown as the mean ± SEM.

α7, α7-integrin; V, VCAM-1; MHC, myosin heavy chain.

See also Figure S2.

transplantation (Chan et al., 2018). We transplanted into each TA 40,000 cells, a number we have previously determined to be functionally equivalent to the amount of endogenous PAX7$^+$ satellite cells in a single TA muscle (Brack et al., 2005; Chan et al., 2018), and evaluated fiber engraftment after 4 months (Figures 3B and 3C). As a direct comparison, we also transplanted freshly isolated and expanded (day 37, or passage 8) endogenous satellite cells harvested from adult muscles (Figures 3B, 3C, and S2A). Freshly sorted teratoma-derived α7$^+$ VCAM$^+$ cells displayed robust fiber engraftment, regenerating ~80% of the TA muscle. This level of engraftment is almost equivalent to endogenous satellite cells freshly isolated from adult muscles. Intriguingly, expanded teratoma-derived skeletal myogenic progenitors also produced a significant number of DYSTROPHIN$^+$ fibers, reconstituting >40% of the total

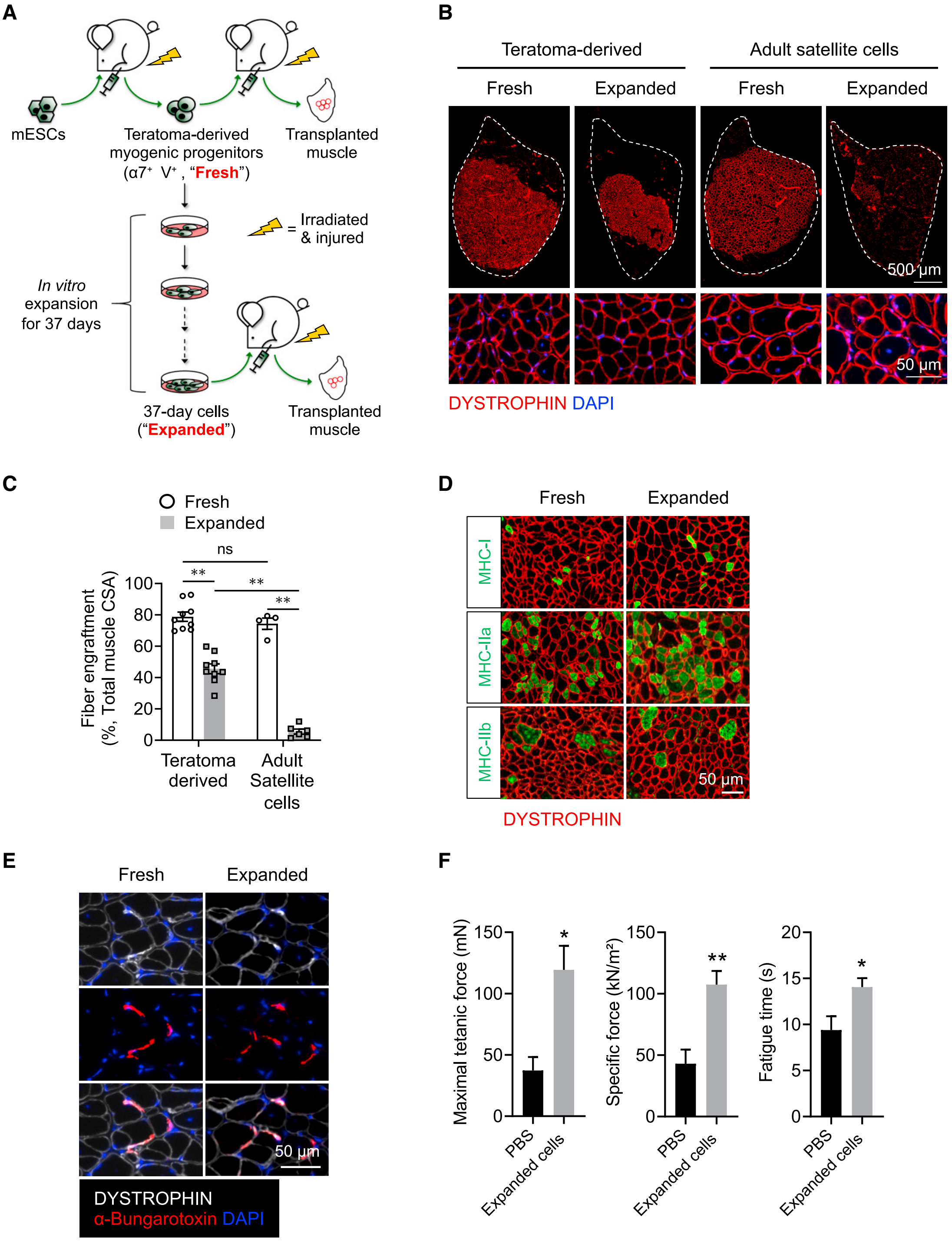

Figure 3. Expanded teratoma-derived skeletal myogenic progenitors engraft and form new muscle fibers

(A) Schematic of evaluation of the engraftability of freshly sorted and expanded teratoma-derived skeletal myogenic progenitors.

(B) Freshly sorted (far left) and expanded (middle left) teratoma-derived skeletal myogenic progenitors engrafted and formed DYSTOPHIN^{+} fibers 4 months post-transplant. In contrast, freshly sorted adult satellite cells (middle right) engrafted, but their expanded counterparts (far right) did not. The whole TA muscle is outlined (top, scale bar represents 500 μm), and magnified images are shown (bottom, scale bar represents 50 μm). Representative images from four to nine biological replicates.

(legend continued on next page)

muscle volume. This is particularly exciting, as endogenous satellite cells rapidly lost their engraftment capability (to ~5%) once they were in culture (Figures 3B and 3C and Montarras et al., 2005; Sacco et al., 2008).

The remarkable engraftment potential of teratoma-derived skeletal myogenic progenitors that have been cultured for 37 days (passage 8) prompted us to determine whether these cells remain engraftable if they were expanded to a longer time point. To address this, we cultured teratoma-derived skeletal myogenic progenitors in a dish for 79 days to passage 18. These passage 18 cells expanded readily, were mainly $\alpha7^+$ $VCAM^+$, expressed PAX7, and were capable of differentiating into multinucleated myotubes (Figures S3A–S3D). Remarkably, passage 18 cells remained highly regenerative *in vivo*, with an engraftment level similar to passage 8 cells (Figures S3E and S3F).

The above results were obtained using E14 ESCs. To evaluate whether the expandability of teratoma-derived skeletal myogenic progenitors is also applicable to other PSC lines, we tested C57BL/6N-PRX-B6N #1 ESCs and Pax7-ZsGreen iPSCs (Chan et al., 2018). We found that teratoma-derived $\alpha7^+$ $VCAM^+$ cells obtained from these PSC lines were expandable (at least up to passage 8) and remained highly skeletal myogenic in forming myotubes *in vitro* and in regenerating new muscle fibers after transplantation (Figures S4A–S4H).

Newly formed fibers in muscles transplanted with expanded teratoma-derived skeletal myogenic progenitors are functional

Adult skeletal muscles consist of multiple fiber types, including slow-twitch (MHC-I) and fast-twitch (MHC-IIa and MHC-IIb) isoforms. To determine the maturity of the regenerated muscles, we evaluated their fiber type composition. Immunostaining of the newly formed muscles revealed that all of these adult myosin isoforms were present at varying degrees in $DYSTROPHIN^+$ fibers derived from both freshly sorted and expanded cells (Figures 3D and S4I). Measurement of individual fiber cross-sectional area also showed similar fiber size distributions between the two cell groups (Figure S4J).

Innervation of newly formed fibers is essential to their maturation and longevity. We undertook staining with α-bungarotoxin, which binds to the nicotinic acetylcholine receptors in the post-synaptic membrane of the neuromuscular junction. We observed a close proximity of α-bungarotoxin immunostaining to $DYSTROPHIN^+$ fibers derived from both freshly sorted and expanded cells, suggesting a potential presence of neuromuscular junctions in these newly formed fibers (Figure 3E).

Next, we evaluated whether the expanded cells were capable of endowing functional improvement to the transplanted muscles. Four months post-transplant, we performed physiological assessment to the transplanted TA muscles *in situ*. Comparing with the PBS-injected control muscles, we observed a significant improvement in maximal tetanic force, specific force, and fatigue time in muscles transplanted with expanded teratoma-derived skeletal myogenic progenitors (Figure 3F). These results indicated that the regenerated muscles were functional and capable of force generation.

Expanded teratoma-derived skeletal myogenic progenitors repopulate the muscle stem cell compartment upon transplantation

The long-term maintenance of the regenerated muscles is ultimately determined by the ability of the transplanted cells to reconstitute the muscle stem cell pool. We first performed immunostaining on sections of the transplanted muscles with antibodies against EGFP (donor cells), PAX7 (satellite cells), and laminin (sarcolemma). We readily observed $EGFP^+$ $PAX7^+$ cells under the muscle basal lamina ($laminin^+$) from both freshly sorted and expanded cell transplantations (Figure 4A). This indicated that the transplanted cells adopted a satellite cell fate and repopulated the muscle stem cell niche.

We wished to further quantify the extent to which teratoma-derived skeletal myogenic progenitors reconstituted the muscle stem cell compartment. We evaluated the mononuclear fraction of the transplanted muscles by FACS for signs of muscle stem cell contribution. Remarkably, the cultured cells gave rise to as many $\alpha7^+$ $VCAM^+$

(C) Quantification of fiber engraftment ($DYSTROPHIN^+$ fibers) in transplanted TA muscles (n = 4–9 biological replicates). Data are shown as the mean ± SEM. $^{**}p < 0.01$; ns, not significant.
(D) Newly formed $DYSTROPHIN^+$ muscle fibers derived from freshly sorted and expanded cells consisted of slow-twitch (MHC-I) and fast-twitch (MHC-IIa and MHC-IIb) fibers (representative images from six biological replicates). Scale bar represents 50 μm.
(E) Potential presence of neuromuscular junctions as revealed by close proximity of α-bungarotoxin staining to newly formed $DYSTROPHIN^+$ fibers derived from freshly sorted and expanded cells (representative images from three biological replicates). Scale bar represents 50 μm.
(F) *In situ* physiological assessment revealed functional improvement 4 months after transplantation of expanded teratoma-derived skeletal myogenic progenitors (n = 5–9 biological replicates). $^{*}p < 0.05$, $^{**}p < 0.01$ versus PBS (vehicle). ESCs, embryonic stem cells; α7, α7-integrin; V, VCAM-1; CSA, cross-sectional area.
See also Figures S2–S4.

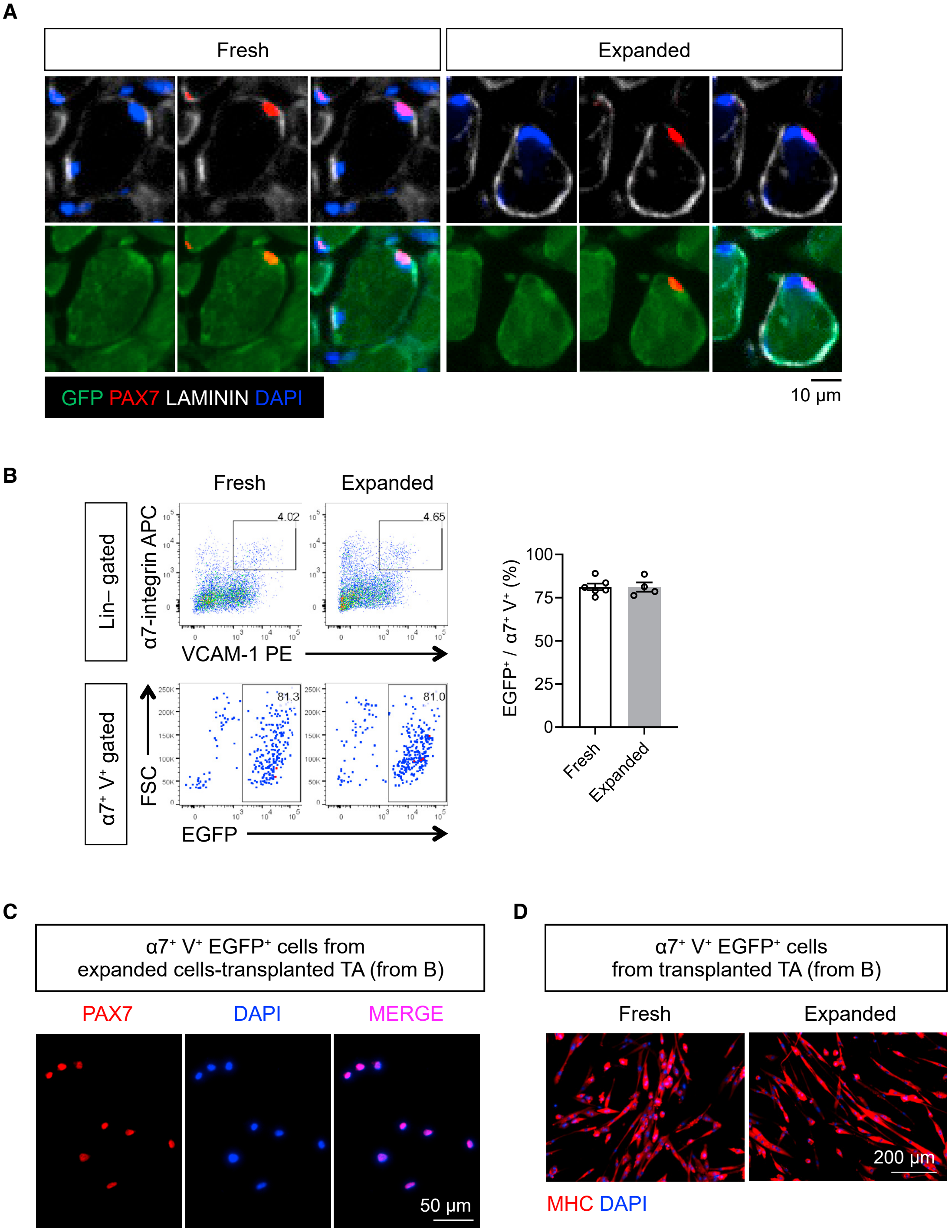

Figure 4. Expanded teratoma-derived skeletal myogenic progenitors reconstitute the muscle stem cell pool after transplantation

(A) Immunostaining showing the presence of donor-derived EGFP^{+} PAX7^{+} putative muscle stem cells under the basal lamina in transplanted muscles (representative images from six biological replicates). Scale bar represents 10 μm.

(B) FACS analysis (left) and quantification (right; n = 4–6 biological replicates) of transplanted muscles revealed that the majority of Lin^{-} (CD31^{-} CD45^{-}) α7^{+} V^{+} muscle stem cells are also EGFP^{+}, i.e., donor derived. Data are shown as the mean ± SEM.

(legend continued on next page)

mononuclear cells as the freshly isolated teratoma cells (Figure 4B). These reisolated EGFP$^+$ $\alpha7^+$ VCAM$^+$ cells expressed PAX7 (Figure 4C) and were capable of differentiating into MHC$^+$ multinucleated myotubes upon subsequent culture (Figure 4D). Altogether, our results demonstrate that teratoma-derived skeletal myogenic progenitors have an unprecedented level of *in vitro* expansion potential while maintaining a very high potency for muscle regeneration after transplantation.

Several factors may contribute to the engraftability of expanded teratoma-derived skeletal myogenic progenitors

As illustrated above, expanded teratoma-derived skeletal myogenic progenitors had an engraftment capacity superior to that of expanded adult satellite cells cultured in the same conditions (Figures 3B and 3C). The difference in engraftability might be due to relative PAX7 expression, as *in vitro* expansion over eight passages reduced PAX7 levels in adult satellite cells but not in teratoma-derived skeletal myogenic progenitors (Figures 2A and S2B). Cell senescence might also be a contribution factor, as p21$^{Waf1/Cip1}$ started to emerge in the expanded but not in the freshly isolated teratoma-derived $\alpha7^+$ VCAM$^+$ cells (Figure S5A). On the other hand, both freshly isolated and expanded teratoma-derived skeletal myogenic progenitors had similar profiles in myotube formation potential (Figure 2C) and cell-cycle stages (Figure S5B).

To gain further insights into why teratoma-derived skeletal myogenic progenitors have such a high expandability and functionality, we performed an RNA-sequencing (RNA-seq) experiment using four groups of skeletal myogenic cells: fresh teratoma-derived (T_F), expanded (day 37/passage 8) teratoma-derived (T_E), fresh satellite (S_F) (all three engraftable), and expanded (day 37/passage 8) satellite cells (S_E) (much less engraftable) (Figure 5A). From principal-component analysis, it is intriguing to see that the three engraftable populations are relatively spread out and that the two expanded cell populations are very close to each other, even though one is engraftable and the other is not (Figure 5B). Comparisons between the three engraftable populations (T_F, T_E, and S_F) and the non-engraftable population (S_E) revealed 240 upregulated genes and 283 downregulated genes (Figures 5C and 5D; Table S1).

We reasoned that factors that regulate engraftability may have their expression directly correlated to engraftment potential, that is, samples with the best engraftment would contain the highest level of engraftment-promoting genes and vice versa. We therefore calculated the Pearson correlation coefficient between engraftment and gene expression and looked for genes with a positive correlation ($r > 0.9$) (Figure S5C; Table S2). From this analysis, we found *Spry1* (Sprouty1) expression to be highly correlated to engraftment: it is minimally expressed in S_E (minimal engraftment), moderately expressed in T_E (modest engraftment), and highly expressed in S_F and T_F (both have the best engraftment) (Figure 5E). Importantly, Sprouty1 has been reported as a key regulator of satellite cell quiescence and self-renewal (Shea et al., 2010). Also, satellite cells that lack Sprouty1 were previously shown to undergo apoptosis during muscle regeneration (Shea et al., 2010), which may explain why cultured satellite cells that minimally express Sprouty1 do not engraft reliably (this study and Sacco et al., 2008). Altogether, Sprouty1, Pax7, and cell senescence might contribute to the engraftability of expanded teratoma-derived skeletal myogenic progenitors.

DISCUSSION

Differentiation of PSCs into skeletal myogenic cells that can reliably engraft has been difficult. We have previously shown that skeletal myogenic progenitors obtained from PSC-derived teratomas have exceptional *in vivo* regenerative potency in forming new fibers and repopulating the muscle stem cell pool (Chan et al., 2018). Here, we have further extended these findings in showing that teratoma-derived skeletal myogenic progenitors are capable of tremendous *in vitro* expansion without losing their remarkable regenerative power after transplantation.

We have provided multiple lines of *in vitro* and *in vivo* evidence in showing that expanded teratoma-derived skeletal myogenic progenitors have muscle stem cell characteristics. The expanded cells were PAX7$^+$, expressed the muscle stem cell surface markers $\alpha7$ and VCAM, and were capable of forming MHC$^+$ colonies with multinucleated myotubes from single cells (i.e., clonal). Most importantly, the expanded cells remained highly engraftable. When culture-expanded cells were transplanted, they differentiated into new force-generating fibers with adult myosins, and developed into PAX7$^+$ muscle stem cells residing under the basal lamina. These results suggest that the expanded teratoma-derived skeletal myogenic progenitors retain a muscle stem cell identity.

(C) Reisolated $\alpha7^+$ V$^+$ EGFP$^+$ cells (from [B]) from expanded cell-transplanted TA muscles expressed PAX7 (representative images from three biological replicates). Scale bar represents 50 μm.
(D) Reisolated $\alpha7^+$ V$^+$ EGFP$^+$ cells (from [B]) differentiated into multinucleated MHC$^+$ myotubes in cultures (representative images from four to six biological replicates). Scale bar represents 200 μm.
$\alpha7$, $\alpha7$-integrin; V, VCAM-1.

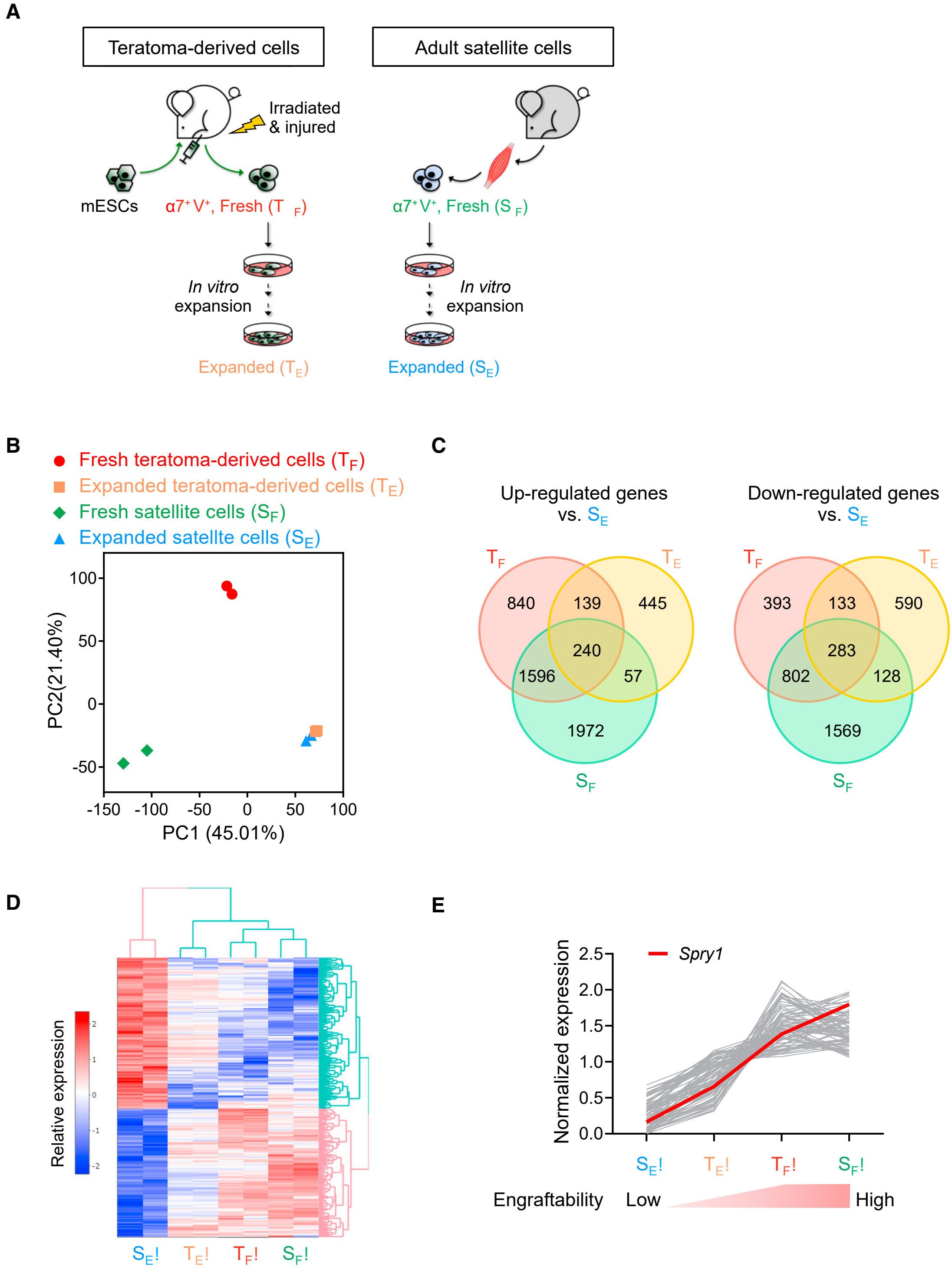

Figure 5. RNA-seq analysis to discover factors that might regulate the engraftability of skeletal myogenic progenitors

(A) Schematic of samples used for RNA-seq analysis: freshly isolated (T_F) and expanded (T_E) teratoma-derived skeletal myogenic progenitors and freshly isolated (S_F) and expanded (S_E) adult satellite cells (n = 2 biological replicates from each group).

(B) Principal-component analysis. Note that the transcriptomes of the three engraftable cell populations (T_F, T_E, and S_F) are relatively spread out and the two expanded cell populations (T_E and S_E) are very close to each other.

(legend continued on next page)

It should be noted that although the expanded teratoma-derived skeletal myogenic progenitors remained highly regenerative, their fiber engraftment potency was reduced somewhat compared with the freshly sorted teratoma cells. This modest reduction in engraftability could not be adequately explained by most *in vitro* assays, as cells from both groups were comparably potent in expressing the pro-myogenic factors PAX7 and MYOD1 and in differentiating into multinucleated MHC$^+$ myotubes. In fact, these *in vitro* features are common to skeletal myogenic populations obtained via various PSC differentiation methods, even though their *in vivo* regenerative potentials differ substantially (Chal et al., 2015; Charville et al., 2015; Gilbert et al., 2010; Hicks et al., 2018; Montarras et al., 2005; Parker et al., 2012; Quarta et al., 2016; Shelton et al., 2014). So far, the field lacks an *in vitro* assay that accurately predicts the *in vivo* engraftability of a putative skeletal myogenic population derived from PSCs. Among the *in vitro* experiments we have performed, we found in the clonal assay that the freshly sorted and the expanded teratoma-derived cells behaved somewhat differently. In the clonal assay, single cells were individually sorted and seeded by FACS, and their ability to survive, self-renew, proliferate, and differentiate into MHC$^+$ colonies was collectively evaluated after 8 days in culture. We found that although single cells from both groups had similar cloning efficiencies, colonies developed from the freshly sorted cells were generally larger in size. We reasoned that the proficiency in fiber engraftment from the transplanted cells is governed not only by their potential to express the relevant myogenic factors and undergo proper differentiation but also by their ability to survive in the transplanted muscle milieu. In this regard, the harsh environment in the irradiated and cardiotoxin-damaged muscles is no more unforgiving to the transplanted cells than the stress induced by FACS isolation and the foreign culture conditions is to the single cells. Therefore, the *in vitro* survival/self-renewal capacity of a testing cell population as revealed in the clonal assay might correlate with its *in vivo* engraftment efficiency (Stuelsatz et al., 2015). We reason that clonal analysis might be an effective predictor of engraftability after transplantation.

Large-scale expansion of skeletal myogenic progenitors with high regenerative potential is important for cell therapy applications (Blau and Daley, 2019). Recent clinical trials on cell therapy for treating muscular disorders tested tens to hundreds of millions of cells (Gussoni et al., 1992; Karpati et al., 1993; Mendell et al., 1995; Périé et al., 2014; Skuk et al., 2006, 2007). For example, in a 2014 oculopharyngeal muscular dystrophy trial, an average of 178 million myogenic cells were focally injected to achieve improvement in swallowing (Périé et al., 2014). However, *in vitro* expansion of muscle stem cells is difficult because of the dramatic loss of regenerative capacity after *in vitro* culture (Gussoni et al., 1992; Mendell et al., 1995; Montarras et al., 2005; Sacco et al., 2008). Published methods to optimize the culture conditions to enhance engraftability are so far limited to short-term expansion, and the regenerative potency of the expanded cells thus derived is relatively modest (Charville et al., 2015; Cosgrove et al., 2014; Parker et al., 2012; Quarta et al., 2016). Our study shows that skeletal myogenic progenitors obtained from PSC-derived teratomas can overcome this problem. Over a 37-day culture, teratoma-derived skeletal myogenic progenitors maintained a steady growth and expanded over 1 billion-fold, and remarkably, the expanded cells still reliably engrafted. It is conceivable that these cells could grow even longer in culture. Transplantation of 40,000 expanded cells regenerated more than 40% of the recipient muscle. This level of engraftment from muscle cells in culture is unprecedented—a 3-day *in vitro* culture of endogenous satellite cells dramatically diminished their engraftability by >10-fold, resulting in a modest 300 fibers even though 100,000 cells were transplanted (Montarras et al., 2005). Furthermore, our RNA-seq revealed factors such as Sprouty1, which may regulate the engraftability of expanded teratoma-derived skeletal myogenic progenitors. These factors might be potential candidates to promote *in vitro* expansion of endogenous muscle stem cells with preserved engraftability. We are currently investigating whether the teratoma method to produce expandable and engraftable skeletal myogenic cells is also applicable to human PSCs.

In the current study, we have demonstrated the functional expansion of an engraftable skeletal myogenic population. The possibility of culturing muscle stem cells over a prolonged period of time enables extensive genetic manipulation, which in turn is fundamental to combining gene and cell therapies to treat muscle disorders (Amoasii et al., 2018; Konieczny et al., 2013). Further investigations to address the molecular mechanisms underlying the high regenerative capacity and *in vitro* expandability of teratoma-derived skeletal myogenic progenitors would provide

(C) Venn diagrams showing differentially expressed genes (fold change >1.25, adjusted $p < 0.05$) that are commonly upregulated (left) or downregulated (right) in the three engraftable cell populations (T_F, T_E, and S_F) versus the non-engraftable cells (S_E).
(D) Heatmap showing the 240 upregulated and 283 downregulated genes from (C).
(E) Expression of upregulated genes with a Pearson correlation coefficient of >0.9 (gray lines) in the four cell populations. Expression of *Spry1* is shown in red. See text for details.
See also Figure S5 and Tables 1 and S2.

valuable insights to functionally expand endogenous muscle stem cells *in vitro*.

EXPERIMENTAL PROCEDURES

Details can be found in the supplemental experimental procedures.

Animals

All animal procedures were performed according to the University of Minnesota Institutional Animal Care and Use Committee guidelines and approved protocols.

Teratoma formation, cell transplantation, and engraftment evaluation

Recipient NSG-mdx^{4Cv} mice with their hindlimbs irradiated and TA muscles cardiotoxin-injured were used for both teratoma formation and skeletal myogenic cell transplantations. For teratoma formation, 250,000 ESCs were injected into the TA muscle. Teratomas were harvested 4 weeks later. For cell transplantations, 40,000 freshly isolated or cultured/expanded cells derived from teratomas or adult muscles were transplanted into the TA muscle. Transplanted muscles were harvested and analyzed 4 months later.

RNA-seq analysis

Paired-end 150-bp sequencing libraries were created using the SMARTer Stranded Total RNA-Seq Kit v.2-Pico Input Mammalian Kit (Clontech, Mountain View, CA). Differentially expressed genes were identified with fold change >1.25 and adjusted $p < 0.05$.

Data and code availability

The accession number for the data reported in this paper is GEO: GSE182508.

SUPPLEMENTAL INFORMATION

Supplemental information can be found online at https://doi.org/10.1016/j.stemcr.2021.10.014.

AUTHOR CONTRIBUTIONS

Conceptualization: S.S.K.C.; methodology: N.X., M.K., R.C.R.P., and S.S.K.C.; investigation: N.X., S.N.C., K.A., C.S., and L.N.P.; formal analysis: N.X.; visualization: N.X. and S.S.K.C.; writing: N.X. and S.S.K.C.; funding acquisition: M.K., R.C.R.P., and S.S.K.C.; supervision: S.S.K.C.

CONFLICTS OF INTEREST

The authors declare no competing interests.

ACKNOWLEDGMENTS

The authors would like to thank Robert Arpke, Christine Rohlf, Matthew Pappas, and Julia Karls for their help with animal husbandry, imaging, and discussion. The study was supported by a Regenerative Medicine Minnesota Discovery Science grant (RMM 102516 001), the National Institute of Arthritis and Musculoskeletal and Skin Diseases (R01 AR075413, R01 AR071439, and R01 AR078571), and University of Minnesota Startup and Children's Discovery-Winefest funds. The monoclonal antibodies to PAX7, embryonic MHC, MHC, MHC-I, MHC-IIa, and MHC-IIb were obtained from the Developmental Studies Hybridoma Bank developed under the auspices of the NICHD and maintained by the University of Iowa.

Received: January 21, 2021
Revised: October 21, 2021
Accepted: October 22, 2021
Published: November 18, 2021

REFERENCES

Al Tanoury, Z., Rao, J., Tassy, O., Gobert, B., Gapon, S., Garnier, J.M., Wagner, E., Hick, A., Hall, A., Gussoni, E., et al. (2020). Differentiation of the human PAX7-positive myogenic precursors/satellite cell lineage in vitro. Development *147*, dev187344.

Amoasii, L., Hildyard, J.C.W., Li, H., Sanchez-Ortiz, E., Mireault, A., Caballero, D., Harron, R., Stathopoulou, T.R., Massey, C., Shelton, J.M., et al. (2018). Gene editing restores dystrophin expression in a canine model of Duchenne muscular dystrophy. Science *362*, 86–91.

Arpke, R.W., Darabi, R., Mader, T.L., Zhang, Y., Toyama, A., Lonetree, C.L., Nash, N., Lowe, D.A., Perlingeiro, R.C., and Kyba, M. (2013). A new immuno-, dystrophin-deficient model, the NSG-mdx(4Cv) mouse, provides evidence for functional improvement following allogeneic satellite cell transplantation. Stem Cells *31*, 1611–1620.

Blau, H.M., and Daley, G.Q. (2019). Stem cells in the treatment of disease. N. Engl. J. Med. *380*, 1748–1760.

Brack, A.S., Bildsoe, H., and Hughes, S.M. (2005). Evidence that satellite cell decrement contributes to preferential decline in nuclear number from large fibres during murine age-related muscle atrophy. J. Cell Sci. *118*, 4813–4821.

Brack, A.S., Conboy, I.M., Conboy, M.J., Shen, J., and Rando, T.A. (2008). A temporal switch from notch to Wnt signaling in muscle stem cells is necessary for normal adult myogenesis. Cell Stem Cell *2*, 50–59.

Chal, J., Oginuma, M., Al Tanoury, Z., Gobert, B., Sumara, O., Hick, A., Bousson, F., Zidouni, Y., Mursch, C., Moncuquet, P., et al. (2015). Differentiation of pluripotent stem cells to muscle fiber to model Duchenne muscular dystrophy. Nat. Biotechnol. *33*, 962–969.

Chal, J., and Pourquié, O. (2017). Making muscle: skeletal myogenesis in vivo and in vitro. Development *144*, 2104–2122.

Chan, S.S., Arpke, R.W., Filareto, A., Xie, N., Pappas, M.P., Penaloza, J.S., Perlingeiro, R.C.R., and Kyba, M. (2018). Skeletal muscle stem cells from PSC-derived teratomas have functional regenerative capacity. Cell Stem Cell *23*, 74–85.

Chan, S.S., Shi, X., Toyama, A., Arpke, R.W., Dandapat, A., Iacovino, M., Kang, J., Le, G., Hagen, H.R., Garry, D.J., et al. (2013). Mesp1 patterns mesoderm into cardiac, hematopoietic, or skeletal myogenic progenitors in a context-dependent manner. Cell Stem Cell *12*, 587–601.

Charville, G.W., Cheung, T.H., Yoo, B., Santos, P.J., Lee, G.K., Shrager, J.B., and Rando, T.A. (2015). In vitro expansion and in vivo self-renewal of human muscle stem cells. Stem Cell Reports *5*, 621–632.

Collins, C.A., Olsen, I., Zammit, P.S., Heslop, L., Petrie, A., Partridge, T.A., and Morgan, J.E. (2005). Stem cell function, self-renewal, and behavioral heterogeneity of cells from the adult muscle satellite cell niche. Cell *122*, 289–301.

Cooper, R.N., Tajbakhsh, S., Mouly, V., Cossu, G., Buckingham, M., and Butler-Browne, G.S. (1999). In vivo satellite cell activation via Myf5 and MyoD in regenerating mouse skeletal muscle. J. Cell Sci. *112*, 2895–2901.

Cosgrove, B.D., Gilbert, P.M., Porpiglia, E., Mourkioti, F., Lee, S.P., Corbel, S.Y., Llewellyn, M.E., Delp, S.L., and Blau, H.M. (2014). Rejuvenation of the muscle stem cell population restores strength to injured aged muscles. Nat. Med. *20*, 255–264.

Darabi, R., Arpke, R.W., Irion, S., Dimos, J.T., Grskovic, M., Kyba, M., and Perlingeiro, R.C. (2012). Human ES- and iPS-derived myogenic progenitors restore DYSTROPHIN and improve contractility upon transplantation in dystrophic mice. Cell Stem Cell *10*, 610–619.

Filareto, A., Parker, S., Darabi, R., Borges, L., Iacovino, M., Schaaf, T., Mayerhofer, T., Chamberlain, J.S., Ervasti, J.M., McIvor, R.S., et al. (2013). An in vitro gene therapy approach to treat muscular dystrophy using inducible pluripotent stem cells. Nat. Commun. *4*, 1549.

Gilbert, P.M., Havenstrite, K.L., Magnusson, K.E., Sacco, A., Leonardi, N.A., Kraft, P., Nguyen, N.K., Thrun, S., Lutolf, M.P., and Blau, H.M. (2010). Substrate elasticity regulates skeletal muscle stem cell self-renewal in culture. Science *329*, 1078–1081.

Giordani, L., He, G.J., Negroni, E., Sakai, H., Law, J.Y.C., Siu, M.M., Wan, R., Corneau, A., Tajbakhsh, S., Cheung, T.H., et al. (2019). High-dimensional single-cell cartography reveals novel skeletal muscle-resident cell populations. Mol. Cell *74*, 609–621.

Günther, S., Kim, J., Kostin, S., Lepper, C., Fan, C.M., and Braun, T. (2013). Myf5-positive satellite cells contribute to Pax7-dependent long-term maintenance of adult muscle stem cells. Cell Stem Cell *13*, 590–601.

Gussoni, E., Pavlath, G.K., Lanctot, A.M., Sharma, K.R., Miller, R.G., Steinman, L., and Blau, H.M. (1992). Normal dystrophin transcripts detected in Duchenne muscular dystrophy patients after myoblast transplantation. Nature *356*, 435–438.

Hicks, M.R., Hiserodt, J., Paras, K., Fujiwara, W., Eskin, A., Jan, M., Xi, H., Young, C.S., Evseenko, D., Nelson, S.F., et al. (2018). ERBB3 and NGFR mark a distinct skeletal muscle progenitor cell in human development and hPSCs. Nat. Cell Biol. *20*, 46–57.

Ippolito, J., Arpke, R.W., Haider, K.T., Zhang, J., and Kyba, M. (2012). Satellite cell heterogeneity revealed by G-Tool, an open algorithm to quantify myogenesis through colony-forming assays. Skelet. Muscle *2*, 13.

Jiwlawat, N., Lynch, E., Jeffrey, J., Van Dyke, J.M., and Suzuki, M. (2018). Current progress and challenges for skeletal muscle differentiation from human pluripotent stem cells using transgene-free approaches. Stem Cells Int. *2018*, 6241681.

Karpati, G., Ajdukovic, D., Arnold, D., Gledhill, R.B., Guttmann, R., Holland, P., Koch, P.A., Shoubridge, E., Spence, D., Vanasse, M., et al. (1993). Myoblast transfer in Duchenne muscular dystrophy. Ann. Neurol. *34*, 8–17.

Konieczny, P., Swiderski, K., and Chamberlain, J.S. (2013). Gene and cell-mediated therapies for muscular dystrophy. Muscle Nerve *47*, 649–663.

Mauro, A. (1961). Satellite cell of skeletal muscle fibers. J. Biophys. Biochem. Cytol. *9*, 493–495.

Mendell, J.R., Kissel, J.T., Amato, A.A., King, W., Signore, L., Prior, T.W., Sahenk, Z., Benson, S., McAndrew, P.E., Rice, R., et al. (1995). Myoblast transfer in the treatment of Duchenne's muscular dystrophy. N. Engl. J. Med. *333*, 832–838.

Montarras, D., Morgan, J., Collins, C., Relaix, F., Zaffran, S., Cumano, A., Partridge, T., and Buckingham, M. (2005). Direct isolation of satellite cells for skeletal muscle regeneration. Science *309*, 2064–2067.

Parker, M.H., Loretz, C., Tyler, A.E., Duddy, W.J., Hall, J.K., Olwin, B.B., Bernstein, I.D., Storb, R., and Tapscott, S.J. (2012). Activation of Notch signaling during in vitro expansion maintains donor muscle cell engraftment. Stem Cells *30*, 2212–2220.

Périé, S., Trollet, C., Mouly, V., Vanneaux, V., Mamchaoui, K., Bouazza, B., Marolleau, J.P., Laforêt, P., Chapon, F., Eymard, B., et al. (2014). Autologous myoblast transplantation for oculopharyngeal muscular dystrophy: a phase I/IIa clinical study. Mol. Ther. *22*, 219–225.

Quarta, M., Brett, J.O., DiMarco, R., De Morree, A., Boutet, S.C., Chacon, R., Gibbons, M.C., Garcia, V.A., Su, J., Shrager, J.B., et al. (2016). An artificial niche preserves the quiescence of muscle stem cells and enhances their therapeutic efficacy. Nat. Biotechnol. *34*, 752–759.

Roth, S.M., Martel, G.F., Ivey, F.M., Lemmer, J.T., Metter, E.J., Hurley, B.F., and Rogers, M.A. (2000). Skeletal muscle satellite cell populations in healthy young and older men and women. Anat. Rec. *260*, 351–358.

Sacco, A., Doyonnas, R., Kraft, P., Vitorovic, S., and Blau, H.M. (2008). Self-renewal and expansion of single transplanted muscle stem cells. Nature *456*, 502–506.

Schultz, E., Gibson, M.C., and Champion, T. (1978). Satellite cells are mitotically quiescent in mature mouse muscle: an EM and radioautographic study. J. Exp. Zool. *206*, 451–456.

Seale, P., Asakura, A., and Rudnicki, M.A. (2001). The potential of muscle stem cells. Dev. Cell *1*, 333–342.

Seale, P., Bjork, B., Yang, W., Kajimura, S., Chin, S., Kuang, S., Scimè, A., Devarakonda, S., Conroe, H.M., Erdjument-Bromage, H., et al. (2008). PRDM16 controls a brown fat/skeletal muscle switch. Nature *454*, 961–967.

Shea, K.L., Xiang, W., LaPorta, V.S., Licht, J.D., Keller, C., Basson, M.A., and Brack, A.S. (2010). Sprouty1 regulates reversible quiescence of a self-renewing adult muscle stem cell pool during regeneration. Cell Stem Cell *6*, 117–129.

Shelton, M., Metz, J., Liu, J., Carpenedo, R.L., Demers, S.P., Stanford, W.L., and Skerjanc, I.S. (2014). Derivation and expansion of PAX7-positive muscle progenitors from human and mouse embryonic stem cells. Stem Cell Reports *3*, 516–529.

Skuk, D., Goulet, M., Roy, B., Chapdelaine, P., Bouchard, J.P., Roy, R., Dugré, F.J., Sylvain, M., Lachance, J.G., Deschênes, L., et al. (2006). Dystrophin expression in muscles of duchenne muscular dystrophy patients after high-density injections of normal myogenic cells. J. Neuropathol. Exp. Neurol. *65*, 371–386.

Skuk, D., Goulet, M., Roy, B., Piette, V., Côté, C.H., Chapdelaine, P., Hogrel, J.Y., Paradis, M., Bouchard, J.P., Sylvain, M., et al. (2007). First test of a "high-density injection" protocol for myogenic cell transplantation throughout large volumes of muscles in a Duchenne muscular dystrophy patient: eighteen months follow-up. Neuromuscul. Disord. *17*, 38–46.

Snow, M.H. (1977). Myogenic cell formation in regenerating rat skeletal muscle injured by mincing. II. An autoradiographic study. Anat. Rec. *188*, 201–217.

Stuelsatz, P., Shearer, A., Li, Y., Muir, L.A., Ieronimakis, N., Shen, Q.W., Kirillova, I., and Yablonka-Reuveni, Z. (2015). Extraocular muscle satellite cells are high performance myo-engines retaining efficient regenerative capacity in dystrophin deficiency. Dev. Biol. *397*, 31–44.

Tierney, M.T., Gromova, A., Sesillo, F.B., Sala, D., Spenlé, C., Orend, G., and Sacco, A. (2016). Autonomous extracellular matrix remodeling controls a progressive adaptation in muscle stem cell regenerative capacity during development. Cell Rep. *14*, 1940–1952.

von Maltzahn, J., Jones, A.E., Parks, R.J., and Rudnicki, M.A. (2013). Pax7 is critical for the normal function of satellite cells in adult skeletal muscle. Proc. Natl. Acad. Sci. U S A *110*, 16474–16479.

Zammit, P.S., Golding, J.P., Nagata, Y., Hudon, V., Partridge, T.A., and Beauchamp, J.R. (2004). Muscle satellite cells adopt divergent fates: a mechanism for self-renewal? J. Cell Biol. *166*, 347–357.

Stem Cell Reports, Volume *16*

Supplemental Information

In vitro expanded skeletal myogenic progenitors from pluripotent stem cell-derived teratomas have high engraftment capacity

Ning Xie, Sabrina N. Chu, Karim Azzag, Cassandra B. Schultz, Lindsay N. Peifer, Michael Kyba, Rita C.R. Perlingeiro, and Sunny S.K. Chan

Supplemental Figures

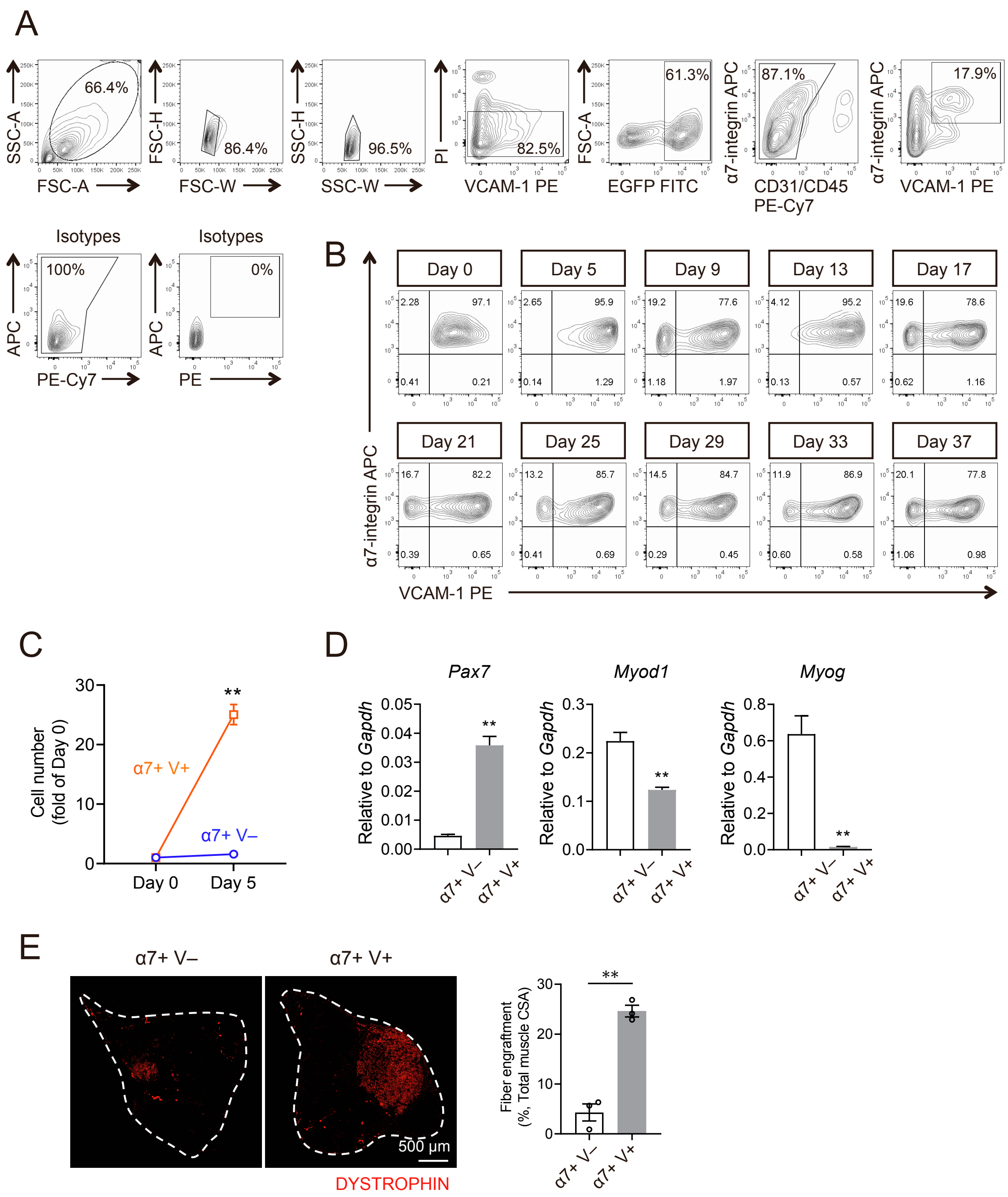

Figure S1, related to Figure 1. FACS profiling and comparison between α7+ VCAM+ and α7+ VCAM– populations in expanded teratoma-derived skeletal myogenic progenitors

(A) Representative FACS gatings for identifying skeletal myogenic progenitors in mouse ESC-derived teratomas.

(B) FACS profiling of teratoma-derived skeletal myogenic progenitors in cultures over 37 days. Note that the FACS plots for Day 0 and Day 37 were also used in Figure 1C as “Fresh” and “Expanded” respectively.

(C) α7+ V+ and α7+ V– cells were sorted from expanded (day 37/passage 8) teratoma-derived skeletal myogenic progenitors and were subsequently cultured for another 5 days. α7+ V+ cells grew more readily than α7+ V– cells. Data are shown as mean ± SEM from 3 independent experiments. **$p < 0.01$.

(D) Quantitative RT-PCR analysis for markers of muscle stem cells (*Pax7*), committed myogenic cells (*Myod1*) and differentiated myocytes (*Myog*) in α7+ V+ and α7+ V– cells sorted from expanded (day 37/passage 8) teratoma-derived skeletal myogenic progenitors. Data are shown as mean ±SEM from 3 independent experiments. **$p < 0.01$.

(E) Representative images (left) and quantification (right) of fiber engraftment (DYSTROPHIN+ fibers) of α7+ V+ and α7+ V– cells sorted from expanded (day 37/passage 8) teratoma-derived skeletal myogenic progenitors. Data are shown as mean ± SEM from 3 biological replicates. **$p < 0.01$.

ESC, embryonic stem cells. α7, α7-integrin. V, VCAM-1. CSA, cross-sectional area.

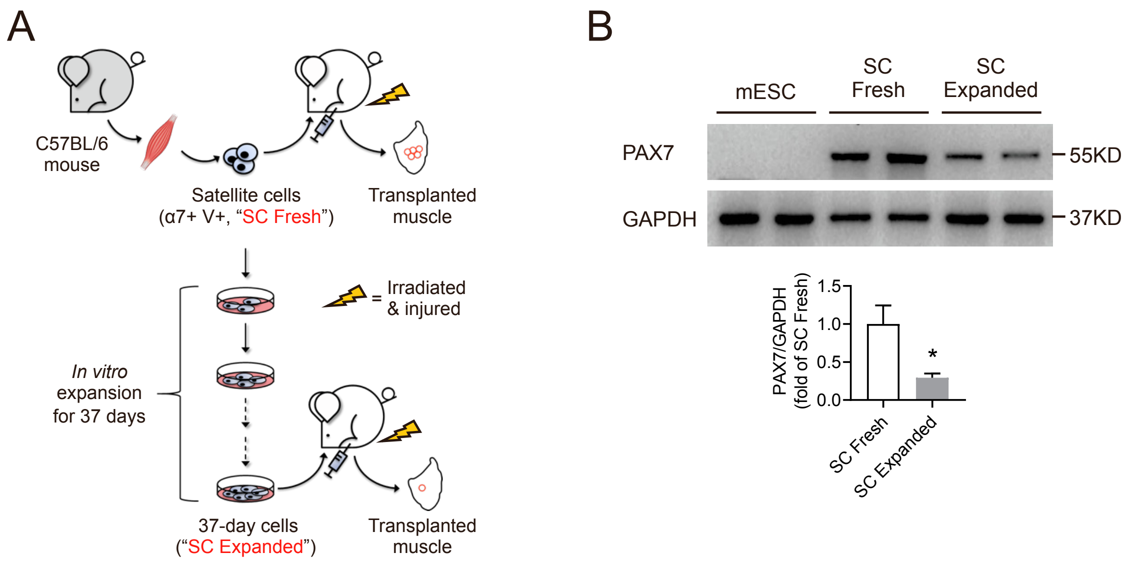

Figure S2, related to Figures 2 and 3. Isolation and characterization of adult satellite cells

(A) Schematic of *in vitro* expansion of endogenous satellite cells isolated from adult muscle tissues.

(B) Immunoblots (top) and quantification (bottom) of PAX7 protein expression in freshly-isolated and expanded adult satellite cells. Mouse ESCs were used as a control. Data are shown as mean ±SEM from 4 independent experiments. *$p < 0.05$.

α7, α7-integrin. V, VCAM-1. mESC, mouse embryonic stem cells. SC, satellite cells.

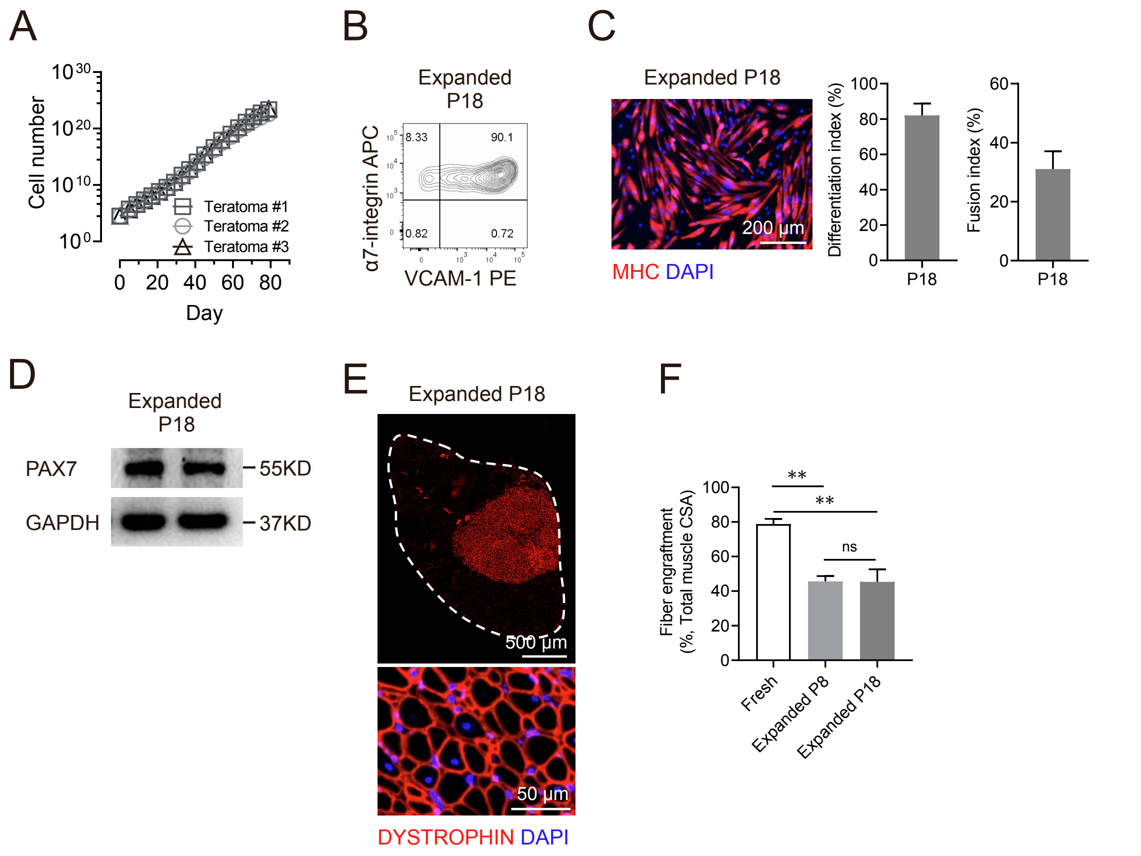

Figure S3, related to Figure 3. Teratoma-derived skeletal myogenic progenitors remain engraftable after 79 days (passage 18) of *in vitro* expansion

(A) *In vitro* cultures of teratoma-derived skeletal myogenic progenitors grew steadily for up to 79 days (passage 18). Data are shown as mean ±SEM from 3 independent experiments.

(B) FACS profile of expanded passage 18 (P18) teratoma-derived skeletal myogenic progenitors.

(C) Immunostaining (left) of MHC in expanded P18 cells cultured in differentiation medium. Scale bar represents 200 μm. Quantification of differentiation (middle) and

fusion in MHC+ myotubes (right) from 3 independent experiments. Data are shown as mean ± SEM.

(D) Immunoblots showing that expanded P18 cells expressed the muscle stem cell transcription factor PAX7 (2 independent samples from each cell group are shown).

(E) Expanded P18 teratoma-derived skeletal myogenic progenitors engrafted and formed DYSTOPHIN+ fibers 4 months post-transplant. The whole TA muscles are outlined (top, scale bar represents 500 μm), and magnified images are shown (bottom, scale bar represents 50 μm).

(F) Quantitation of fiber engraftment (DYSTROPHIN+ fibers) in TA muscles transplanted with freshly-sorted, expanded P8 and expanded P18 skeletal myogenic progenitors derived from teratomas (n=9-12 biological replicates). Data are shown as mean ± SEM. ∗∗$p < 0.01$. ns, not significant. Note that the quantification for "Fresh" and "Expanded P8" were also used in Figure 3C as "Fresh, teratoma-derived" and "Expanded, teratoma-derived", respectively.

MHC, myosin heavy chain. PI, propidium iodide. CSA, cross-sectional area.

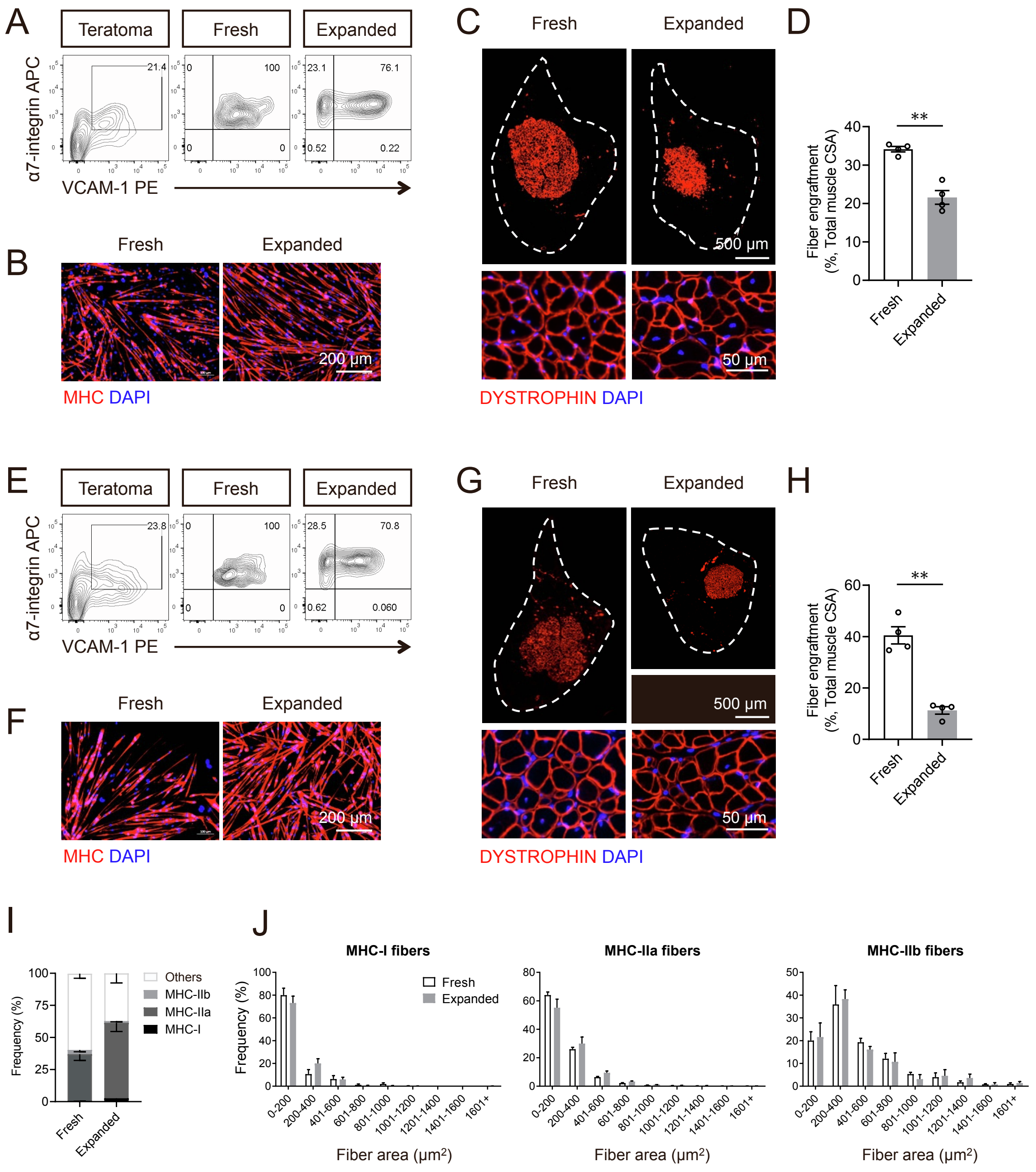

Figure S4, related to Figure 3. Evaluation of the functionality of expanded teratoma-derived skeletal myogenic progenitors generated from multiple PSC lines

C57BL/6N-PRX-B6N #1 ESCs (A-D) and Pax7-ZsGreen iPSCs (E-H) were used to generate teratoma-derived α7+ V+ cells, whose *in vitro* expandability and skeletal myogenic potential were evaluated.

(A, E) FACS profiling of total teratoma cells, freshly-sorted α7+ V+ cells and expanded 37-day (passage 8) cells.

(B, F) Immunostaining of MHC in freshly-sorted and expanded cells cultured in differentiation medium. Scale bar represents 200 μm.

(C, G) Freshly-sorted and expanded teratoma-derived skeletal myogenic progenitors engrafted and formed DYSTOPHIN+ fibers 6 weeks post-transplant. The whole TA muscles are outlined (top, scale bar represents 500 μm), and magnified images are shown (bottom, scale bar represents 50 μm).

(D, H) Quantitation of fiber engraftment (DYSTROPHIN+ fibers) (n=4 biological replicates). Data are shown as mean ± SEM. **$p < 0.01$.

(I) Quantification of various fiber types of engrafted DYSTROPHIN+ muscle fibers derived from freshly-sorted and expanded teratoma-derived skeletal myogenic progenitors. Data are shown as mean ± SEM from 6 biological replicates.

(J) Area distribution of individual DYSTROPHIN+ fibers expressing MHC-I (left), MHC-IIa (middle) and MHC-IIb (right). Data are shown as mean ± SEM from 6 biological replicates.

ESC, embryonic stem cells. iPSC, induced pluripotent stem cells. α7, α7-integrin. V, VCAM-1. MHC, myosin heavy chain. CSA, cross-sectional area.

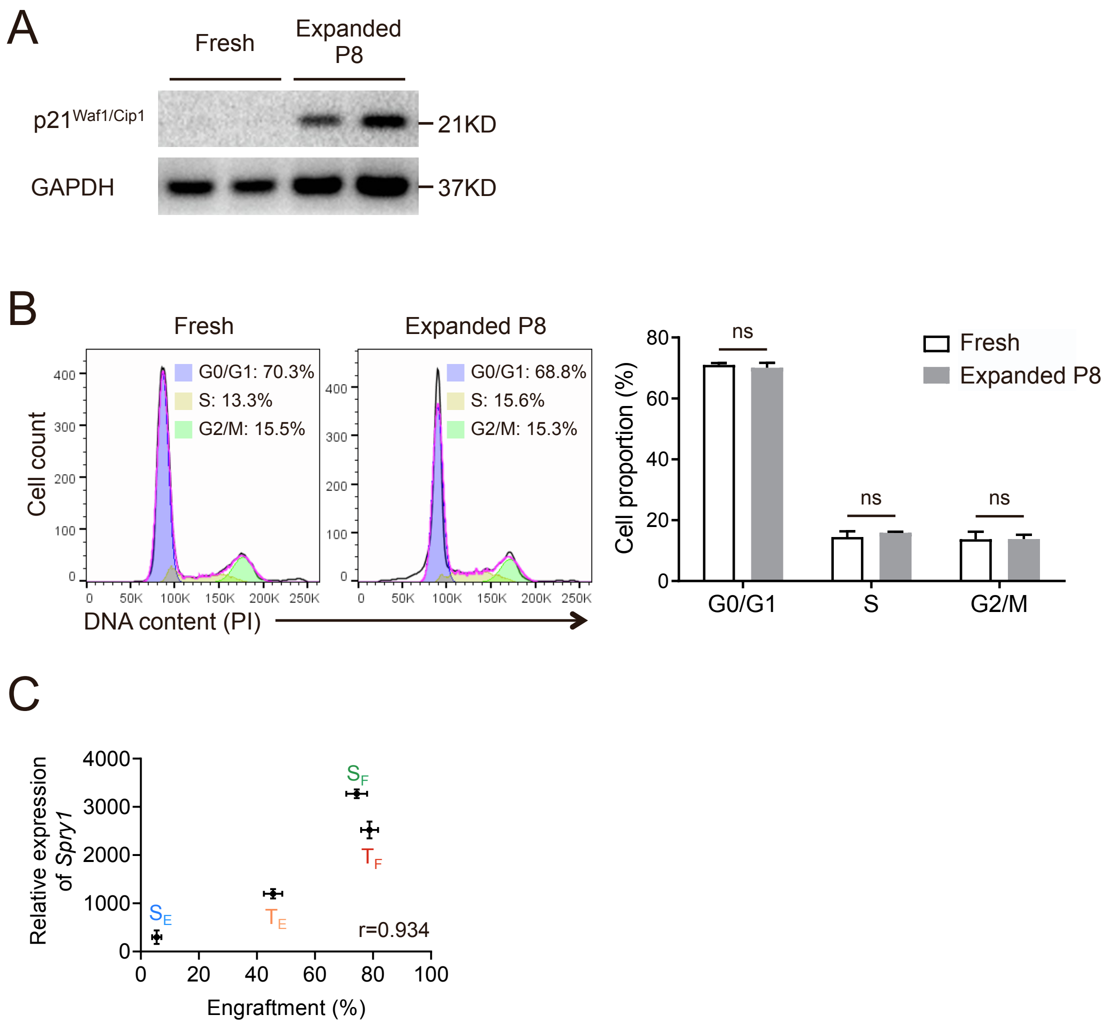

Figure S5, related to Figure 5. Potential factors affecting engraftment

(A) Immunoblots showing $p21^{Waf1/Cip1}$ protein expression in expanded P8 cells but not in fresh cells (2 independent samples from each cell group are shown).

(B) FACS profiling (left) and quantification (right) of cell cycle analysis of fresh and expanded P8 cells. Cells were stained with PI and analyzed by FACS. Data are shown as mean ± SEM of 3 independent experiments. ns, not significant.

(C) Pearson correlation coefficient (r) between engraftment (%) and *Spry1* gene expression (MRN-normalized read counts) from S_E, T_E, T_F and S_F samples.

Supplemental Tables

Table S1, related to Figure 5. Common differentially expressed genes in engraftable cells vs. non-engraftable cells

Table S2, related to Figure 5. Pearson correlation analysis of differentially expressed genes

Supplemental Experimental Procedures

Animals

Animal housing, husbandry and experiments were carried out according to protocols (#1903-36840A) approved by the University of Minnesota Institutional Animal Care and Use Committee and under institutional assurances of AAALAC accreditation (#000552, as of Nov 2015), USDA research facility registration (USDA No. 41-R-0005), and PHS Animal Welfare Assurance approval (A3456-01). The NSG-mdx^{4Cv} mice has been described (Arpke et al., 2013), and were generated by crossing NOD.Cg-Prkdc^{scid}Il2rg^{tm1Wjl}/SzJ (NSG) mice and B6Ros.Cg-Dmd$^{mdx\text{-}4Cv}$/J (mdx^{4Cv}) mice and maintained in autoclaved cages. NSG-mdx^{4Cv} mice of both sexes (3-4 months old) were used for experiments. Male C57BL/6J mice (3-4 months old) were obtained from Jackson Laboratory (#000664, Bar Harbor, ME).

ESCs/iPSCs culture

E14-EGFP mouse ESCs (Ismailoglu et al., 2008), C57BL/6N-PRX-B6N #1 mouse ESCs (Jackson Laboratory #012448, via Mouse Genetics Laboratory, University of Minnesota, MN) and Pax7-ZsGreen mouse iPSCs (Chan et al., 2018) were cultured on irradiated mouse embryonic fibroblasts (MEFs) in maintenance medium containing Knock-Out Dulbecco's Minimum Essential Medium (DMEM) (Life Technologies #10829-018, Grand Island, NY), 15% fetal bovine serum (FBS) (Gemini Bio-Products #100-119, West Sacramento, CA), 1% non-essential amino acids (Life Technologies #11140-050), 1% penicillin/streptomycin (P/S) (Life Technologies #15140-122), 2 mM Glutamax (Life Technologies #SCR006), 0.1 mM β-mercaptoethanol (Sigma #M3148, St. Louis, MO)

and 500 U/mL leukemia inhibitory factor (Millipore #ESG1107, Temecula, CA), at 37°C in 5% CO_2. Cells were fed with fresh medium every day and were passaged with 0.25% trypsin-EDTA (Life Technologies #25200-072) every other day. On the day of teratoma formation, ESCs were dissociated with 0.25% trypsin-EDTA and plated on a cell culture flask for 45-60 min to remove MEFs.

Teratoma formation

Mouse ESCs/iPSCs were injected into tibialis anterior (TA) muscles of NSG-mdx^{4Cv} mice to generate teratomas. Two days prior to cell injections, recipients were anesthetized with ketamine (150 mg/kg, i.p., Akorn #NDC:59399-114-10, Lake Forest, IL) and xylazine (10 mg/kg, i.p., Akorn #NDC:59399-111-50), and left hindlimbs were exposed to 1200 cGy X-ray irradiation. One day before cell injections, 15 µL of cardiotoxin (10 µM, Latoxan #L8102, France) was injected into the irradiated muscles to induce muscle injury. The next day, 250,000 ESCs or 1,000,000 iPSCs were resuspended in 10 µL PBS (HyClone #SH30256.01, Logan, UT) and injected into the TA muscles using a Hamilton syringe (Hamilton, Reno, NV).

Teratoma harvest

Four week-old teratomas were dissected and mechanically chopped into small pieces. Chopped tissues were then incubated in primary digestion buffer consisting of DMEM/high glucose (HyClone #SH30243.01), 2 mg/mL Collagenase II (Gibco #17101-015, Gaithersburg, MD) and 1% P/S on a shaker at 250 rpm, 37°C for 30 min. The digested cell supernatant was filtered through 40 µm cell strainers and spun down, then

the filtered cell pellet was washed with rinsing buffer consisting of Ham's/F-10 medium (Caisson Labs #HFL01, Smithfield, UT), 10% horse serum (HyClone #SH30074.03), 1% HEPES buffer solution (Caisson Labs #HOL06) and 1% P/S and kept on ice (first-pass). The undigested chunk tissues were further incubated in primary digestion buffer on a shaker at 250 rpm, 37°C for another 30 min, then filtered through 40 μm cell strainers, spun down and the cell pellet was washed with rinsing buffer (second-pass). The cells from first-pass and second-pass were combined together, resuspended in FACS buffer (PBS with 0.2% FBS) and kept on ice until downstream FACS staining.

Satellite cells isolation

Hindlimb muscles were removed from C57BL/6J mice and then mechanically chopped into small pieces. Chopped tissues were incubated in primary digestion buffer on a shaker at 250 rpm, 37°C for 75 min. Primary digestion was stopped by adding rinsing buffer and tissue spun down at 1500 rpm for 5 min at 4°C. The tissues were further digested in secondary digestion buffer consisting of rinsing buffer supplemented with 0.1 mg/mL Collagenase II and 0.5 mg/mL Dispase (Gibco, Cat#17105-041) on a shaker at 250 rpm, 37°C for 30 min. Digested tissues were vortexed, drawn and released into a 10 mL syringe with a 16-gauge needle four times and then with an 18-gauge needle four times. Dissociated tissues were filtered through 40 μm cell strainers, spun down, then washed with rinsing buffer and resuspended in FACS buffer on ice. Cells were stained and sorted as described below and endogenous satellite cells are defined as CD31– CD45– α7-integrin+ VCAM-1+.

Cell isolation from transplanted TA muscles

Transplanted TA muscles were removed and were processed the same way as satellite cells isolation.

Cell transplantation

NSG-mdx^{4Cv} mice were irradiated and cardiotoxin-injured as mentioned above. For cell transplantations, 40,000 cells (derived from teratomas or isolated from adult muscles) in PBS was injected into TA muscles using a Hamilton syringe. Grafted TA muscles were harvested at 4 months after transplantation and processed for immunostaining or fluorescence activated cell sorting (FACS) analysis.

Fluorescence-activated cell sorting (FACS)

Dissociated cells were filtered through 40 μm cell strainers and incubated with fluorophore-conjugated antibodies on ice for 30 min. Stained cells were washed twice in FACS buffer (PBS with 0.2% FBS) and resuspended in FACS buffer with propidium iodide (PI, 1 μg/mL, Sigma #P4170) for FACS analysis. PI was used as a live/dead cell indicator and only live cells (PI–) were counted. Cells were analyzed and sorted by BD FACSAriaII (BD Biosciences, San Diego, CA) with FACSDiva software (BD Biosciences). Four-way purity precision was used for bulk sorting and single-cell precision was used for sorting single cells into 96-well plate for clonal analysis. Sorted cells were collected into cell culture medium and kept cold until downstream processing. FACS plots were generated using FlowJo (FLOWJO LLC, Ashland, OR). Antibodies used were PE-Cy7 anti-CD31, BD Biosciences #561410; RRID:AB_10612003, San Jose,

CA; PE-Cy7 anti-CD45, BD Biosciences #552848; RRID:AB_394489; APC anti-α7-Integrin, AbLab #67-0010-05, Vancouver, Canada; Biotin anti-VCAM-1, BD Biosciences #553331; RRID:AB_10053328; PE streptavidin, BD Biosciences #554061; all at 0.5 µL per million cells. To analyze cell-cycle progression, cells were harvested and immobilized in 70% ethanol at 4°C for 12 hr, followed by washing with PBS. Then the cells were incubated in PI (50 µg/mL) with RNase (10 µg/mL) for 30 min. 10,000 cells were analyzed for each sample using FACSAriaII.

In vitro cell expansion

To expand the teratoma-derived skeletal myogenic progenitors, FACS-sorted CD31– CD45– α7+ VCAM+ cells were plated on 0.1% gelatin-coated wells and cultured in myogenic expansion medium containing Ham's/F-10, 20% FBS (Sigma #F0926), 10 ng/mL basic FGF (R&D Systems #233-FB/CF, Minneapolis, MN), 1% P/S, 2 mM Glutamax and 0.1 mM β-mercaptoethanol. Cells were passaged every 4-5 days in myogenic expansion medium when they reached 40% to 50% confluency. Cells (freshly-isolated or expanded) were plated in myogenic expansion medium and cultured for 3 days before processing to immunostaining with PAX7 and MYOD1 and imaging. Endogenous satellite cells were cultured and expanded similarly.

In vitro skeletal myogenic differentiation

Skeletal myogenic progenitors were differentiated into myotubes at 70% to 80% confluency. Medium was switched to myogenic differentiation medium containing high-glucose DMEM, 2% horse serum, 1% insulin-transferrin-selenium (Life Technologies

#41400045) and 1% P/S for 3 days. Cells at the end of differentiation were subjected to immunostaining with myosin heavy chain (MHC). Cells were imaged and analyzed. Differentiation index was calculated as the percentage of nuclei within MHC+ cells relative to the total number of nuclei. Fusion index was calculated as the percentage of nuclei within myotubes (elongated MHC+ cells containing at least three nuclei) relative to the total number of nuclei. Approximately 100 myotubes were counted for each replicate.

Clonal analysis

Single cells were seeded via FACS into gelatin-coated 96-well plates (one cell per well) in myogenic clonal medium containing DMEM/F12 (Cellgro #15-090-CV, Manassas, VA), 20% FBS, 10% horse serum, 10 ng/mL basic FGF, 1% P/S, 2 mM Glutamax and 0.5% chick embryo extract (US Biological #C3999, Salem, MA). This medium supports both proliferation and differentiation (Ippolito et al., 2012). Cells were cultured at 37 °C, 5% CO_2 in a humidified tissue culture incubator. Cells were left undisturbed for 8 days, followed by immunostaining with MHC and 4’,6-diamidino-2-phenylindole (DAPI) (Life Technologies #D3571). Clone size was determined by counting the number of DAPI-positive nuclei using ImageJ software.

Western blotting

One hundred thousand cells were used for each lane. Protein extracts were separated by electrophoresis on 10% SDS-polyacrylamide gels and then transferred to PVDF membranes. Membranes were blocked with 5% nonfat dry milk in Tris-buffered saline

and 0.1% Tween 20 (Bio-Rad #170-6531, Hercules, CA) for 1 hr, and then incubated with the primary antibody overnight at 4 °C, followed by incubation with secondary antibody peroxidase-conjugated anti-mouse IgG (1:10000, GE Healthcare # NA931, RRID:AB_772210) for 1 hr. Detections were carried out using a chemiluminescence detection substrate (Thermo Fisher Scientific #32106). Primary antibodies used were mouse anti-PAX7 at 1:10, Developmental Studies Hybridoma Bank (DSHB) #PAX7, RRID:AB_528428, Iowa City, IA; mouse anti-p21$^{WAF1/CIP1}$ at 1:1000, Santa Cruz Biotechnology Cat# sc-6246, RRID:AB_628073, Dallas, TX; and HRP-conjugated GAPDH at 1:10000, Proteintech #HRP-60004, RRID:AB_2737588, Rosemont, IL.

Immunostaining on cultured cells

Cell cultures were fixed with 4% paraformaldehyde (PFA) (Sigma #P6148) for 20 min, rinsed three times in PBS, then permeabilized with 0.3% Triton X-100 (Sigma #X100) for 30 min, followed by blocking with 3% bovine serum albumin (BSA) (Thermo Fisher Scientific #BP1605-100, Waltham, MA) for 1 hr, all at room temperature. Primary antibodies were diluted in 3% BSA and incubated overnight at 4°C. Cultures were then washed three times in PBS and incubated with appropriate coupled secondary antibodies in 3% BSA for 1 hr at room temperature. Cultures were counterstained with DAPI for 15 min and washed 3 times in PBS before analysis. Primary antibodies used were mouse anti-PAX7 at 1:10, DSHB #PAX7, RRID:AB_528428; rabbit anti-MYOD1 at 1:500, Santa Cruz Biotechnology #sc-304, RRID:AB_631992, Dallas, TX; and mouse anti-MHC at 1:20, DSHB #MF-20, RRID:AB_2147781. Secondary antibodies used were: goat anti-mouse Alexa Fluor 555, goat anti-mouse Alexa Fluor 647, goat anti-rabbit

Alexa Fluor 647 (all from Life Technologies). Fluorescent images were captured with a Zeiss AxioObserver Z1 inverted microscope with an AxioCamMR3 camera using the ZEN software (Zeiss). Image processing and quantification were performed using Fiji/ImageJ.

Immunostaining on muscle sections

TA muscles were harvested for analysis 4 months after cell transplantation. Harvested TA muscles were embedded in optimal cutting temperature (OCT) solution (Scigen #4586, Gardena, CA), snap frozen in liquid nitrogen-chilled 2-methylbutane (Sigma #320404) and stored at -80°C. For EGFP staining, TA muscles were pre-fixed with 4% PFA overnight at 4°C following by overnight incubation with 20%/30% sucrose before embedding. Muscle sections were cut at 10 μm on a Leica CM3050 S cryostat (Leica Microsystems, Buffalo Grove, IL) and collected every other 250 μm across the TA muscle. Sections with the largest cross-sectional area of the whole TA were used for fiber engraftment evaluation. Sections were rehydrated with PBS, permeabilized with 0.3% Triton X-100 for 30 min and blocked with 3% BSA for 1 hr at room temperature. For PFA-fixed samples, sections were boiled in sodium citrate buffer (10 mM sodium citrate, 0.05% Tween 20, pH 6.0) at 95°C for 20 min for epitope retrieval before permeabilization. Primary antibodies were incubated overnight at 4°C followed by secondary antibodies for 1 hr at room temperature and counterstained with DAPI. Slides were mounted with Immu-Mount (Thermo Scientific #9990402) and proceeded to image capture as described above. Images showing whole TA muscles were captured in a tile mode and stitched together using the Zen software. Primary antibodies used were rabbit

anti-DYSTROPHIN (1:250, Abcam #ab15277; RRID:AB_301813), mouse anti-MHC-I (1:100, DSHB #BA-D5; RRID:AB_2235587), mouse anti-MHC-IIa (1:100, DSHB #SC-71; RRID:AB_2147165), mouse anti-MHC-IIb (1:100, DSHB #BF-F3; RRID:AB_2266724), mouse anti-PAX7 (1:10, DSHB #PAX7; RRID:AB_528428), chicken anti-EGFP (1:500, Abcam #ab13970; RRID:AB_300798), rabbit anti-laminin (1:500, Sigma #L9393; RRID:AB_477163), and Alexa Fluor 555 anti-α-bungarotoxin (1:100, Invitrogen #B35451; RRID:AB_2617152). Secondary antibodies used were goat anti-rabbit Alexa Fluor 555, goat anti-mouse Alexa Fluor 647 and donkey anti-chicken Alexa Fluor 488.

Fiber counting and area measurement

Sections with the largest cross-sectional area of the whole TA were used for fiber engraftment evaluation. Fiber counting and cross section area measurement were performed using Muscle2Veiw, a CellProfiler pipeline (Sanz et al., 2019) with adjusted parameters. DYSTROPHIN or laminin staining was used to determine the cross-sectional area of muscle fibers. Fiber engraftment is defined as the total cross-sectional area of DYSTROPHIN+ fibers over the total cross-sectional area of the whole TA section.

In situ muscle function assay

For functional evaluation of TA muscles, an *in situ* assessment of force production was performed using the Aurora 3-in-1 animal system (1300A; Aurora Scientific, Aurora, Canada), implemented with Dynamic Muscle Analysis software (615A; Aurora Scientific) for data analysis (Wu et al., 2021). Mice were maintained under anesthetized

during the whole procedure with isoflurane (Piramal Critical Care, NDC:66794-013-25, Mumbai, India). 4-5% of isoflurane were provided to the mice in the induction chamber until the mouse was recumbent, then the anesthesia was maintained with 1-2% isoflurane provided through a nose cone. The TA muscle was exposed and isolated at its distal tendon. The animal was then positioned on the aurora system and its knee was stabilized using a vertical knee pin. The TA distal tendon was attached to a force transducer and a pair of platinum-coated electrodes was placed inside the TA muscle for electrical stimulation using rectangular unipolar pulses at 0.2 ms duration and 150 Hz. TA muscle was maintained at optimal length, and the maximal tetanic force (F_0) was determined at 0.2 ms of 150 Hz stimulation for 300 ms. Fatigue time was defined as the time required for force to drop to 30% of F_0 after stimulation at 0.2 ms of 150 Hz for 2 min. Total muscle cross-sectional area (CSA) was calculated by dividing muscle mass (mg) by the product of muscle length (mm) and muscle density (1.06 mg/mm^3). Specific force (sF_0) was determined by normalizing F_0 to CSA.

Quantitative RT-PCR

Total RNA was extracted using RNeasy Mini Kit (QIAGEN #74106, Valencia, CA), and subsequent genomic DNA removal and reverse transcription (RT) were performed using Verso cDNA Synthesis Kit (Thermo Scientific #AB1453A, Pittsburgh, PA). Quantitative reverse transcription polymerase chain reaction (qRT-PCR) was performed in triplicate using TB Green Premix Ex Taq II (Takara Bio, Otsu, Japan). Expression of individual genes was subsequently analyzed by the ΔCt method in relative to the expression of the housekeeping gene *Gapdh* in a QuantStudio 6 Flex Real-Time PCR System using

QuantStudio Real-Time PCR Software (both Applied Biosystems). Primer sequences are listed as follows: *Pax7* (GTTCGGGAAGAAAGAGGACGAC, GGTTCTGATTCCACATCTGAGCC); *Myod1* (GCACTACAGTGGCGACTCAGAT, TAGTAGGCGGTGTCGTAGCCAT); *Myog* (CCATCCAGTACATTGAGCGCCT, CTGTGGGAGTTGCATTCACTGG); *Gapdh* (TTCAACGGCACAGTCAAG, CCAGTAGACTCCACGACATA).

RNA-seq

Freshly-sorted α7+ V+ cells from E14 ESC-derived teratomas (T_F), 37-day expanded teratoma-derived cells (T_E), freshly-sorted α7+ V+ satellite cells from wildtype B6 hindlimb muscles (S_F) and 37-day expanded satellite cells (S_E) were collected for RNA extraction (200,000-500,000 cells per sample, n=2 biological replicates for each group). Total RNA was extracted using RNeasy Mini Kit and in-column genomic DNA removal was applied. RNA samples with RNA integrity number (RIN) > 7 were processed for library generation. 250 pg-10 ng of total RNA was used for sequencing libraries generation using SMARTer Stranded Total RNA-Seq Kit v2 – Pico Input Mammalian Kit (Clontech #634412). Paired-end 150 base-pair sequencing was performed using an Illumina NovaSeq 6000 (Illumina, San Diego, CA), producing 20 million raw reads per sample at the University of Minnesota Genomic Center.

RNA-seq analysis

RNA-seq raw paired-end reads were aligned to Mus musculus reference transcriptome (GRCm38/mm10) using Kallisto (v0.44.0) (Bray et al., 2016) via Minnesota

Supercomputing Institute, University of Minnesota. Transcript abundance was quantified and summarized into gene level as read counts values using the R package tximport (v1.10.0) (Soneson et al., 2015). DEBrowser software (Kucukural et al., 2019) implemented with DESeq2 (Love et al., 2014) was used to perform subsequent analysis. Lowly-covered genes were filtered out from the 8 samples (only genes with at least 2 read counts per million mapped reads in at least 2 samples were included), and the read counts of filtered genes were normalized using the median ratio normalization (MRN) method for further analysis. Principal component analysis (PCA) of all detected genes was plotted using the function PCA plot. Differentially expressed genes (DEGs) were identified with fold change > 1.25 and adjusted p value < 0.05. Heatmap of DEGs was generated using the function Heatmap plot included with DEBrowser. RNA-seq datasets can be accessed on GEO: GSE182508. Pearson correlation coefficient (r) was calculated between engraftment (%) and gene expression of 4 groups to evaluate correlation.

Software

FACS analysis data acquisition were performed in FACSDiva v6.1.3 (BD) and analyzed in FlowJo v7.6.3 (FLOWJO LLC). Immunostaining images were acquired using ZEN v2.3 pro (Zeiss). Fiber counting and measurements were performed with using Muscle2Veiw, a CellProfiler pipeline (Sanz et al., 2019).

Statistical analysis

Data are presented as mean ± SEM. All statistical analyses are performed using GraphPad Prism v6.07 (GraphPad Software, La Jolla, CA). Significance is calculated

using two-tailed, unpaired Student's t tests for comparison between two groups. Differences are considered to be statistically significant at the $p < 0.05$ level.

Supplemental References

Arpke, R.W., Darabi, R., Mader, T.L., Zhang, Y., Toyama, A., Lonetree, C.L., Nash, N., Lowe, D.A., Perlingeiro, R.C., and Kyba, M. (2013). A new immuno-, dystrophin-deficient model, the NSG-mdx(4Cv) mouse, provides evidence for functional improvement following allogeneic satellite cell transplantation. Stem Cells *31*, 1611-1620.

Bray, N.L., Pimentel, H., Melsted, P., and Pachter, L. (2016). Near-optimal probabilistic RNA-seq quantification. Nat. Biotechnol. *34*, 525-527.

Chan, S.S., Arpke, R.W., Filareto, A., Xie, N., Pappas, M.P., Penaloza, J.S., Perlingeiro, R.C.R., and Kyba, M. (2018). Skeletal muscle stem cells from PSC-derived teratomas have functional regenerative capacity. Cell Stem Cell *23*, 74-85.

Ippolito, J., Arpke, R.W., Haider, K.T., Zhang, J., and Kyba, M. (2012). Satellite cell heterogeneity revealed by G-Tool, an open algorithm to quantify myogenesis through colony-forming assays. Skelet. Muscle *2*, 13.

Ismailoglu, I., Yeamans, G., Daley, G.Q., Perlingeiro, R.C., and Kyba, M. (2008). Mesodermal patterning activity of SCL. Exp. Hematol. *36*, 1593-1603.

Kucukural, A., Yukselen, O., Ozata, D.M., Moore, M.J., and Garber, M. (2019). DEBrowser: interactive differential expression analysis and visualization tool for count data. BMC Genomics *20*, 6.

Love, M.I., Huber, W., and Anders, S. (2014). Moderated estimation of fold change and dispersion for RNA-seq data with DESeq2. Genome Biol. *15*, 550.

Sanz, G., Martínez-Aranda, L.M., Tesch, P.A., Fernandez-Gonzalo, R., and Lundberg, T.R. (2019). Muscle2View, a CellProfiler pipeline for detection of the capillary-to-

muscle fiber interface and high-content quantification of fiber type-specific histology. J. Appl. Physiol. *127*, 1698-1709.

Soneson, C., Love, M.I., and Robinson, M.D. (2015). Differential analyses for RNA-seq: transcript-level estimates improve gene-level inferences. F1000Research *4*, 1521.

Wu, J., Matthias, N., Bhalla, S., and Darabi, R. (2021). Evaluation of the Therapeutic Potential of Human iPSCs in a Murine Model of VML. Mol. Ther. *29*, 121-131.
